# Grass carp reovirus activates fish Ppp1r3g to suppress innate immunity and exacerbate hemorrhage via the 14-3-3ζ/Itgb3 signaling axis

**DOI:** 10.1371/journal.ppat.1014590

**Published:** 2026-09-18

**Authors:** Ruyi Wang, Man Zhou, Yuxuan Wang, Xudong Hu, Han Zhang, Hong Cao

**Affiliations:** 1 Institute of Hydrobiology, Chinese Academy of Sciences, Wuhan, China; 2 University of Chinese Academy of Sciences, Beijing, China; 3 Fisheries College, Hunan Agricultural University, Changsha, China; University of Arkansas for Medical Sciences, UNITED STATES OF AMERICA

## Abstract

Protein phosphatase 1 regulatory subunit 3G (PPP1R3G) is one of the regulatory subunits of protein phosphatase 1 (PP1). Current research on PPP1R3G is primarily focused on glycogen synthesis and lipid metabolism. However, the role of *ppp1r3g* in the innate immune response of fish has not been completely elucidated. This study demonstrated that *ppp1r3g* regulates RLR signaling pathway through interactions with Rig-I, Mda5, and Traf6. In parallel, Ppp1r3g inhibits platelet activation by regulating 14-3-3 zeta (14-3-3ζ) and consequently modulating the formation of the 14-3-3ζ-integrin-β3 (Itgb3) complex. *In vivo* analysis revealed that compared with the wild-type controls, *ppp1r3g*-null rare minnow (*Gobiocypris rarus*) and *ppp1r3g*-chimera grass carp (*Ctenopharyngodon idella*) exhibited significantly enhanced resistance to grass carp reovirus (GCRV), with markedly alleviated hemorrhagic symptoms post-infection. Furthermore, knockdown of *ppp1r3g* suppressed GCRV replication and upregulated type I interferon (IFN-1) expression in grass carp kidney (CIK) cells. Additionally, Ppp1r3g binds to the -ESKVFYLKMKGDYYRYL- fragment (EL17) of 14-3-3ζ *via* the Ser36/37 phosphorylation site located on the PP1c domain. This study also demonstrated that 14-3-3ζ interacts with Itgb3 through this fragment, indicating that Ppp1r3g competes with Itgb3 to bind 14-3-3ζ. Furthermore, Ppp1r3g dose-dependently promoted Itgb3 degradation via a proteolytic pathway. These findings indicate that Ppp1r3g negatively regulates innate antiviral immunity after GCRV stimulation and suppresses platelet and coagulation cascade activation by modulating the interaction between 14-3-3ζ and Itgb3 in fish.

## Author summary

Grass carp reovirus (GCRV) infection is associated with high lethality in grass carp and rare minnow. The primary symptom of GCRV is massive hemorrhaging in various tissues (hemorrhagic disease). Therefore, alleviating hemorrhagic symptoms is considered important for improving survival following GCRV infection. This study demonstrated that GCRV promotes *ppp1r3g* induction, suppressing the activation of the fish innate immune system by negatively regulating RLR signaling. Furthermore, Ppp1r3g competes with integrin-β3 (Itgb3) for binding to 14-3-3ζ and promotes Itgb3 degradation in a proteasome-dependent manner, which inhibits the formation of the 14-3-3ζ-Itgb3 complex, suppressing platelet activation. Thus, this study revealed the role of Ppp1r3g in fish innate immunity and elucidated its regulatory mechanism in the coagulation pathway.

## Introduction

The innate immune system is the first line of defense against viral pathogens and microbial invasion [1–3]. Several pattern recognition receptors (PRRs), including Toll-like receptors (TLRs), retinoic acid-inducible gene 1 (RIG-I)-like receptors (RLRs), NOD-like receptors (NLRs), and DNA sensors (cytoplasmic DNA sensors), mediate host defense responses [4,5]. Viral DNA and RNA are detected by the cyclic GMP-AMP synthase-stimulator of interferon genes (cGAS-STING) and RLR pathways, respectively, in infected cells [6,7]. The RLR family members, which primarily include RIG-I, melanoma differentiation-associated gene 5 (MDA5), and laboratory genetics and physiology 2 (LGP2), serve as the main PRRs for recognizing viral RNA and initiating antiviral responses [8,9]. Grass carp reovirus (GCRV), which belongs to the family *Reoviridae*, is a nonenveloped spherical virus with an 11-segmented double-stranded RNA (dsRNA) genome [10,11]. The RLR signaling pathway plays a crucial role in sensing GCRV and activating innate antiviral immune responses. After viral infection, the PRRs RIG-I and MDA5 recognize dsRNA or single-stranded RNA (ssRNA). RIG-I and MDA5 signal through mitochondrial antiviral signaling protein (MAVS) to activate downstream type I IFN signaling [3,4,12,13]. Subsequently, TRAF3, TRAF6, and TBK1 are recruited and activated, leading to the nuclear translocation of IRF3/7 and NF-κB, which ultimately induces type I interferon production [12,14,15]. IFN-I, IFN-stimulated genes (ISGs), and MX proteins induce innate antiviral immune responses [16–18]. Recent studies have demonstrated that the RLR signaling pathway is extensively regulated during GCRV infection. GCRV suppresses host antiviral immunity by targeting MAVS-mediated type I IFN signaling [19], whereas host factors such as DHX40, ATG5, and PKM regulate antiviral responses by modulating key components of the RLR signaling pathway. For example, ATG5 promotes the degradation of RIG-I and MDA5 [20], DHX40 negatively regulates RLR-mediated signaling by targeting RLR-related helicases in host cells [21], and PKM facilitates GCRV infection by inhibiting the RLR–IFN–STAT1 signaling axis [22]. Collectively, these findings indicate that the RLR signaling pathway is subject to complex and dynamic regulation during GCRV infection. However, additional host factors involved in this regulatory network remain to be identified and characterized.

The innate immune system and coagulation system share a common ancestral origin and are interrelated [23–25]. Virus-induced hemorrhage activates the coagulation and complement systems to control bleeding and restrict infection [26,27]. A characteristic feature of GCRV infection in fish is extensive hemorrhage across multiple organs and tissues, which is often accompanied by vascular wall disruption and circulatory necrosis [28,29]. Platelet activation is a critical step in hemostasis and thrombosis, where integrin αIIbβ3 (GPIIb/IIIa) plays a central role [30–33]. Previous studies have demonstrated that the adaptor protein 14-3-3ζ regulates platelet activation through multiple mechanisms, including interactions with GPIbα and modulation of mitochondrial function [34–38]. Integrin αIIbβ3 (GPIIb/IIIa), which is the most abundant glycoprotein on platelets [39], interacts with 14-3-3ζ via its cytoplasmic tail, forming a 14-3-3ζ–c-Src–integrin-β3 complex on the platelet surface to promote platelet activation [30,40]. In addition to serving as a key target regulating outside-in signaling of integrin β3, the adaptor protein 14-3-3ζ binds to GPIbα to modulate platelet activation and thrombosis [35,41,42].

Protein phosphatase 1 (PP1), a widely expressed Ser/Thr phosphatase, comprises catalytic subunits (PP1α, PP1β, and PP1γ) and regulatory subunits (RIPPOs) [43]. Previous studies have reported that PP1 regulates various cellular processes. Additionally, PP1 modulates host antiviral responses [44–46]. PP1α and PP1γ are activated through the dephosphorylation of the CARD domains of MDA5 and RIG-I, inhibiting viral RNA replication and upregulating IFN-I expression [47]. PP1γ interacts with and regulates the activity of the E3 ubiquitin ligase TRAF6 and its substrate (IKKγ), promoting NF-κB-mediated innate signaling responses [48]. Viruses can evade PP1-mediated antiviral signaling by interfering with the interaction between PP1 and RLR pathway components [49]. PPP1R3G is activated via serine/threonine protein kinase (AKT)-mediated phosphorylation and regulates glucose homeostasis and lipid metabolism by modulating glycogen synthase (GS) activity [50,51]. PPP1R3G/PP1γ activates RIPK1 by promoting RIPK1-S357 dephosphorylation to regulate apoptosis and necrosis [52]. Previously, we demonstrated that *ppp1r3g* regulates the innate antiviral immune response to GCRV infection in grass carp by modulating *irf3* [53]. This study focused on the role of *ppp1r3g* in the coagulation pathway after GCRV challenge.

In this study, *ppp1r3g*^−/−^ rare minnow and *ppp1r3g-*chimera grass carp exhibited increased disease resistance and decreased hemorrhage following GCRV infection. The expression levels of immune-related genes (*irf3*, *ifn1*, *mx*, and *isgs*) were upregulated. Experimental data indicated that Ppp1r3g negatively regulates antiviral immune responses by inhibiting IFN-I expression. Furthermore, ppp1r3g modulated 14-3-3ζ to regulate platelet activation, inhibiting downstream coagulation cascade signaling. Thus, *ppp1r3g* deficiency enhances antiviral innate immunity and promotes platelet activation and downstream coagulation signaling.

## Results

### Grass carp *ppp1r3g* negatively regulates antiviral innate immunity *in vitro* through RLR signaling

**Fig 1:**
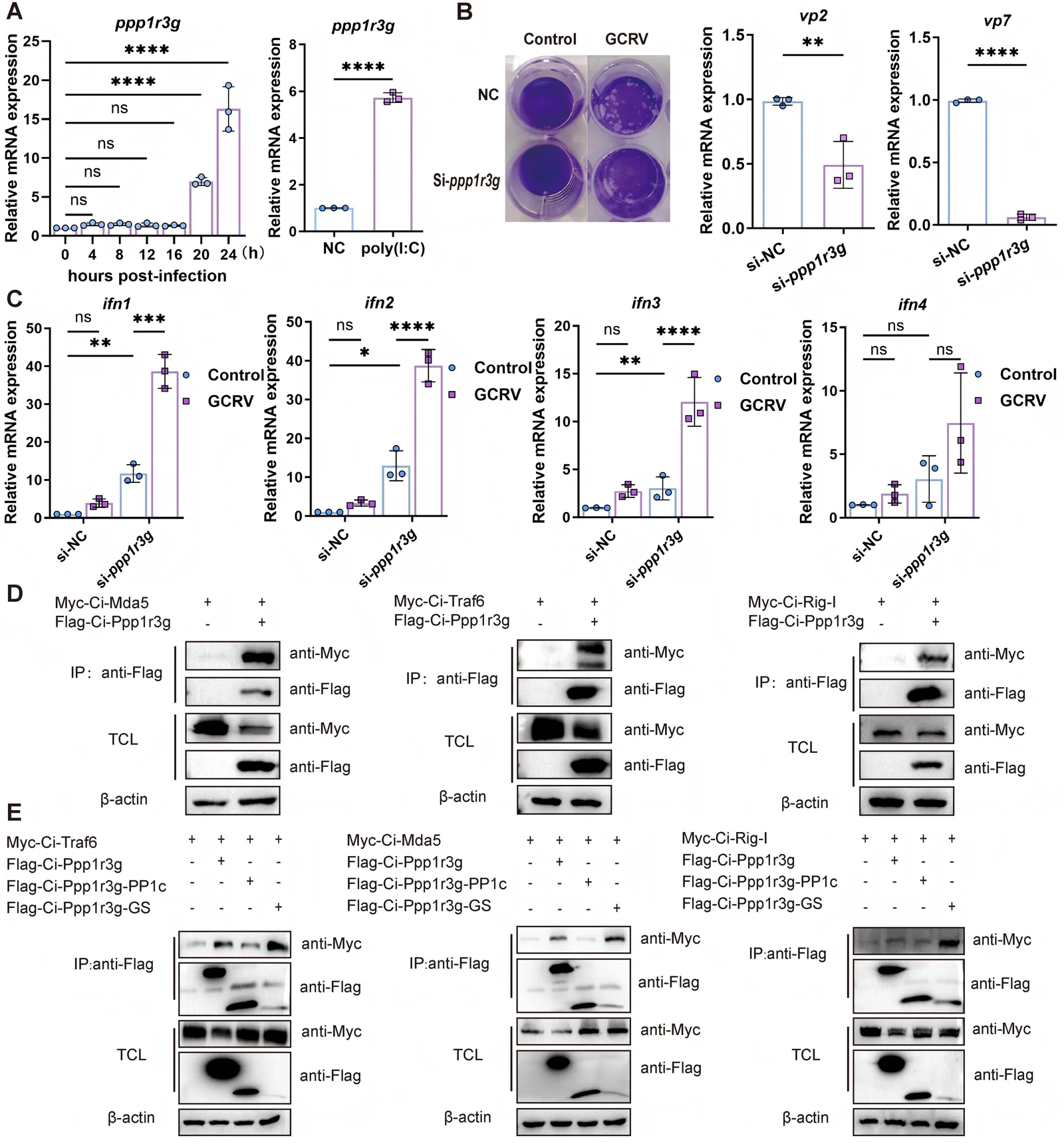
Grass carp Ppp1r3g negatively regulates antiviral innate immunity in cells through RLR signaling. **(A)** GCRV infection and poly(I:C) stimulation significantly increased the expression of *ppp1r3g*. CIK cells were infected with GCRV (strain HZ08; MOI = 0.2) or treated with poly(I) for the indicated times. The mRNA levels of *ppp1r3g* were determined by qRT-PCR. **(B-C)** CIK cells were transfected with si-NC or si-Ci*-ppp1r3g* and subsequently infected with GCRV. The mRNA levels of *vp2*, *vp7*, *ifn1*, *ifn2*, *ifn3*, and *ifn4* were analyzed by qRT-PCR. Cytopathic effects (CPEs) were evaluated by crystal violet staining. **(D)** Ci-Ppp1r3g interacted with Ci-Rig-I, Ci-Mda5, and Ci-Traf6. HEK293T cells were transfected with the indicated plasmids. At 24 h post-transfection, the lysates were immunoprecipitated using anti-Flag magnetic beads and subjected to immunoblotting analysis with anti-Flag and anti-Myc antibodies. **(E)** Interaction of Ci-Ppp1r3g truncation mutants with Ci-Rig-I, Ci-Mda5, and Ci-Traf6. HEK293T cells were co-transfected with the indicated plasmids. At 24 h post-transfection, the cell lysates were immunoprecipitated with anti-Flag magnetic beads and analyzed by immunoblotting using anti-Flag and anti-Myc antibodies, respectively. All the data represent the mean of three independent replicates. The error bars indicate the standard deviation (n = 3). ns, not significant; ^*^P< 0.05, ^**^P < 0.01, ^***^P < 0.001, ^****^P < 0.0001.

To investigate the functional mechanism of *ppp1r3g* from grass carp (Ci-*ppp1r3g*) during GCRV infection, CIK cells were stimulated with poly(I:C) (a mimic of RNA virus) [54] and GCRV for 0–24 h. Quantitative real-time polymerase chain reaction (qRT-PCR) analysis revealed that stimulation with poly(I:C) and GCRV significantly upregulated Ci*-ppp1r3g* mRNA expression levels (Fig 1A). Furthermore, knockdown of Ci-*ppp1r3g* suppressed the mRNA expression of the viral genes *vp2* and *vp7* and the cytopathogenic effect (CPE). Compared with those in the control group, the viral titers in the Ci*-ppp1r3g* knockdown group were significantly lower (Fig 1B). qRT-PCR analysis revealed that the mRNA expression levels of type I interferon (*ifn1*, *ifn2*, and *ifn3*) family members were significantly upregulated in Ci-*ppp1r3g* knockdown CIK cells (Fig 1C). Activation of the RLR antiviral signaling pathway is essential for viral recognition and induction of the type I IFN response. Co-immunoprecipitation (Co-IP) assays demonstrated that Ci-Ppp1r3g interacted with Ci-Rig-I, Ci-Mda5, and Ci-Traf6 in HEK293T cells (Fig 1D). In contrast, no interactions were detected between Ci-Ppp1r3g and other key RLR signaling components, including Ci-Lgp2, Ci-Mavs, Ci-Traf3, and Ci-Tbk1 (S1A-D Fig). To further validate these interactions in a teleost cell model, Co-IP assays were also performed in GCO cells. Consistent with the results obtained in HEK293T cells, Ci-Ppp1r3g also interacted with Ci-Rig-I, Ci-Mda5, and Ci-Traf6 in GCO cells, further confirming the conserved interaction pattern across different cellular systems (S1E-G Fig). To further elucidate the regions where Ppp1r3g interacts with Mda5, Traf6, and Rig-I, two truncated constructs corresponding to the PP1c domain (Ci-Ppp1r3g-PP1c) and the GS domain (Ci-Ppp1r3g-GS) were generated. Co-IP assays revealed that Mda5, Traf6, and Rig-I co-precipitated with the GS-domain fragment but not with the PP1c-domain fragment. These findings demonstrate that the GS domain of Ppp1r3g is responsible for mediating its interaction with Mda5, Traf6, and Rig-I (Fig 1E). These findings indicate that Ci-Ppp1r3g is selectively associated with specific components of the RLR signaling pathway and participates in the regulation of GCRV-induced innate antiviral responses.

### *ppp1r3g*^−/−^ rare minnow and grass carp *ppp1r3g*-chimeras exhibit enhanced resistance to GCRV

**Fig 2:**
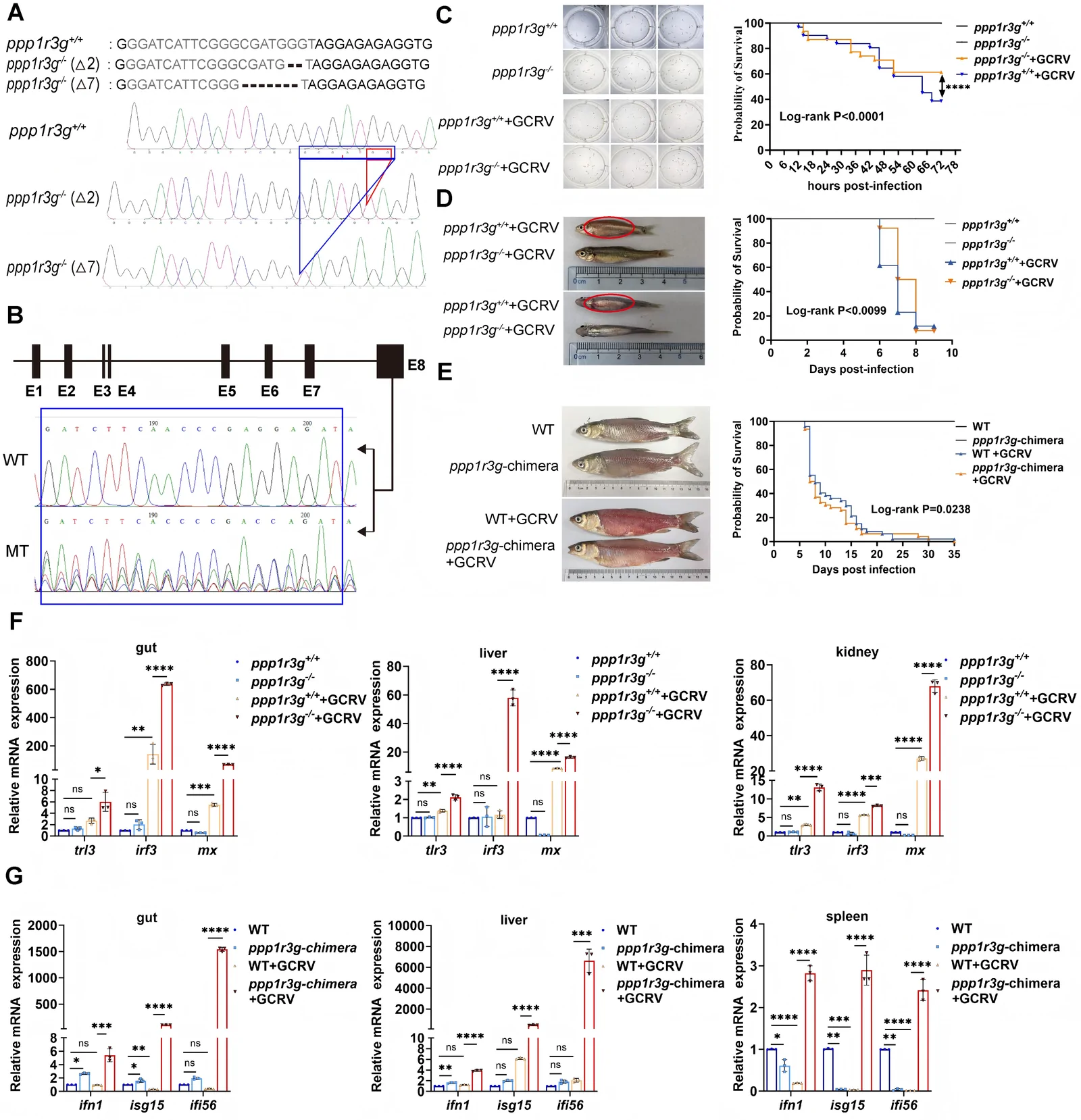
*ppp1r3g*^−/−^ rare minnow and *ppp1r3g*-chimera grass carp exhibit enhanced resistance to GCRV. **(A)** Schematic diagram of the *ppp1r3g* deletion target site and sequence information in the rare minnow. *ppp1r3g*^△2^ involves the deletion of two bases (GG) in *ppp1r3g*, and *ppp1r3g*^△7^ involves the deletion of seven bases (CGATGGG) in *ppp1r3g*. **(B)** Schematic diagram of the target site and sequence information for the grass carp *ppp1r3g*-chimera. **(C)** Representative images and mortality curves of *ppp1r3g*-deficient rare minnow larvae (*ppp1r3g*^−/−^; n = 30; 3 dpf) and their wild-type siblings (*ppp1r3g*^+/+^; n = 30, 3 dpf) following GCRV infection. Larvae were exposed to GCRV (approximately 2.97 × 10^3^ RNA copies/μL), and survival was monitored for 72 h. **(D)** Representative images and mortality curves of *ppp1r3g*-deficient rare minnow adults (*ppp1r3g*^−/−^; n = 26; 6 mpf) and their wild-type siblings (*ppp1r3g*^+/+^; n = 26, 6 mpf) following GCRV infection. The fish were immersed in GCRV solution (approximately 2.97 × 10^3^ RNA copies/μL) for 20 min. Mortality was recorded daily for both groups. Red circles indicate hemorrhagic lesions. **(E)** Representative images and mortality curves of *ppp1r3g*-chimera grass carp juveniles (n = 48; 6 mpf) and their wild-type siblings (n = 48; 6 mpf) following GCRV infection. The fish were immersed in GCRV solution for 20 min. Mortality was recorded daily for both groups. **(F)** The mRNA levels of key antiviral genes (*tlr3*, *irf3*, and *mx)* in *ppp1r3g*^−/−^ rare minnow were upregulated compared with those in *ppp1r3g*^+/+^ individuals after GCRV infection. **(G)** The mRNA levels of key antiviral genes (*ifn1*, *isg15*, and *ifi56)* in *ppp1r3g*-chimera grass carp were significantly upregulated compared with those in WT controls after GCRV infection. The expression levels were normalized using *β*-actin as an internal reference. The error bars indicate the standard deviation (n = 3). ns, not significant; ^*^P< 0.05, ^**^P < 0.01, ^***^P < 0.001, ^****^P < 0.0001; compared with the control group. All the data represent the mean of three independent replicates.

In this study, *ppp1r3g*-deficient rare minnow (*ppp1r3g*^−/−^) and *ppp1r3g*-chimera grass carp (*ppp1r3g*-chimera) were generated using clustered regularly interspaced short palindromic repeats-associated protein 9 (CRISPR-Cas9) technology (Fig 2A). qRT-PCR analysis confirmed that the mRNA level of *ppp1r3g* was efficiently downregulated in both the *ppp1r3g*^−/−^ rare minnow and the *ppp1r3g*-chimera grass carp (S2A-B Fig). The target site and sequence information of the grass carp *ppp1r3g*-chimera are shown in Fig 2B. The growth rate and reproductive capacity of *ppp1r3g* ^−/−^ rare minnow and *ppp1r3g*-chimera grass carp were not significantly affected under normal conditions. Triplicate groups of *ppp1r3g*^−/−^ rare minnow (*n* = 30) larvae were analyzed (day 3 post-fertilization), whereas *ppp1r3g*^+/+^ (wild-type [WT]) rare minnow larvae served as the control. The survival curves were compared using log-rank analysis (Fig 2C). Compared with those in the control group, the resistance to GCRV and the survival rates were significantly greater in *ppp1r3g*^−/−^ rare minnow larvae. Next, adult rare minnow (6 months post-fertilization, 6 [mpf]) of both the *ppp1r3g*^−/−^ and *ppp1r3g*^+/+^ strains were infected with GCRV *via* immersion challenge. Changes in the body surface and overall condition were monitored daily. Compared with *ppp1r3g*^+/+^ minnows, *ppp1r3g*^−/−^ rare minnows exhibited significantly decreased hemorrhaging in the abdomen and lower jaw. The body surface of *ppp1r3g*^−/−^ rare minnow occasionally exhibited no hemorrhaging. Additionally, the number of rare minnow deaths on different days post-GCRV infection was recorded, and survival curves were constructed (Fig 2D). Compared with those in *ppp1r3g*^*+/+*^ rare minnows, GCRV infection-induced hemorrhagic symptoms in *ppp1r3g*^−/−^ rare minnow were significantly alleviated, with a delayed onset. Similarly, 6 mpf *ppp1r3g*-chimeras of grass carp (F_0_ generation *ppp1r3g*-knockout larvae with detectable effects) (*n* = 48) were selected. WT grass carp were selected as a control. The *ppp1r3g*-chimera group and the control group were infected with GCRV via immersion challenge. The external characteristics were monitored daily after infection. The fish were dissected after death, and survival curves were plotted (Fig 2E). To investigate the impact of *ppp1r3g* deficiency on the IFN response, the mRNA expression levels of three antiviral genes (*irf3*, *mx*, and *tlr3*) were compared between *ppp1r3g*^−/−^ and *ppp1r3g*^+/+^ WT adult rare minnow after GCRV infection. qRT-PCR analysis revealed that compared with those in the control groups, the levels of GCRV-induced antiviral genes in *ppp1r3g*^−/−^ adult rare minnow were markedly upregulated (Fig 2F). Additionally, the mRNA expression levels of antiviral genes (*ifn1*, *isg15*, and *ifi56*) in GCRV-infected *ppp1r3g*^−/−^-chimera grass carp larvae were significantly greater than those in *ppp1r3g*^+/+^ larvae (Fig 2G). These findings indicate that the deficiency of *ppp1r3g* expression suppresses GCRV infection-induced hemorrhage, activates innate immune pathways, and promotes downstream IFN-1 production.

### GCRV-induced hemorrhage is alleviated in *ppp1r3g*^−/−^ rare minnow and *ppp1r3g*-chimeras grass carp

**Fig 3:**
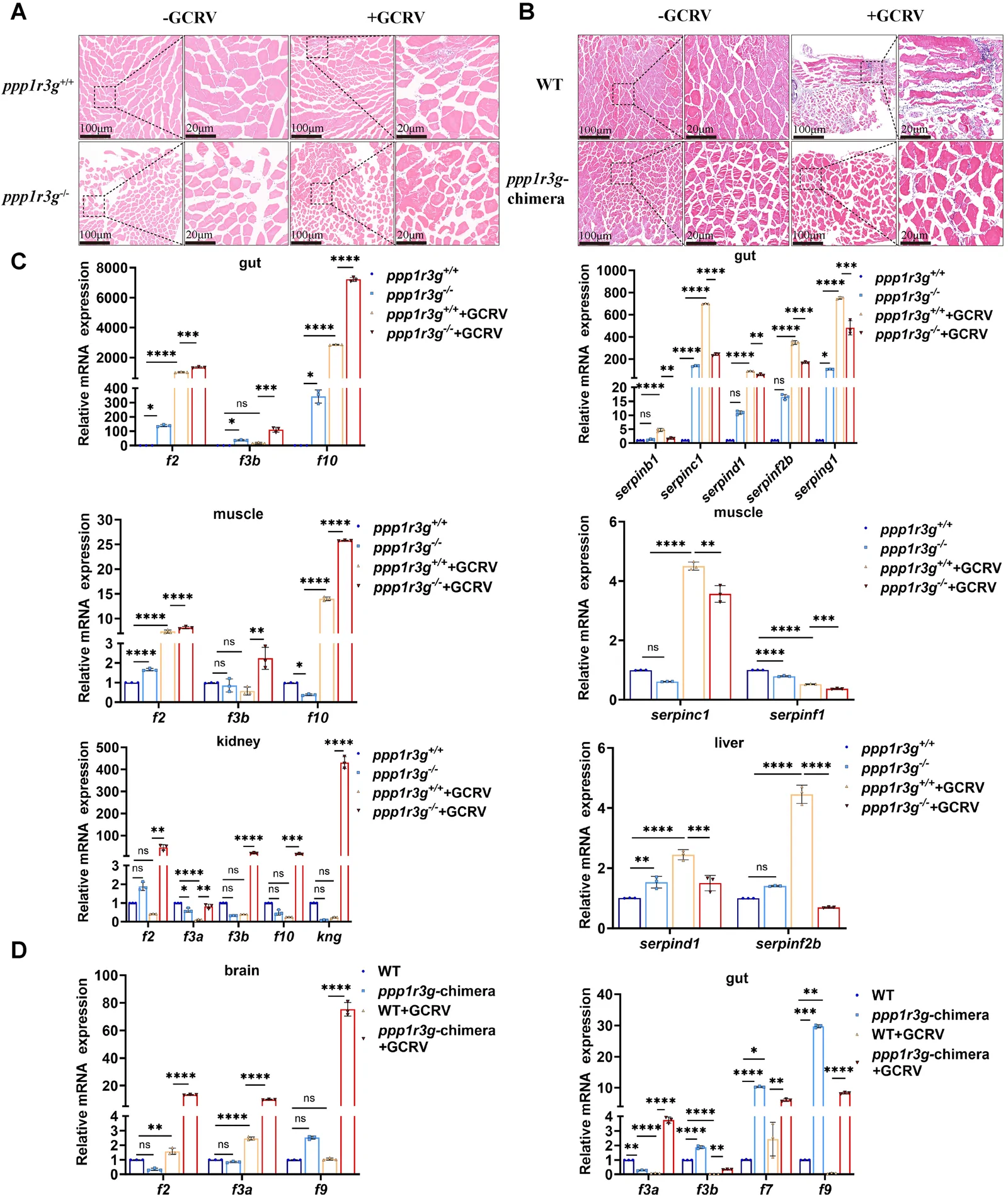
*ppp1r3g*^−/−^ rare minnow and *ppp1r3g*-chimeras grass carp exhibited significantly alleviated hemorrhagic symptoms compared with wild-type (WT) individuals after grass carp reovirus (GCRV) infection. **(A)** Hematoxylin and eosin (H&E)-stained sections of GCRV-infected and uninfected *ppp1r3g*^−/−^ and *ppp1r3g*^+/+^ rare minnow. *ppp1r3g*^+/+^ individuals exhibited exacerbated hemorrhagic symptoms compared with *ppp1r3g*^−/−^ individuals (scale bar = 100 μm). **(B)** H&E-stained sections of *ppp1r3g*-chimeras and WT grass carp before and after GCRV infection. *ppp1r3g*-chimera individuals exhibited alleviated hemorrhagic symptoms compared with WT controls (scale bar = 100 μm). **(C)** qRT-PCR analysis of coagulation factor genes (*kng, f2*, *f3a*, *f3b*, and *f10)* and anticoagulant factors (*serpinb1, serpinc1, serpind1, serpinf1, serpinf2b*, and *serping1*) in the tissues of *ppp1r3g*^+/+^ and *ppp1r3g*^−/−^ rare minnow before and after GCRV infection. **(D)** qRT-PCR analysis of coagulation-related genes (*f2*, *f3a*, *f3b*, *f7*, and *f9*) in the brain and gut tissues of *ppp1r3g*^+/+^ and *ppp1r3g*-chimeras grass carp before and after GCRV infection. The error bars indicate the standard deviation (n = 3). (ns, not significant; ^*^P < 0.05, ^**^P < 0.01, ^***^P< 0.001, ^****^P< 0.0001). All the data are presented as the mean of three independent replicates.

To examine hemorrhages in the muscle tissues of rare minnow and grass carp, the muscle tissues from uninfected and infected fish were subjected to histopathological analysis using hematoxylin and eosin staining. GCRV infection promoted the formation of punctate hemorrhages in the muscles of diseased fish. Compared with the WT controls, both *ppp1r3g*^−/−^ rare minnow and *ppp1r3g*-chimera grass carp larvae exhibited significantly decreased muscle hemorrhage (Fig 3A–B). Next, the effect of *ppp1r3g* deficiency on hemorrhagic symptoms was examined in rare minnow and grass carp infected with GCRV. The mRNA levels of procoagulant factors and serpin family members involved in anticoagulation processes were determined using qRT-PCR analysis. Compared with those in WT controls, the mRNA levels of procoagulant factor genes (*kng*, *f2*, *f3b*, *f3a*, and *f10*) were upregulated in *ppp1r3g*^−/−^ rare minnow. In contrast, the tissue levels of anticoagulant factors (*serpinb1*, *serpinc1*, *serpind1*, *serpinf2b*, *serping1* and *serpinf1*) in *ppp1r3g*^−/−^ rare minnow were significantly lower than those in WT controls (Fig 3C). The brain and gut levels of procoagulants (*f2*, *f3a*, *f7*, and *f9*) in *ppp1r3g*-chimera grass carp were upregulated compared with those in WT controls (Fig 3D). These findings indicate that Ppp1r3g deficiency activates the coagulation pathway, suppresses anticoagulant factors, and significantly alleviates GCRV infection-induced hemorrhage.

### Interaction between Ppp1r3g and 14-3-3ζ

**Fig 4:**
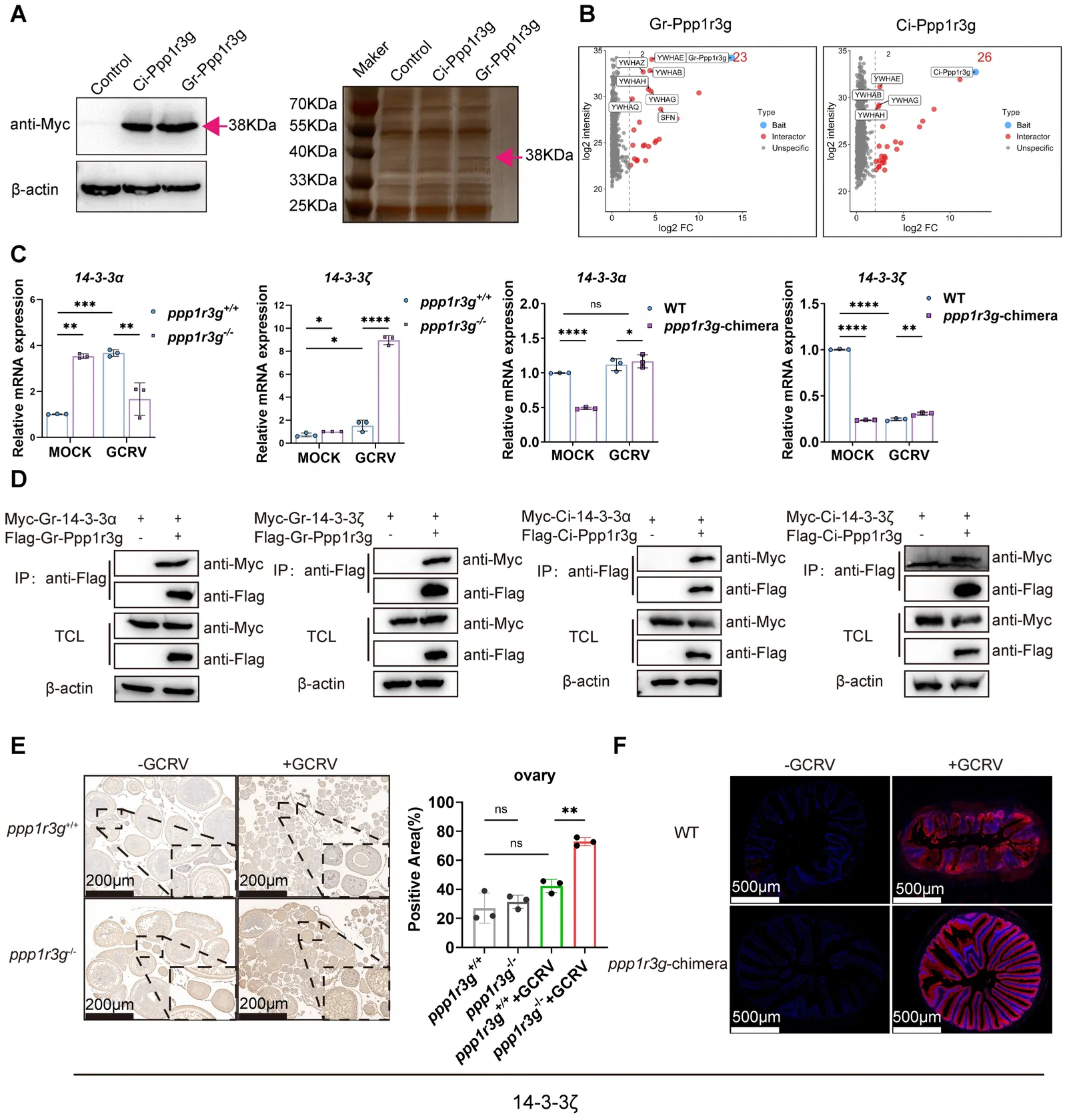
Interaction between Ppp1r3g and 14-3-3ζ. **(A)** Assessment of protein expression and silver staining quality in cell lines stably transfected with Ci-Ppp1r3g and Gr-Ppp1r3g. *β*-actin served as the internal control. The arrows indicate the band positions corresponding to Ci-Ppp1r3g and Gr-Ppp1r3g (≈38 kDa). The silver staining signals were consistent with the western blotting results. **(B)** Volcano plots showing proteins enriched in immunoprecipitation-mass spectrometry experiments for Ci-Ppp1r3g and Gr-Ppp1r3g. **(C)** Quantitative real-time polymerase chain reaction (qRT-PCR) analysis of *14-3-3α* and *14-3-3ζ* expression in the liver tissues of wild-type (WT) and *ppp1r3g*^−/−^ rare minnow, as well as WT and *ppp1r3g*-chimera grass carp following GCRV infection. **(D)** Interaction of Ppp1r3g with 14-3-3ζ and 14-3-3α in grass carp and rare minnow. HEK293T cells were transfected with the indicated plasmids. At 24 h post-transfection, the lysates were immunoprecipitated using anti-Flag magnetic beads and subjected to immunoblotting analysis with anti-Flag and anti-Myc antibodies. **(E)** Localization and expression of 14-3-3ζ in the ovarian tissues of WT and *ppp1r3g*^−/−^ rare minnow before and after GCRV infection. The positive signals of 14-3-3ζ appear brownish-yellow (scale bar = 500 μm). Quantitative analysis of 14-3-3ζ-positive areas. **(F)** Localization and expression of 14-3-3ζ in the gut tissues of WT grass carp and *ppp1r3g*-chimera before and after GCRV infection. The red signal represents 14-3-3ζ, whereas the blue signal indicates cell nuclei (scale bar = 500 μm). All the data are presented as the mean of three independent replicates. The error bars indicate the standard deviation. ns, not significant; ^*^P< 0.05, ^**^P< 0.01, ^***^P< 0.001, ^****^P< 0.0001. Ci: *Ctenopharyngodon idella*; Gr: *Gobiocypris rarus*.

Stable K562 cell lines expressing Ci-Ppp1r3g and rare minnow Ppp1r3g (Gr-Ppp1r3g) were generated by transfection with lentiviral plasmids to investigate the mechanism by which Ppp1r3g regulates the coagulation pathway. Western blotting and protein silver staining confirmed the physiological expression of the Ci-Ppp1r3g and Gr-Ppp1r3g proteins (Fig 4A). Immunoprecipitation-mass spectrometry (IP-MS) analysis (S1 Table) revealed multiple 14-3-3 family members that simultaneously interacted with both Ci-Ppp1r3g and Gr-Ppp1r3g (Fig 4B). qRT-PCR analysis revealed that, following GCRV infection, the expression levels of 14-3-3ζ in *ppp1r3g*^−/−^ rare minnow and *ppp1r3g*-chimera grass carp were significantly greater than those in WT controls (Fig 4C). Co-IP demonstrated that 14-3-3ζ and 14-3-3α can interact with Ppp1r3g from both species *in vitro* (Fig 4D). Immunohistochemical (IHC) analysis revealed that the 14-3-3ζ signal intensity in the ovarian tissue of *ppp1r3g*^−/−^ rare minnow was greater than that in the ovarian tissue of WT rare minnow after GCRV infection (Fig 4E). Immunofluorescence (IF) analysis revealed that the 14-3-3ζ signal intensity in the gut tissues of *ppp1r3g*-chimera grass carp was significantly greater than that in the gut tissues of WT grass carp (Fig 4F). These results indicate that Ppp1r3g negatively regulates the expression of the 14-3-3ζ protein, suggesting that Ppp1r3g may regulate coagulation-related responses through the 14-3-3ζ signaling pathway.

### Site for binding of Ppp1r3g with 14-3-3ζ

**Fig 5:**
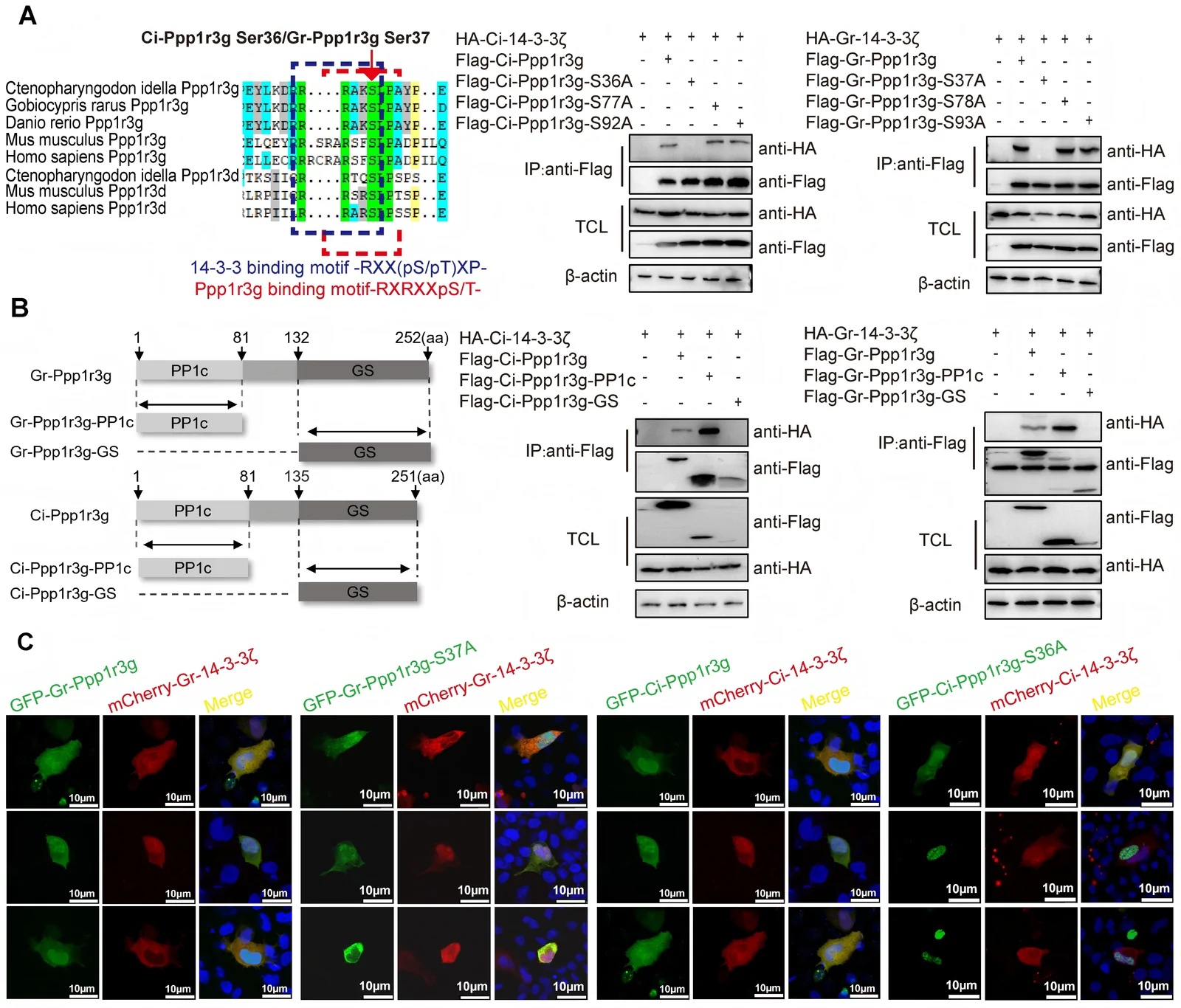
Protein-binding motifs of Ppp1r3g and 14-3-3ζ. **(A)** Interaction of Ppp1r3g with 14-3-3ζ via Ser36/37 in grass carp and rare minnow. HEK293T cells were transfected with the indicated plasmids. At 24 h post-transfection, total cell lysates were immunoprecipitated with anti-Flag magnetic beads, and the immunoprecipitates were analyzed by immunoblotting with anti-HA and anti-Flag antibodies. **(B)** The PP1c domain of Ppp1r3g is required for interaction with 14-3-3ζ. HEK293T cells were transfected with the indicated plasmids. At 24 h post-transfection, the lysates were immunoprecipitated with anti-Flag magnetic beads, followed by immunoblotting with anti-HA and anti-Flag antibodies. **(C)** Subcellular colocalization of Ppp1r3g and 14-3-3ζ and the effect of the Ser36/37 mutation in grass carp and rare minnow. GCO cells were transfected with the indicated plasmids. At 24 h post-transfection, the cells were fixed and analyzed by confocal microscopy. Red, green, and blue signals indicate 14-3-3ζ, Ppp1r3g, and nuclei, respectively (scale bar = 10 μm). Ci: *Ctenopharyngodon idella*; Gr: *Gobiocypris rarus*.

14-3-3 proteins commonly mediate molecular interactions by recognizing and binding to phosphorylated serine/threonine (Ser/Thr) residues present on target proteins [55,56]. To identify the binding site between Ppp1r3g and 14-3-3ζ, non-phosphorylatable mutants were generated by substituting serine residues (Ser36/37, Ser77/78, and Ser92/93) with alanine (S36/37A, S77/78A, and S92/93A). Co-IP analysis revealed that the introduction of the Ser36/37A mutation, but not that of Ser77/78A and Ser92/93A, resulted in the loss of interaction between Ppp1r3g and 14-3-3ζ (Fig 5A). Therefore, Ppp1r3g binds to the 14-3-3ζ protein at the Ser36/37 phosphorylation site. To determine which domain of Ppp1r3g mediates its interaction with 14-3-3ζ, previously generated Ppp1r3g truncation constructs were examined by Co-IP assays. The results revealed that 14-3-3ζ interacted with the PP1c domain fragment but not with the GS domain fragment in both species. These results indicate that 14-3-3ζ specifically interacts with the PP1c domain of Ppp1r3g (Fig 5B). Next, the subcellular localization of 14-3-3ζ with Ppp1r3g and Ppp1r3g-ser36/37A was examined. mCherry -tagged 14-3-3ζ (red fluorescence) and green fluorescent protein (GFP)-tagged Ppp1r3g (green fluorescence) were expressed in both the nucleus and the cytoplasm of grass carp ovary (GCO) cells with both proteins colocalizing in the cytoplasm (yellow fluorescence). The introduction of the Ser36/37 site mutation resulted in the significant reduction or complete absence of the cytoplasmic colocalization (yellow fluorescence) of mCherry-tagged 14-3-3ζ (red fluorescence) and GFP-tagged Ppp1r3g-ser36/37A (green fluorescence) in both species. (Fig 5C). These results indicate that 14-3-3ζ binds to the PP1c domain of Ppp1r3g via Ser36/37. Furthermore, 14-3-3ζ colocalized with Ppp1r3g in the cytoplasm.

### Interaction between 14-3-3ζ and Itgb3

**Fig 6:**
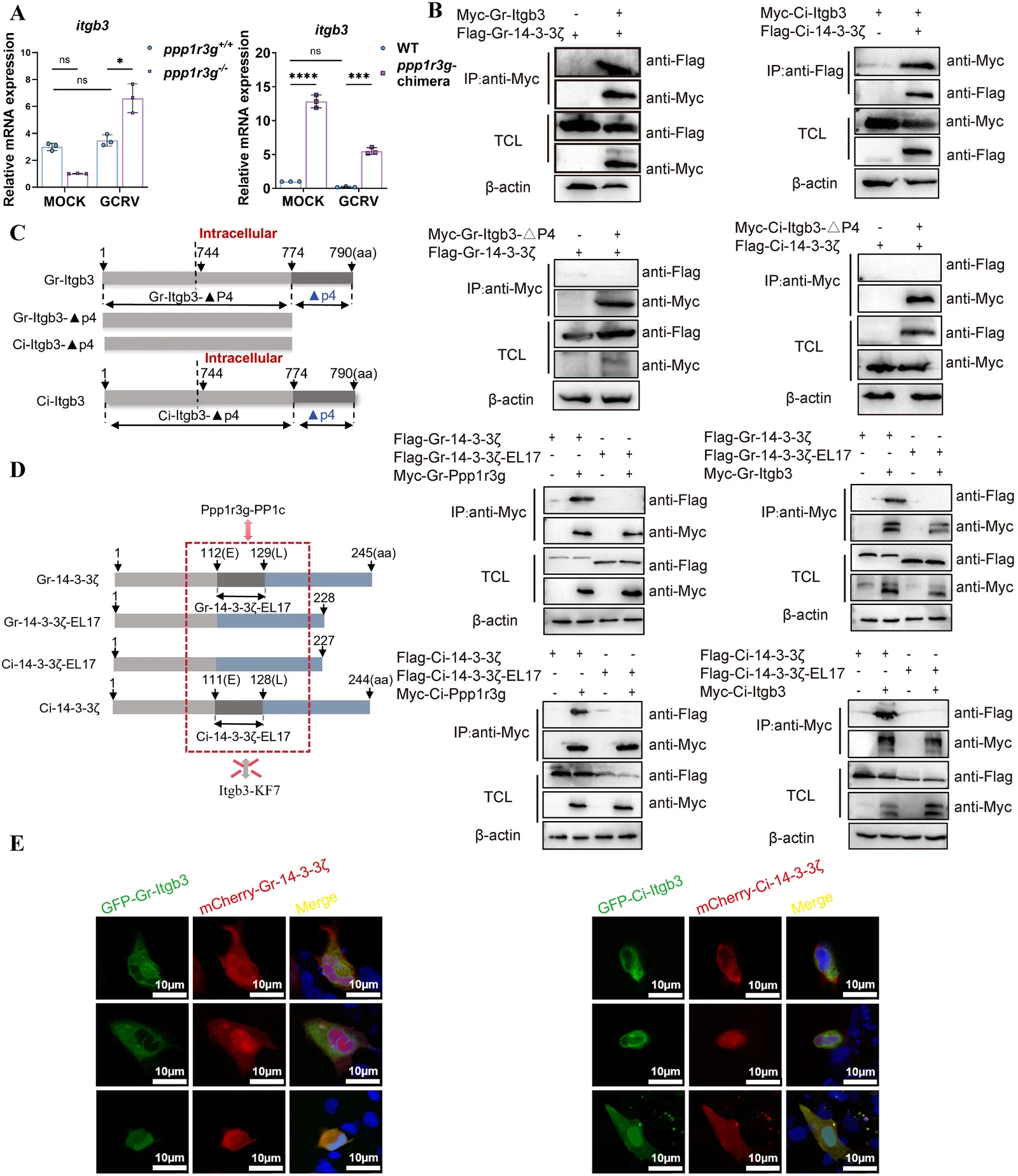
Interaction between 14-3-3ζ and Itgb3. **(A)** Quantitative real-time polymerase chain reaction (qRT-PCR) analysis of Ci-*itgb3* and Gr-*itgb3* expression in the intestinal tissues of grass carp and rare minnow. The error bars indicate the standard deviation (n = 3). ns, not significant; ^*^P< 0.05, ^**^P< 0.01, ^****^P< 0.0001. **(B)** Interaction between Itgb3 and 14-3-3ζ in grass carp and rare minnow. HEK293T cells were transfected with the indicated plasmids. At 24 h post-transfection, the cell lysates were immunoprecipitated using anti-Flag or anti-Myc magnetic beads. The immunoprecipitated samples were subjected to immunoblotting with anti-Flag and anti-Myc antibodies. **(C)** Interaction between Itgb3-ΔP4 and 14-3-3ζ in grass carp and rare minnow. HEK293T cells were transfected with the indicated plasmids. At 24 h post-transfection, the cell lysates were immunoprecipitated with anti-Flag or anti-Myc magnetic beads, and the immunoprecipitates were analyzed by immunoblotting with the corresponding antibodies. **(D)** Interaction of Ppp1r3g and Itgb3 with 14-3-3ζ via the EL17 peptide in grass carp and rare minnow. HEK293T cells were transfected with the indicated plasmids. At 24 h post-transfection, the cell lysates were immunoprecipitated with anti-Flag or anti-Myc magnetic beads, and the immunoprecipitates were analyzed by immunoblotting with anti-Flag and anti-Myc antibodies. **(E)** Subcellular localization of Itgb3 and 14-3-3ζ in grass carp and rare minnow. HEK293T cells were transfected with the indicated plasmids. At 24 h post-transfection, the cells were fixed and analyzed by confocal microscopy. Red, green, and blue signals indicate 14-3-3ζ, Itgb3, and nuclei, respectively (scale bar = 10 μm). All the data are presented as the mean of three independent replicates. Ci: *Ctenopharyngodon idella*; Gr: *Gobiocypris rarus*.

The transcriptomic data were analyzed to identify key downstream genes involved in coagulation to further investigate the molecular mechanism through which Ppp1r3g targets 14-3-3ζ and regulates the coagulation pathway. qRT-PCR analysis revealed that the mRNA levels of *itgb3* in the gut tissues in *ppp1r3g*^−/−^ rare minnow and *ppp1r3g*-chimera grass carp were significantly greater than those in their WT counterparts after GCRV infection (Fig 6A). Additionally, Co-IP analysis of HEK293T cells co-expressing plasmids from both species confirmed the interaction between 14-3-3ζ and Itgb3 (Fig 6B). Furthermore, Co-IP analysis revealed that GCRV infection weakened the interaction between 14-3-3ζ and Itgb3 in GCO cells. Notably, the interaction between rare minnow 14-3-3ζ and Itgb3 was no longer detectable after GCRV infection, whereas the interaction between the grass carp proteins was substantially reduced (S3A-B Fig). These results indicate that GCRV infection disrupts the association between 14-3-3ζ and Itgb3. A previous study revealed that 14-3-3ζ interacts with the -ESKVFYLKMKGDYYRYL- fragment (EL17) and the cytoplasmic tail of Itgb3 *via* the -KEATSTF- fragment (KF7) [30]. In this study, the cytoplasmic tail ΔP4 of Itgb3 from both species was truncated to obtain the recombinant plasmids Flag-tagged Ci-Itgb3-ΔP4 and Myc-tagged Gr-Itgb3-ΔP4 (Fig 6C). These recombinant plasmids were overexpressed in HEK293T cells, which were then subjected to Co-IP analysis. The interaction between 14-3-3ζ and Itgb3 was abolished (Fig 6C). Additionally, the EL17 peptide segment of 14-3-3ζ was truncated to construct the recombinant Myc-tagged Ci-14-3-3ζ-EL17 and Flag-tagged Gr-14-3-3ζ-EL17 plasmids (Fig 6D). These recombinant plasmids were transfected into HEK293T cells, which were then subjected to Co-IP analysis. The truncation of the EL17 segment of 14-3-3ζ inhibited its interactions with both Itgb3 and Ppp1r3g (Fig 6D). These findings suggest that Ppp1r3g and Itgb3 bind to 14-3-3ζ at the same site, indicating a competitive binding relationship. Subcellular localization analysis revealed that green fluorescent protein (GFP)-tagged Itgb3 (green fluorescence) was expressed on the cell membrane, whereas red fluorescent protein (mCherry)-tagged 14-3-3ζ (red fluorescence) was detected in both the nucleus and cytoplasm. In both species, these two proteins colocalized in the cytoplasm (yellow fluorescence) (Fig 6E).

### Ppp1r3g promotes the degradation of Itgb3

**Fig 7:**
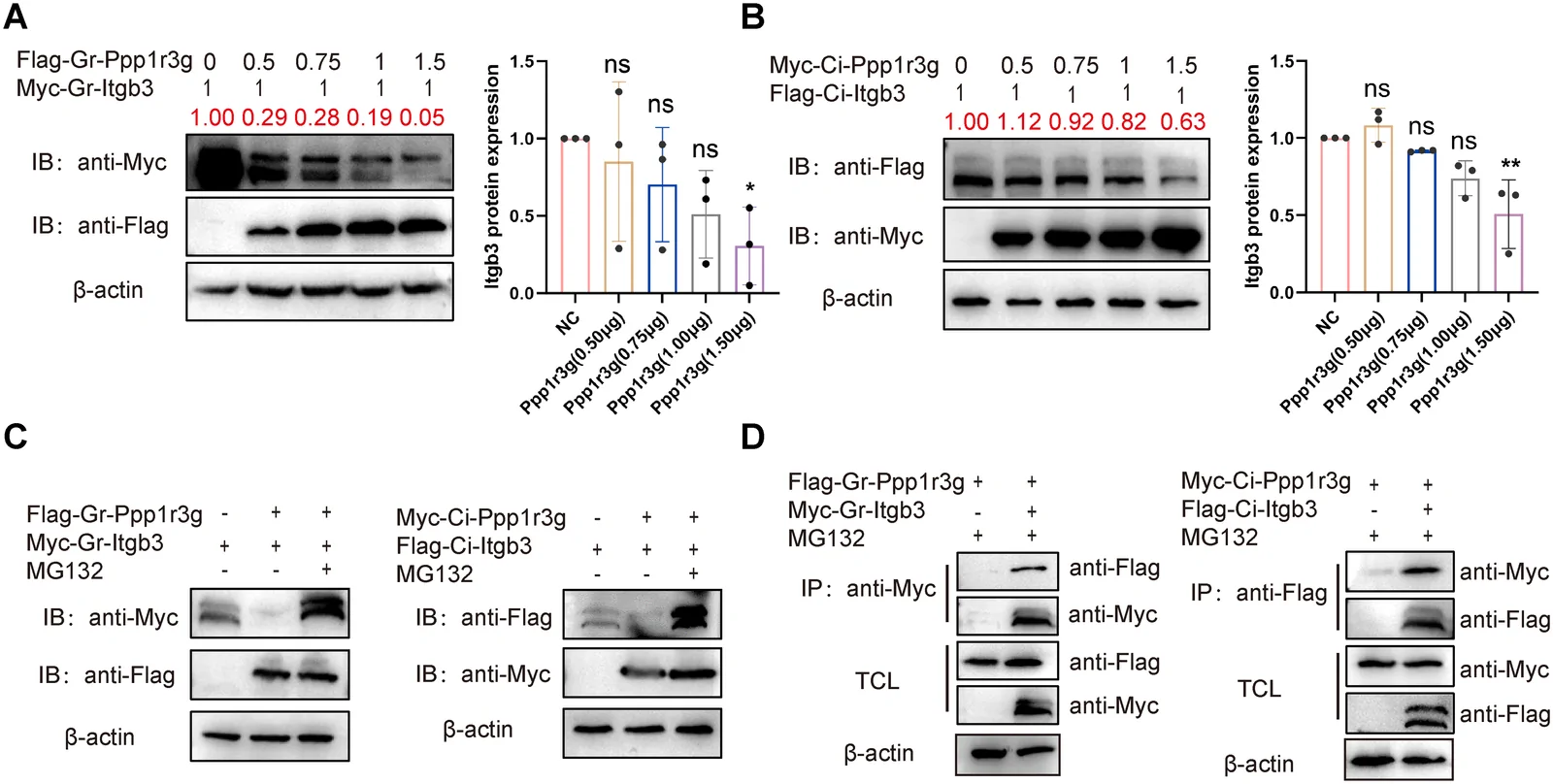
Ppp1r3g promotes the degradation of Itgb3. **(A-B)** Dose-dependent effect of Ppp1r3g on Itgb3 degradation. HEK293T cells were transfected with the indicated plasmids. Total cell lysates were collected at 24 h post-transfection and analyzed by immunoblotting. **(C)** Ppp1r3g promotes proteasome-dependent degradation of Itgb3. HEK293T cells were transfected with the indicated plasmids. At 16 h post-transfection, the cells were treated with MG132 (20 μM) or the same volume of dimethyl sulfoxide for 8 h. Total cell lysates were collected and subjected to immunoblotting using the indicated antibodies. **(D)** Ppp1r3g interacts with Itgb3 upon proteasome inhibition. HEK293T cells were transfected with the indicated plasmids. At 16 h post-transfection, cells were treated with MG132 (20 μM) for 8 h. Total cell lysates were immunoprecipitated with anti-Myc magnetic beads. The total cell lysates and immunoprecipitated samples were probed with anti-Flag and anti-Myc antibodies, respectively.

To investigate the effect of Ppp1r3g on the stability of 14-3-3ζ and Itgb3, recombinant plasmids encoding Ppp1r3g and Itgb3 from grass carp or rare minnow were transiently expressed in HEK293T cells. Quantitative analysis of three independent Western blot experiments demonstrated that Ppp1r3g induced dose-dependent degradation of Itgb3 (Fig 7A-B). Similar results were observed in GCO cells, indicating that Ppp1r3g-mediated Itgb3 degradation also occurs in fish cells (S4A-B Fig). To further determine whether Ppp1r3g also affects the stability of 14-3-3ζ, Ppp1r3g, 14-3-3ζ, and Itgb3 were co-expressed in HEK293T cells. Ppp1r3g promoted dose-dependent degradation of Itgb3, whereas the protein level of 14-3-3ζ remained unchanged (S5A-B Fig). To elucidate the pathway involved in Ppp1r3g-mediated Itgb3 degradation, cells were treated with MG132, 3-MA, or NH_4_Cl. MG132 markedly restored Itgb3 protein levels (Fig 7C). In contrast, NH_4_Cl inhibited Ppp1r3g-mediated Itgb3 degradation only in rare minnow, whereas 3-MA had no detectable effect on either species (S6A-D Fig). However, ubiquitination of Itgb3 was not detected under these conditions. (S7A-B Fig). Given that MG132 restored Itgb3 protein levels, whereas ubiquitination of Itgb3 was not detected, we next examined whether Ppp1r3g directly associates with Itgb3. Co-IP revealed an interaction between Ppp1r3g and Itgb3 in the presence of the proteasome inhibitor MG132 (Fig 7D). To further evaluate the functional relevance of *itgb3* in *ppp1r3g*-mediated antiviral regulation, we examined the expression of IFNs, the interferon-stimulated gene *isg15* and *rig-i* following *itgb3* modulation after GCRV infection (S8A-F Fig). *itgb3* overexpression altered the expression of *ifn3*, *isg15*, *ifn-*γ*2*, and *rig-i*, and degradation of *itgb3* by Ppp1r3g partially reversed these effects.

## Discussion

**Fig 8:**
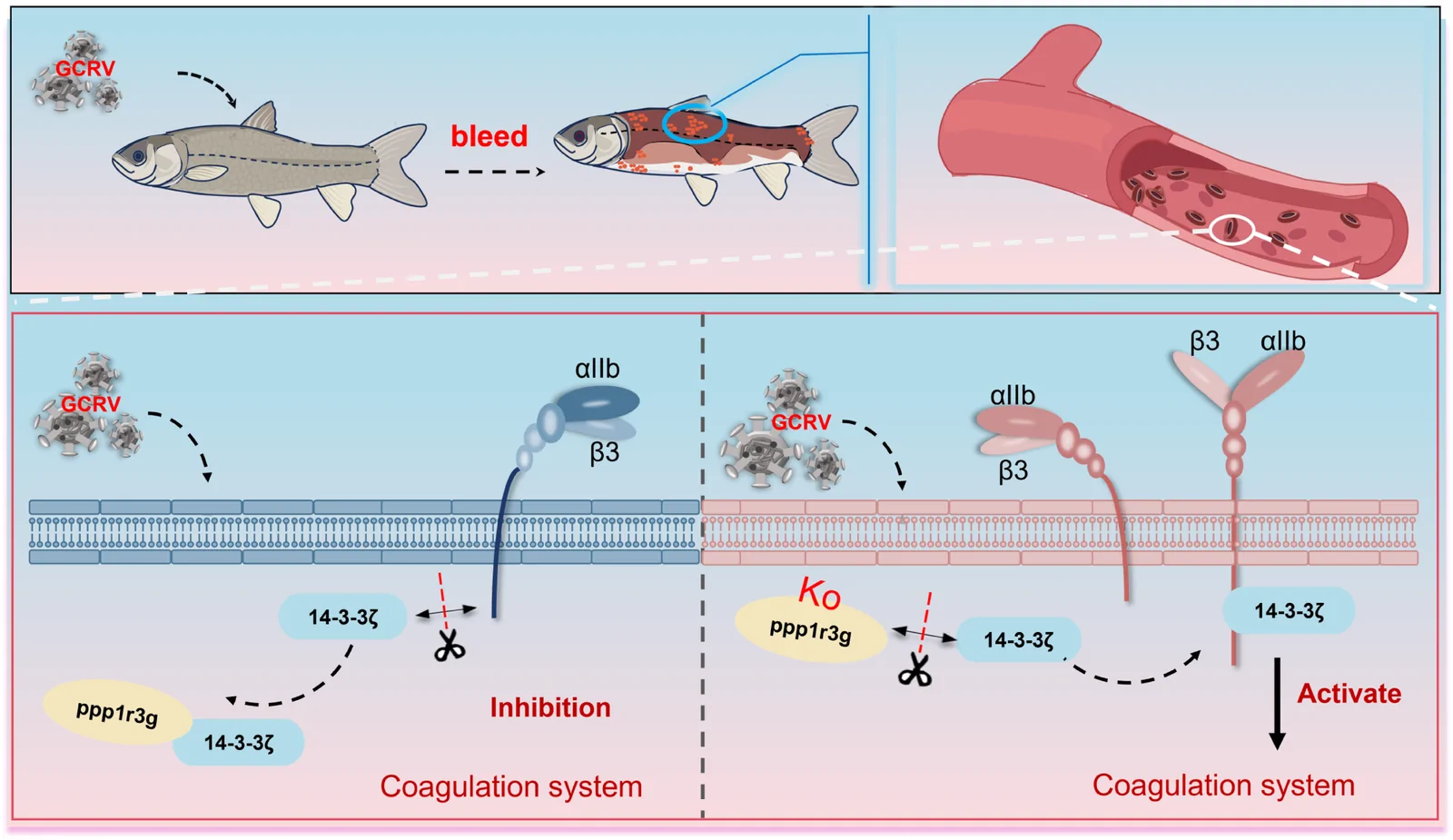
Model explaining the Ppp1r3g-mediated negative regulation of platelet and coagulation system activation in fish. Upon viral infection, Itgb3 undergoes a conformational change, facilitating its incorporation into the 14-3-3ζ-c-Src-integrin-β3 complex. This complex activates platelets and subsequently triggers the coagulation cascade. In contrast, Ppp1r3g, a regulatory subunit of PP1, interacts with 14-3-3ζ and promotes the degradation of Itgb3 via the proteasome pathway, thus suppressing platelet activation and the downstream coagulation response.

The coagulation and immune systems cooperate through multiple shared regulatory pathways, which play critical roles in host defense responses against invading pathogens [24,25,57]. These two systems are closely linked in evolution and function, forming a synergistic network to defend against pathogenic microbial invasion [26,27]. This study demonstrated that Ppp1r3g functions as a negative regulator, linking antiviral immunity and coagulation responses during GCRV infection. After GCRV invasion, Ppp1r3g directly interacted with some core components of the RLR pathway, including Rig-I and Mda5 and their downstream adaptor protein (Traf6). This interaction effectively suppresses IFN-I signaling and its downstream effector genes, negatively regulating the innate antiviral response of the host. Additionally, Ppp1r3g interferes with the formation of the 14-3-3ζ-Itgb3 complex by interacting with 14-3-3ζ, a crucial platelet-activating protein, inhibiting platelet activation and subsequent signaling and amplification of the coagulation cascade (Fig 8).

Previous studies have shown that *ppp1r3g* is involved in glycogen and lipid metabolism, insulin signaling, and inflammation regulation [50,52,58]. However, the role of Ppp1r3g in fish innate immunity has not been fully elucidated. In this study, *ppp1r3g*-deficient rare minnow and grass carp models were generated to elucidate the function of *ppp1r3g* in antiviral innate immune responses. After GCRV infection, *ppp1r3g^−/−^* rare minnow larvae exhibited increased survival rates, whereas adult fish exhibited delayed disease onset. Although the overall survival of *ppp1r3g*-chimera grass carp did not differ from that of the wild type, the hemorrhagic symptoms in the muscle tissues were markedly alleviated. Transcriptome analysis revealed that the expression levels of several antiviral genes, including *tlr3*, *irf3*, *mx*, *ifn1*, *isg15*, and *ifi56*, were upregulated in both models compared with those in WT controls. These findings indicate that *ppp1r3g* may function as a negative regulator of innate antiviral signaling in fish. As a regulatory subunit of PP1, PPP1R3G typically forms a holoenzyme with the PP1 catalytic subunit to exert its effects. PP1 is reported to be involved in innate antiviral immune responses [46,59]. Both the RLR signaling pathway and the TLR signaling pathway are essential for host defense responses against RNA virus infection. The PP1 catalytic subunit can promote antiviral signaling by dephosphorylating the CARD domains of RIG-I and MDA5 [47] and enhancing NF-κB-mediated innate immune responses through the regulation of TRAF6 and its substrate (IKKγ) [48]. Additionally, the PP1 regulatory subunit GADD34 (PPP1R15A) suppresses TLR signaling by promoting the dephosphorylation of TAK1 [60]. These findings indicate that PP1 regulatory subunits contribute to the modulation of innate immune pathways. Previously, we demonstrated that Ci-Ppp1r3g regulates IRF3 during GCRV-induced antiviral responses [53]. Based on these findings, Ppp1r3g selectively interacted with Rig-I, Mda5, and Traf6, whereas no interactions were detected with Mavs, Lgp2, Traf3, or Tbk1. Furthermore, the GS domain of Ppp1r3g was primarily responsible for mediating these interactions. This discovery expands the functional repertoire of Ppp1r3g and reveals a previously unrecognized role for this PP1 regulatory subunit in the regulation of antiviral innate immune responses in fish.

A characteristic feature of GCRV infection in fish is extensive hemorrhage across multiple organs and tissues, often accompanied by vascular wall disruption and circulatory necrosis [28,29,61]. The findings of this study indicate that the hemorrhagic symptoms in the superficial muscles and intact vascular walls in both *ppp1r3g*^−/−^ rare minnow and *ppp1r3g*-chimera grass carp were significantly alleviated when compared with those in WT individuals post-GCRV infection. The expression of coagulation factors, such as *f2*, *f3b*, *f9*, and *f10*, was upregulated in the tissues of both grass carp and rare minnow. In contrast, some anticoagulant factors, including *serpinc1*, *serpind1*, *and serpinf1*, were significantly downregulated. These findings indicate that *ppp1r3g* deficiency activates both extrinsic and intrinsic coagulation pathways, suppressing GCRV-induced hemorrhage, which is consistent with the findings of previous studies [28,62]. Recent studies have revealed that 14-3-3ζ regulates platelet activation by directly interacting with GPIbα and integrin αIIbβ3 (GPIIb/IIIa). Additionally, 14-3-3ζ modulates platelet activation *via* mitochondrial-mediated exposure to platelet PS [30,37,63]. In this study, Ppp1r3g could bind to 14-3-3ζ at the Ser36/37 phosphorylation site. The colocalization of Ppp1r3g and 14-3-3ζ was detected in the cytoplasm. Furthermore, this study demonstrated that Ppp1r3g could interact with 14-3-3ζ and Itgb3 and that Itgb3 binds to the -ESKVFYLKMKGDYYRYL- fragment (EL17) of 14-3-3ζ, which is consistent with the findings of a previous study [30]. This study also demonstrated that Ppp1r3g binds to the EL17 fragment of 14-3-3ζ, suggesting a competitive binding relationship among these proteins. Interestingly, GCRV infection weakened the interaction between 14-3-3ζ and Itgb3, indicating that viral infection may promote the dissociation of the 14-3-3ζ–Itgb3 complex. Given the essential role of Itgb3 in thrombocyte activation and hemostasis, disruption of this interaction may contribute, at least in part, to the hemorrhagic symptoms observed during GCRV infection. Furthermore, Ppp1r3g dose-dependently promoted the degradation of Itgb3 protein. Notably, Ppp1r3g did not induce the ubiquitination of Itgb3, indicating that Ppp1r3g-mediated Itgb3 degradation is ubiquitination independent and likely involves an alternative proteolytic mechanism. Viral infections are associated with economic losses to the aquaculture industry [64–66]. In this study, the hemorrhagic symptoms in both *ppp1r3g*^−/−^ rare minnow and *ppp1r3g*-chimera grass carp were significantly alleviated when compared with those in WT individuals after GCRV challenge. Additionally, *ppp1r3g*^−/−^ rare minnow and *ppp1r3g*-chimera grass carp exhibited enhanced resistance to GCRV infection. These findings suggest that Ppp1r3g is a potential therapeutic target for GCRV infection in fish. However, more studies are still needed to elucidate the molecular mechanisms underlying the antiviral effects of Ppp1r3g against GCRV infection.

## Materials and methods

### Ethics statement

The fish experiments were conducted at the Institute of Hydrobiology, Chinese Academy of Sciences, following the European Union guidelines for handling of laboratory animals (2010/63/EU). Healthy full-sib grass carp and rare minnows aged 6 months were acquired from the Institute of Hydrobiology. All the grass carp and rare minnow experiments were approved by the Institutional Animal Care and Use Committee of the Institute of Hydrobiology, Chinese Academy of Sciences.

### Cells and viruses

K562 cells were cultured in Roswell Park Memorial Institute-1640 medium supplemented with 10% fetal bovine serum (FBS). Grass carp kidney (CIK) and GCO were cultured in M199 medium (M-199) supplemented with 10% FBS. Human embryonic kidney (HEK293T) and K562 cells were cultured at 37 °C and 5% CO_2_, while CIK and GCO cells were cultured at 28 °C and 5% CO_2_. HEK293T cells were cultured in high-glucose Dulbecco’s modified eagle medium (Biosharp) supplemented with 10% TransSerum FQ FBS (TransGen Biotech). All the cell lines were cultured in a humidified incubator and were verified to be free of *Mycoplasma* contamination before use. GCRV (strain: GCRV-0901) was propagated in GCO cells until the CPE was complete. The culture medium containing GCRV (approximately 1.32 × 10^8^ TCID_50_/mL) was collected and stored at −80˚C until use. Type II grass carp reovirus (GCRV-HZ08) was diluted to a titer of 2.97 × 10^3^ RNA copies/μL for further experiments.

### Grass carp and rare minnow

In this study, *ppp1r3g*-null rare minnow (*Gobiocypris rarus*) were raised, maintained, and staged according to standard protocols. CRISPR/Cas9 was used to knock out *ppp1r3g* in rare minnow. First, *ppp1r3g* single-guide RNA (sgRNA) was designed using the CRISPR design tool (http://crispr.mit.edu). After digestion with *Xba*I, the rare minnow-codon-optimized Cas9 plasmid was purified and subsequently transcribed through the T7 mMessage mMachine kit (Invitrogen). The sgRNA template was amplified with the pUC19-gRNA vector. The following primers were used for guide RNA (gRNA) template amplification: 5′-GTAATACGACTCACTATAGGATCATTCGGGCGATGGGTGTTTTAGAGCTAGAAATAGC-3′ and 5′-AAAAGCACCGACTCGGTGCC-3′. The sgRNA was synthesized using a TranscriptAid T7 high-yield transcription kit (Thermo Fisher). At the one-cell stage, Cas9 RNA and sgRNA were mixed for intraembryonic injection (final concentrations: 500 and 80 ng/μL, respectively). The primers used to identify the mutants were as follows: 5′-TTCAAGATGCCCGTGGACTC-3′ (forward primer) and 5′-CTTTCAACGACTGGCTGTCG-3′ (reverse primer). Moreover, *ppp1r3g*-chimera grass carp (*Ctenopharyngodon idella*) were raised, maintained, and staged according to the standard protocols. The operating procedure has been previously described. The following primers were used for gRNA template amplification: 5′-GTAATACGACTCACTATAGGACGCGCTGGGGTTGAATCGTTTTAGAGCTAGAAATAGC-3′ and 5′-AAAAGCACCGACTCGGTGCC-3′. The primers used to identify the mutants were as follows: 5′-CGTAACCAGCGTGTAACTGAT-3′ (forward primer) and 5′-AGGTCATCCATGAAGTCCCG-3′ (reverse primer).

### Plasmid construction and reagents

Grass carp *ppp1r3g* (Ci-*ppp1r3g*) (GenBank: MT833844), grass carp *14-3-3ζ* (Ci-*14-3-3ζ*) (GenBank: PX962606), grass carp 14-3-3α (Ci-*14-3-3α*) (GenBank: KY742727.1), grass carp *itgb3* (Ci-*itgb3*) (GenBank: PX962609), rare minnow *ppp1r3g* (Gr-*ppp1r3g*) (GenBank: MT833843) rare minnow *14-3-3ζ* (Gr-*14-3-3ζ*) (GenBank: PX962605), rare minnow *14-3-3α* (Gr-*14-3-3α*) (GenBank: PX962607), and rare minnow *itgb3* (Gr-*itgb3*) (GenBank: PX962608), and the truncated mutants were amplified from rare minnow and grass carp complementary DNA (cDNA) using PCR. The amplified genes were subcloned and inserted into the pCMV-Myc (Clontech), pCMV-HA (Clontech), pCMV-Flag (Clontech), pAcGFP-N1 (Clontech), or pAcmCherry-N1 (Clontech) vectors. Cell transfection was performed using Neofect (Mayin Technology, Beijing, China). Moreover, cells were transfected with poly(I:C) (Glpbio, California, USA) using Lipofectamine 2000 transfection reagent (Invitrogen). The Ci-*ppp1r3g* and Gr-*ppp1r3g* genes were subsequently cloned and inserted into a PHAGE-puro-6tag cloning and expression lentivector provided by Prof. Wuhan Xiao (Institute of Hydrobiology, Wuhan, China). The vector was digested with *BamH*I and *Xho*I, followed by ligation with the inserts using the Uniclone one-step Seamless Cloning Kit (SC612, Genesand), according to the manufacturer’s instructions. The bacteria were plated on ampicillin agar plates overnight. The colonies were subsequently cultured in Luria-Bertani broth supplemented with ampicillin. The bacterial plasmid was purified using the EasyPure plasmid MiniPrep Kit (EM101, TRANS). The isolation of plasmids was confirmed using PCR with cloning primers. The fragments were gel-purified, and their sequences were confirmed using Sanger sequencing with the forward primer. The primer sequences are provided in the Supplementary S2 Table.

### Reagents and antibodies

Phenylmethylsulfonyl fluoride (329-98-6, Sigma) and MG132 (133407-82-6, MedChemExpress) were purchased from different vendors. The following antibodies were used: anti-Flag (66008–4-Ig, Proteintech Group, Inc.; diluted 1:10000), anti-Myc (sc-40, Santa Cruz Biotechnology; diluted 1:1000), anti-HA (66006–2-Ig, Proteintech Group, Inc.; diluted 1:10000), anti-β-actin (ABL1010, Abbkine; diluted 1:10000), and anti-14-3-3ζ (AtaGenix, Customized antibody; diluted 1:1000) antibodies. Anti-Flag/Myc/HA magnetic beads were purchased from Biolinkedin (L-1011A, L-1010A and L-1009).

### siRNA-mediated knockdown of *ppp1r3g*

CIK cells were transfected with siRNA targeting Ci-*ppp1r3g*. The following three siRNA sequences targeting different regions of Ci-*ppp1r3g* were synthesized by GenePharma (Jiangsu, China): si-Ci-*ppp1r3g*: CCATGTACACGCCTCCTTT (all sense 5′-3′). CIK cells were transfected with siRNA using Lipofectamine 2000 Transfection Reagent (Invitrogen) for 24 h. The knockdown efficiencies of siRNA constructs were evaluated using qRT-PCR and compared with those of the negative control siRNA (si-NC) provided by the supplier.

### Viral infection and plaque assays

To perform survival ratio assays, rare minnow larvae (n = 30; 3 dpf) were placed in a 6-well plate. Next, 4 mL of water containing GCRV (24 mL of water plus 300 μL of GCRV (2.97 × 10^3^ RNA copies/μL)) was added to each well. The survival ratio was monitored every 2 h over a 72-h period. To infect adult rare minnows with the virus, rare minnows (n = 26; 3 mpf) were infected with GCRV (2.97 × 10^3^ RNA copies/μL) for 20 min. Adult rare minnows exposed to 0.9% physiological saline served as the control. During the 10-day observation period, the survival rate of rare minnows were monitored once every 24 hours. To perform the virus infection experiment for grass carp larvae, the soaking challenge method was used to infect grass carp lavae (n = 48; 6 mpf) with GCRV (2.97 × 10^3^ RNA copies/μL) for 20 min. Grass carp larvae exposed to 0.9% physiological saline served as the control. During the 35-day observation period, the survival rate of rare minnows was monitored once every 24 h. To perform the plaque assay, CIK cells were transfected with si-*ppp1r3g* or si-NC. At 24 h post-transfection, the cells were infected with GCRV for 24 h at the indicated dose. The culture supernatant of CIK cells infected with GCRV (multiplicity of infection = 0.2) was collected to determine viral titers. The cells were subsequently washed with phosphate-buffered saline (PBS), fixed with 4% paraformaldehyde, and stained with 1% crystal violet to visualize the CPE.

### Virus titer determination

CIK cells were cultured in 96-well plates. The culture supernatant (containing virus) was serially diluted (10^−1^ to 10^−8)^ in sterile 1.5 mL tubes using M-199 medium. The diluted virus samples were incubated with CIK cells for 7 days. The plates were observed under a microscope, and the results were considered positive if the area of detached cells in one well after infection with the virus was 50%. The titers for GCRV infection were calculated using the Spearman-Ka¨rber method and represented as the 50% tissue culture infective dose (TCID_50_). The experiments were repeated three times for statistical analysis.

### Quantitative real-time PCR analysis

Total RNA was extracted from cells and tissues (n = 3) using the AG RNAex Pro Reagent (AG21101, Accurate Biology) following the manufacturer’s instructions. Equivalent amounts of total RNA (1 μg) were reverse-transcribed into cDNA using All-in-First-Strand Synthesis MasterMix (Biology Biotechnology, WuHan, China). qRT-PCR analysis was performed using Fast SYBR Green PCR Master mix (Bio-Rad) with the StepOne Real-Time PCR System (Applied Biosystems). Each experiment was independently repeated at least three times. The sequences of primers used for the qRT-PCR analysis of procoagulant and anticoagulant factors were obtained from previous studies [28,62]; all the qRT-PCR sequences of primers used in this study are listed in S2 Table.

### Co-immunoprecipitation and western blotting

HEK293T cells were seeded overnight in 100-mm cell culture dishes and transfected with the indicated plasmids (10 μg per dish). At 24 h post-transfection, the cells were washed with ice-cold PBS and lysed in 1 mL of radioimmunoprecipitation assay buffer. The supernatant was transferred to a new tube and subjected to immunoprecipitation with anti-Flag/Myc/HA magnetic beads. Total cell lysates and immunoprecipitated samples were subjected to western blotting analysis.

### Ubiquitination assay

GCO cells were co-transfected with plasmids encoding His-tagged ubiquitin (His-Ub) and the indicated combinations of Myc-Gr-Itgb3 and Flag-Gr-Ppp1r3g or Flag-Ci-Itgb3 and Myc-Ci-Ppp1r3g. At 18 h post-transfection, the cells were treated with MG132 (20 μM) for 6 h and harvested at 24 h post-transfection. Cell lysates were prepared by sonication and incubated with Ni-NTA agarose beads overnight at 4 °C. After extensive washing, the bound proteins were analyzed by immunoblotting using anti-Flag and anti-Myc antibodies. The expression of the indicated proteins in whole-cell lysates was confirmed by immunoblotting.

### Immunofluorescence confocal microscopy

GCO cells cultured on glass coverslips were fixed with 4% paraformaldehyde for 25 min, washed three times with ice-cold PBS, permeabilized with 0.05% Triton X-100, and washed three times with ice-cold PBS. The nuclei were count erstained with 4,6-diamidino-2-phenylindole (DAPI; C0065, ServiceBio). After being washed five times with ice-cold PBS, the cells were imaged using a Leica laser-scanning confocal microscope.

### Immunohistochemistry

The ovarian tissue was sliced into 4 μm-thick sections and paraffinized. The sections were then deparaffinized with deparaffinization buffer (G1128, ServiceBio), rehydrated in ethanol, heated with sodium citrate buffer (pH 6.0; G1202, ServiceBio) for 20 min to retrieve antigens, and cooled to room temperature. Next, the sections were incubated in a 3% methanol-hydrogen peroxide solution at room temperature in the dark for 25 min, washed three times with PBS, and blocked with 3% bovine serum albumin (BSA) at room temperature for 30 min; next, the sections were incubated with an anti-14-3-3ζ antibody (AtaGenix, customized antibody; diluted 1:100) overnight at 4 °C, and probed with a secondary antibody (S-vision immunohistochemistry polyclonal antibody; G1303, ServiceBio; diluted 1:300) for 50 min. Further, the sections were stained with freshly prepared 3-amino-9-ethylcarbazole (G1264-200T, ServiceBio) in the dark for 10–25 min. The nuclei were counterstained with hematoxylin (G1004, ServiceBio), followed by incubation with hematoxylin differentiation solution (G1039, ServiceBio) and hematoxylin counterstain solution (G1040, ServiceBio). The sections were mounted with glycerol-gelatin aqueous mounting medium (G1402, ServiceBio) and imaged under a Nikon E100 microscope.

### Immunofluorescence staining

The intestinal tissues were sliced into 4 μm-thick sections and embedded in paraffin. The paraffinized sections were deparaffinized with deparaffinization buffer (G1128, ServiceBio), rehydrated in ethanol, heated with sodium citrate buffer (pH 6.0, G1202, ServiceBio) for 20 min to retrieve the antigens, and cooled to room temperature. Next, the sections were incubated in a 3% methanol-hydrogen peroxide solution at room temperature in the dark for 25 min. After being rinsed three times with PBS, the sections were blocked with a 3% BSA solution at room temperature for 30 min. The sections were subsequently incubated overnight with an anti-14-3-3ζ primary antibody (AtaGenix, Customized Antibody; diluted 1:100) at 4 °C, followed by incubation with a secondary antibody (S-vision immunohistochemical polymer secondary antibody; G1303, ServiceBio; diluted 1:300) at room temperature for 50 min. The nuclei were counterstained with DAPI (C0065, ServiceBio) for 5 min in the dark and then were washed three times with Tris-buffered saline containing 0.05% Tween-20. The sections were mounted using antifade fluorescence mounting medium (G1401, ServiceBio) and stored at 4 °C in the dark. The stained sections were imaged using a fluorescence microscope (Nikon Eclipse C1, Nikon, Japan).

### Histopathological analysis

The muscle tissues were sliced into 4 μm-thick sections and embedded in paraffin. The sections were then deparaffinized with deparaffinization buffer (G1128, ServiceBio), rehydrated in ethanol, incubated with a high-definition constant-staining pretreatment solution, stained with hematoxylin solution, differentiated with differentiation solution, and incubated with a bluing solution. Finally, the sections were stained with an eosin solution (G1076, ServiceBio), dehydrated, and mounted. The images were captured and processed using ImageJ software.

### Lentivirus production and cell line transduction

HEK293T cells were co-transfected with PHAGE-puro-Ci-Ppp1r3g/Gr-Ppp1r3g, PSPAX2, and PMD2G using Neofect DNA transfection reagent (TF20121201, Neofect (Beijing) Biotech Co., Ltd.). At 24 and 48 h post-transfection, the virus-containing supernatant (10 mL) was collected and concentrated using a universal virus concentration kit (C2901M, Beyotime). The virus solution was purified and aliquoted into 200 μL per tube and stored in a −80 °C. K562 cells, which were seeded in plates the previous night, were incubated with 200 μL of concentrated virus solution and 10 μL of polybrene (28728-55-4, Beyotime). At 72 h post-treatment, some surviving cells were subjected to western blotting analysis. The cells stably expressing Ci-Ppp1r3g and Gr-Ppp1r3g were passaged for subsequent experiments.

### IP-MS analysis

The bead samples were incubated in reaction buffer (1% sodium deoxycholate/100 mM Tris-HCl (pH 8.5), 10 mM Tris (2-carboxyethyl) phosphine, and 40 mM 2-chloroacetamide) at 95 °C for 10 min for protein denaturation, cysteine reduction, and alkylation. The eluates were diluted with an equal volume of H_2_O and digested with trypsin (1:50; enzyme: protein; w/w) overnight at 37 °C. The pH was adjusted to 6.0 using TFA to terminate the digestion. The samples were subsequently centrifuged at 12000 g for 15 min. The peptides were subsequently purified using in-house SDB desalting columns. The eluate was vacuum-dried and stored at −20 °C until use.

All the samples were analyzed using an UltiMate 3000 RSLCnano system coupled online with a Q Exactive HF mass spectrometer through a Nanospray Flex ion source (Thermo). The MS raw data were analyzed with MaxQuant using the Andromeda database search algorithm. Proteins denoted as decoy hits, contaminants, or only identified by sites were removed. The remaining proteins were used for further quantification analysis. Proteins with a fold change > 4 between the bait immunoprecipitated and control samples were screened out as interactors of the bait protein. The Gene Ontology (GO) annotation files (released in July 2020) were downloaded from the Gene Ontology Consortium website (http://www.geneontology.org/). The Kyoto Encyclopedia of Genes and Genomes (KEGG) annotation files (released in July 2020) were downloaded from the ftp server of KEGG (ftp://ftp.bioinformatics.jp/). A two-sided hypergeometric test was adopted for the enrichment analysis of the interactors. GO terms and KEGG pathways with a *P* value of < 0.05 were considered significantly different among the interactors.

### Statistical analysis

All the statistical analyses (unpaired t-tests) were performed using GraphPad Prism (version 9.1). The data are representative of at least three independent experiments. The error bars indicate the standard deviation. Rare minnow and grass carp survival rates were represented using Kaplan-Meier curves. The survival curves were compared using log-rank analysis. Differences were considered significant at *P* < 0.05 (^*^P <0.05, ^**^P <0.01, ^***^P <0.001, ^***^P < 0.0001).

## Supporting information

S1 Fig Interaction of Ci-Ppp1r3g with components of the RLR signaling pathway. (A-D) Co-immunoprecipitation analysis of the interactions between Ci-Ppp1r3g and Ci-Traf3, Ci-Lgp2, Ci-Tbk1, and Ci-Mavs in HEK293T cells. Cells were co-transfected with the indicated expression plasmids, and cell lysates were analyzed by Co-IP and immunoblotting with the indicated antibodies. (E-G) Co-immunoprecipitation analysis of the interactions between Ci-Ppp1r3g and Ci-Traf6, Ci-Mda5, and Ci-Rig-I in GCO cells. Cells were co-transfected with the indicated expression plasmids, and cell lysates were analyzed by Co-IP and immunoblotting with the indicated antibodies.
(TIF)

S2 Fig Detection of *ppp1r3g* in knockout rare minnow and grass carp. (A) RNA was extracted from the livers of both mutant rare minnow and wild-type (*ppp1r3g*^+/+^) individuals. The mRNA expression levels of *ppp1r3g* were determined using quantitative real-time polymerase chain reaction (qRT-PCR) analysis. (B) RNA was extracted from multiple tissues of *ppp1r3g*-chimera grass carp and wild-type (WT) grass carp. The mRNA expression levels of *ppp1r3g* were determined using qRT-PCR analysis. (A-B) All the data represent the mean of three independent replicates. The error bars indicate the standard deviation (n = 3). ns, not significant; **P < 0.01, ****P < 0.0001.
(TIF)

S3 Fig GCRV infection disrupts the interaction between 14-3-3ζ and Itgb3. (A-B) GCO cells were co-transfected with the indicated plasmids. At 24 h post-transfection, the cells were infected with GCRV (MOI = 0.2) for an additional 12 h. Cell lysates were immunoprecipitated with anti-Flag (A) or anti-Myc (B) magnetic beads and analyzed by immunoblotting using anti-HA and anti-Myc antibodies (A) or anti-HA and anti-Flag antibodies (B).
(TIF)

S4 Fig Ppp1r3g promotes Itgb3 degradation. (A-B) Dose-dependent degradation of Gr-Itgb3 or Ci-Itgb3 induced by Gr-Ppp1r3g or Ci-Ppp1r3g. GCO cells were transfected with the indicated plasmids, and cell lysates were analyzed by immunoblotting at 24 h post-transfection using the indicated antibodies.
(TIF)

S5 Fig Ppp1r3g promotes Itgb3 degradation without affecting 14-3-3ζ expression. (A-B) HEK293T cells were co-transfected with the indicated plasmids, and cell lysates were analyzed by immunoblotting at 24 h post-transfection using the indicated antibodies.
(TIF)

S6 Fig Effects of 3-MA and NH_4_Cl treatment on Ppp1r3g-mediated Itgb3 degradation. (A-B) HEK293T cells were transfected with the indicated plasmids and treated 18 h post-transfection with increasing concentrations of 3-MA (5, 10, and 20 mM). The cell lysates were analyzed by immunoblotting at 24 h post-transfection using the indicated antibodies. (C-D) HEK293T cells were transfected with the indicated plasmids and treated 18 h post-transfection with increasing concentrations of NH_4_Cl (5, 10, and 20 mM). Cell lysates were analyzed by immunoblotting at 24 h post-transfection using the indicated antibodies.
(TIF)

S7 Fig Effect of Ppp1r3g on Itgb3 ubiquitination. (A-B) GCO cells were co-transfected with the indicated plasmids together with His-tagged ubiquitin (His-Ub). At 18 h post-transfection, cells were treated with MG132 (20 μM) for 6 h and harvested at 24 h post-transfection. The cell lysates were subjected to Ni-NTA pull-down followed by Western blot analysis to assess Itgb3 ubiquitination. Input samples were analyzed by Western blotting to confirm protein expression.
(TIF)

S8 Fig Impact of *ppp1r3g*-mediated *itgb3* degradation on the expression of IFNs and ISGs. (A-C) GCO cells were transfected with the indicated plasmids (empty vector, Ppp1r3g, Itgb3, or Ppp1r3g plus Itgb3) and subsequently infected with GCRV. Total RNA was extracted and subjected to qRT-PCR analysis. The mRNA expression levels of *itgb3*, *vp2*, *vp7*, *ifn-γ2*, *ifn3*, *isg15*, and *rig-i* were determined. All the data represent the mean of three independent replicates. The error bars indicate the standard deviation (n = 3). ns, not significant; *P < 0.05, **P < 0.01, ****P < 0.0001.
(TIF)

S1 Table Ppp1r3g interacts with 14-3-3 family proteins. (XLSX)

S2 Table The primer sequences. (DOCX)

S1 Data Raw data values. (XLSX)

S2 Data Rawgel. (PDF)

S3 Data Raw photographs. (PDF)
