# Supplementary material for: Grass carp reovirus activates fish Ppp1r3g to suppress innate immunity and exacerbate hemorrhage via the 14-3-3ζ/Itgb3 signaling axis
Source: PLoS Pathog. 2026 Sep 18;22(9):e1014590. doi: 10.1371/journal.ppat.1014590 (PMC13588375; doi:10.1371/journal.ppat.1014590)

Fig 1D

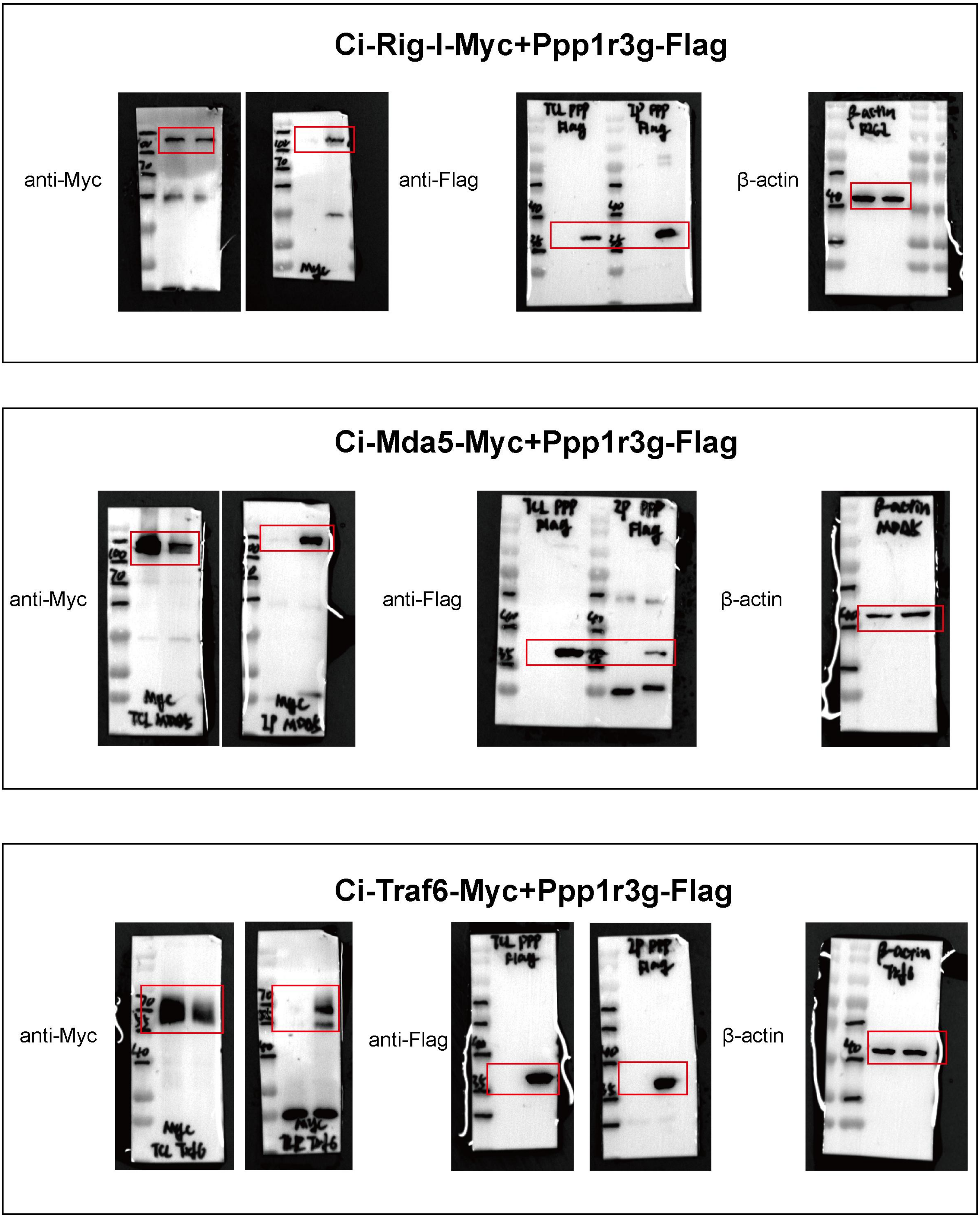
Ci-Rig-I-Myc+Ppp1r3g-Flag
anti-Myc
anti-Flag
β-actin
Ci-Mda5-Myc+Ppp1r3g-Flag
anti-Myc
anti-Flag
β-actin
Ci-Traf6-Myc+Ppp1r3g-Flag
anti-Myc
anti-Flag
β-actin

Fig 1D

Ci-Rig-I-Mcy+Ppp1r3g-Flag/Ppp1r3g-GS/PP1c-Flag

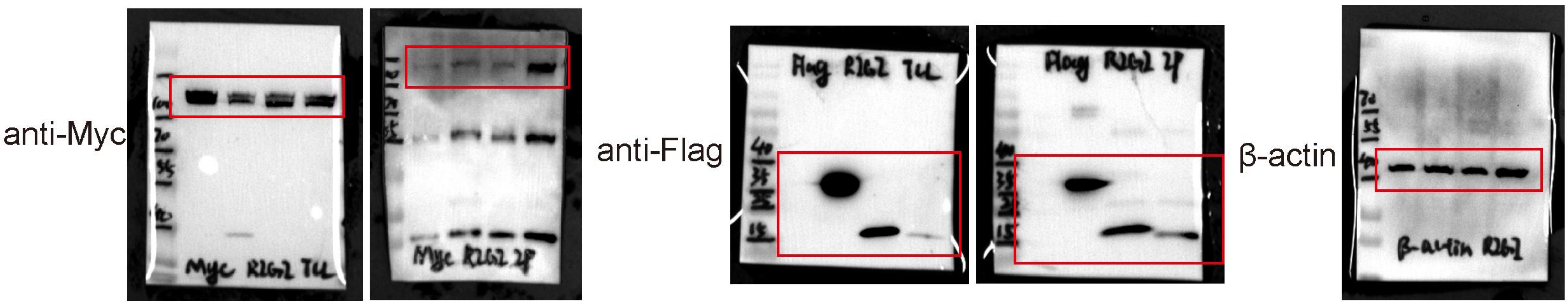

Ci-Mda5-Mcy+Ppp1r3g-Flag/Ppp1r3g-GS/PP1c-Flag

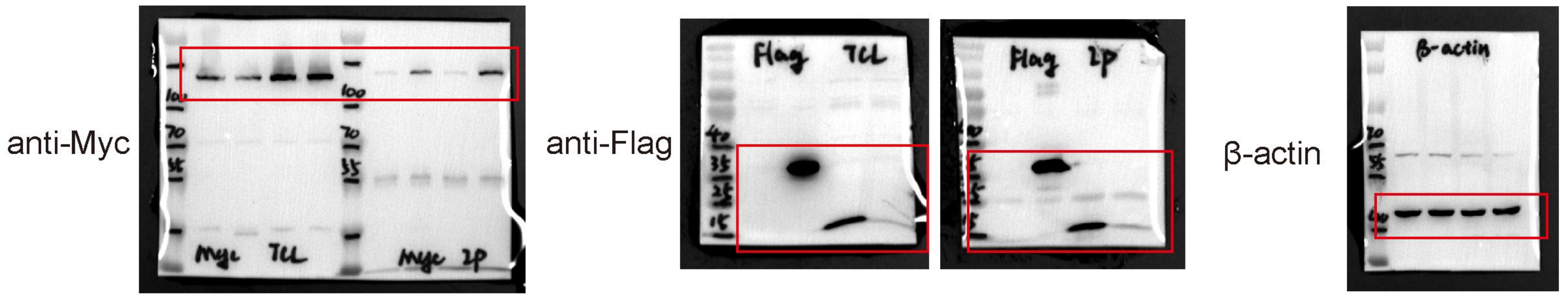

Ci-Traf6-Mcy+Ppp1r3g-Flag/Ppp1r3g-GS/PP1c-Flag

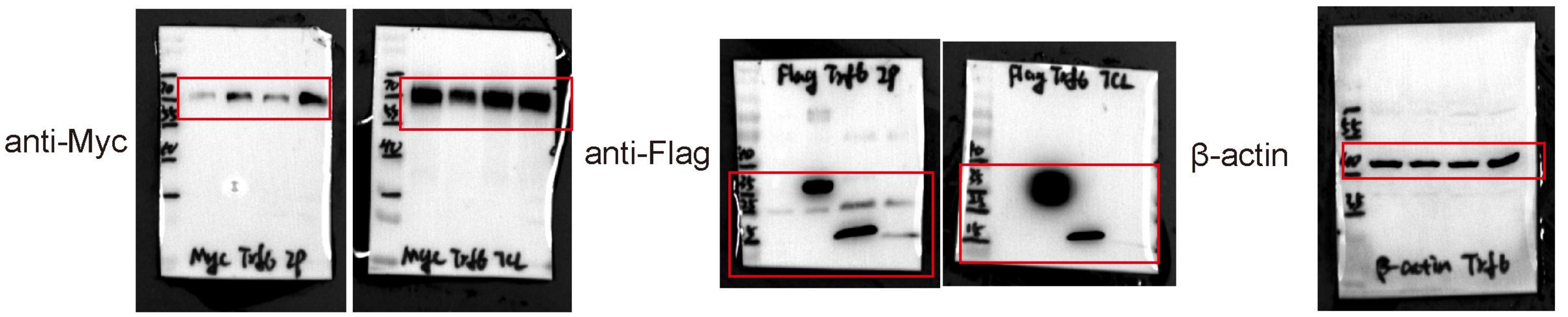

Fig 4A

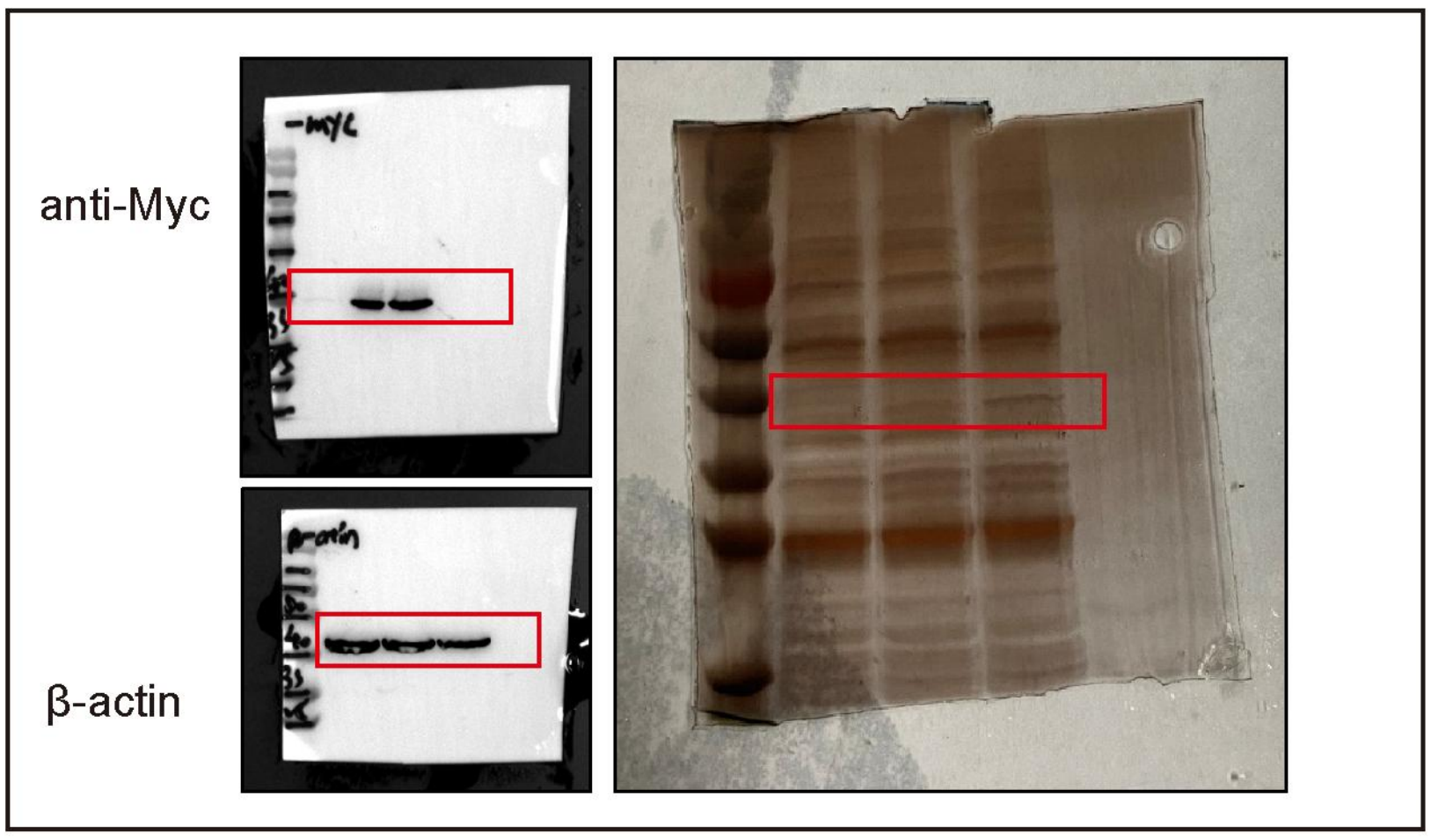
anti-Myc
β-actin

Fig 4C

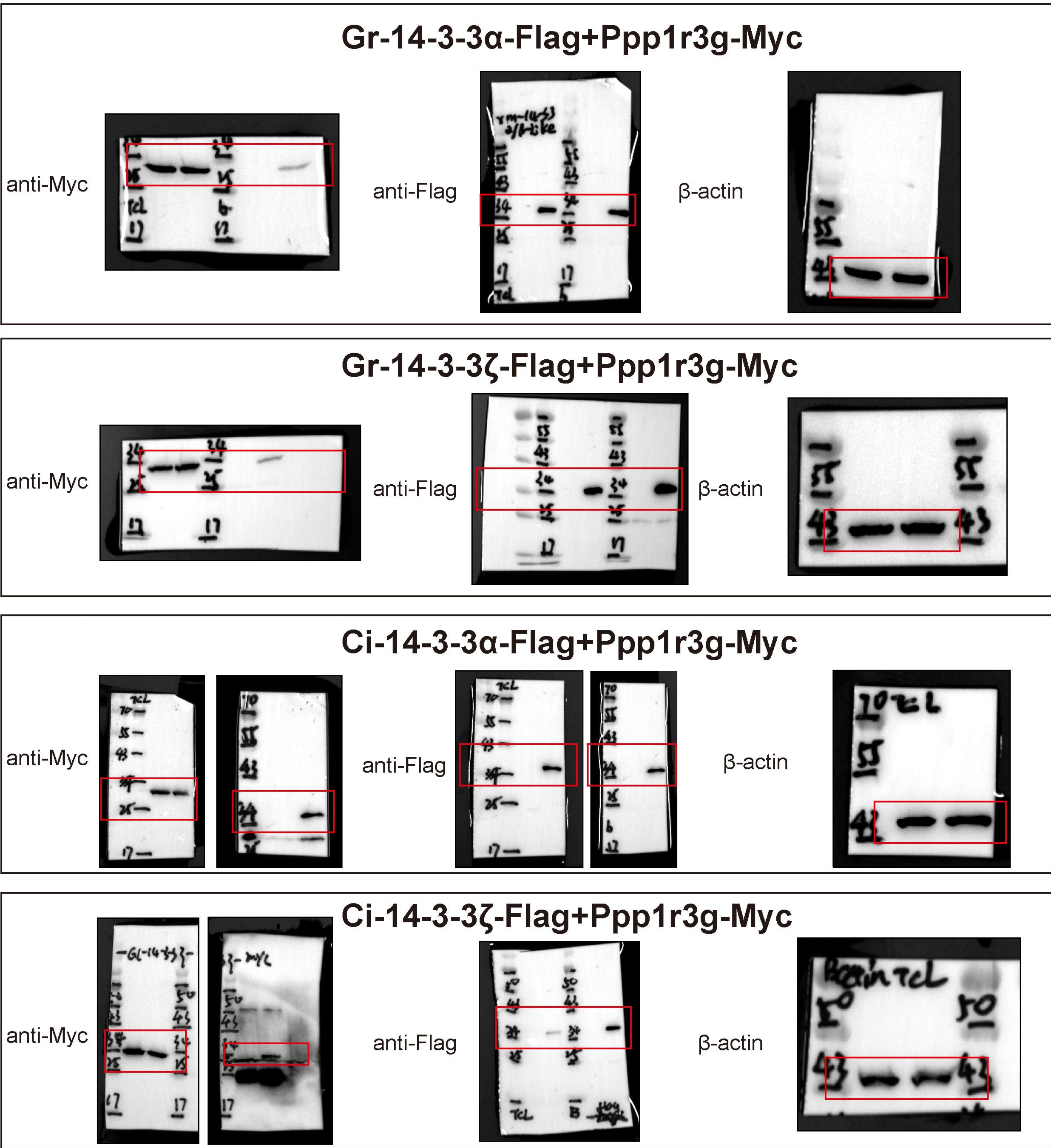
Gr-14-3-3α-Flag+Ppp1r3g-Myc
anti-Myc
anti-Flag
β-actin
Gr-14-3-3ζ-Flag+Ppp1r3g-Myc
anti-Myc
anti-Flag
β-actin
Ci-14-3-3α-Flag+Ppp1r3g-Myc
anti-Myc
anti-Flag
β-actin
Ci-14-3-3ζ-Flag+Ppp1r3g-Myc
anti-Myc
anti-Flag
β-actin

Fig 5A

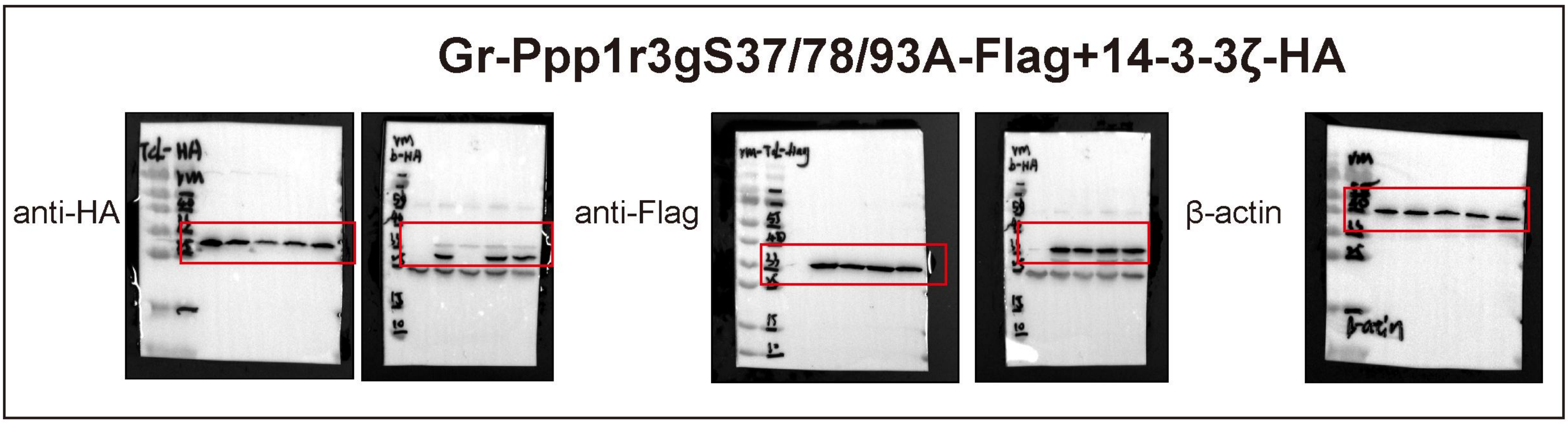
Gr-Ppp1r3gS37/78/93A-Flag+14-3-3ζ-HA
anti-HA
anti-Flag
β-actin

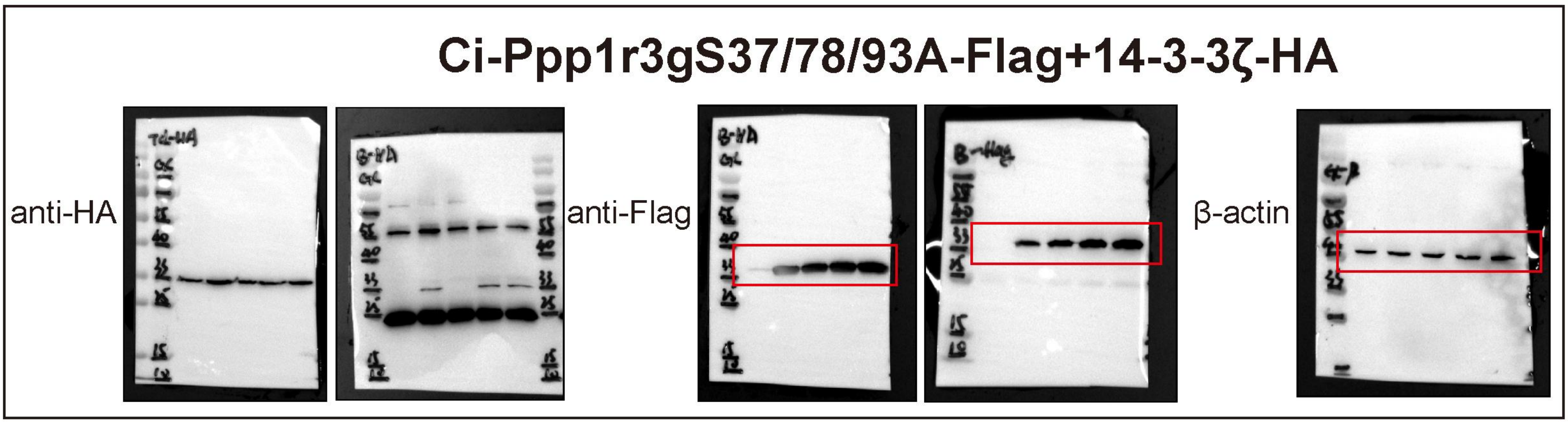
Ci-Ppp1r3gS37/78/93A-Flag+14-3-3ζ-HA
anti-HA
anti-Flag
β-actin

Fig 5B

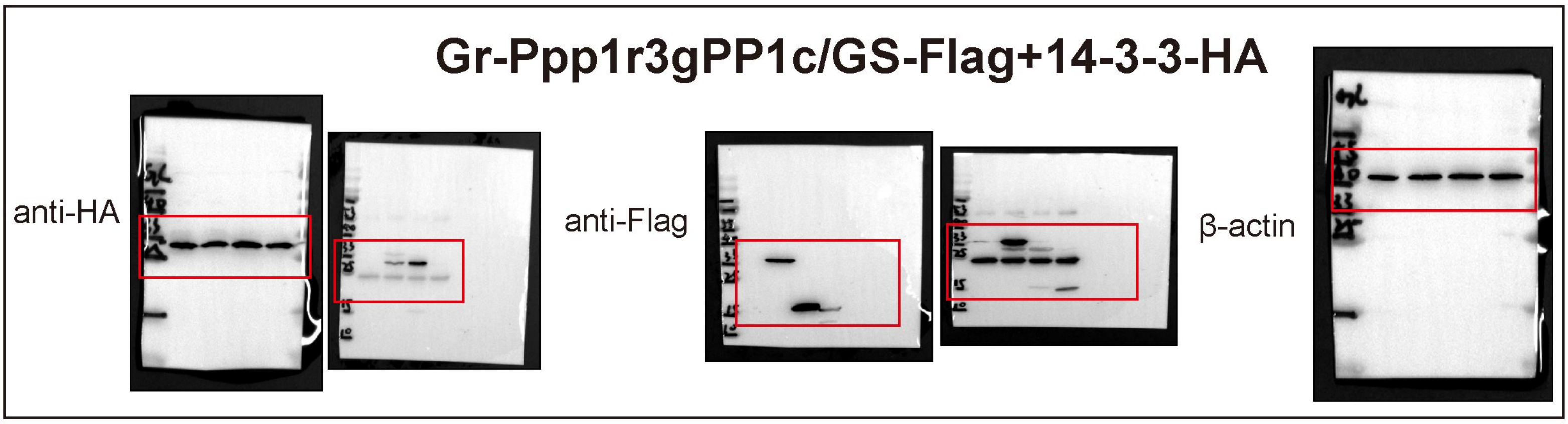
Gr-Ppp1r3gPP1c/GS-Flag+14-3-3-HA
anti-HA
anti-Flag
β-actin

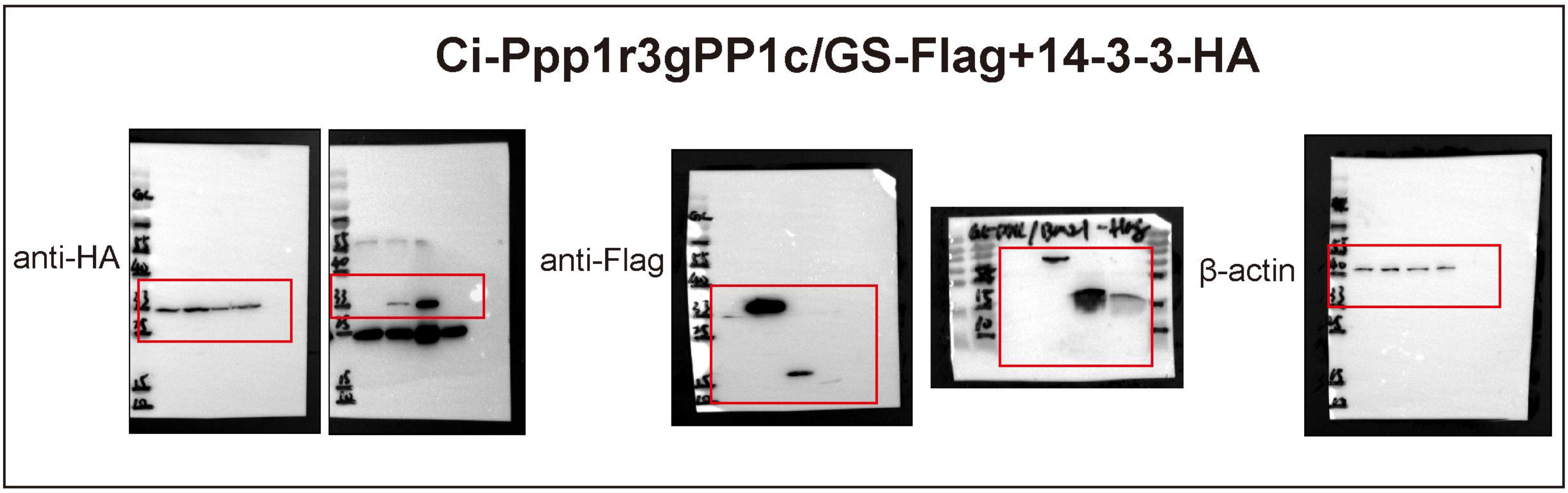
Ci-Ppp1r3gPP1c/GS-Flag+14-3-3-HA
anti-HA
anti-Flag
β-actin

Fig 6A

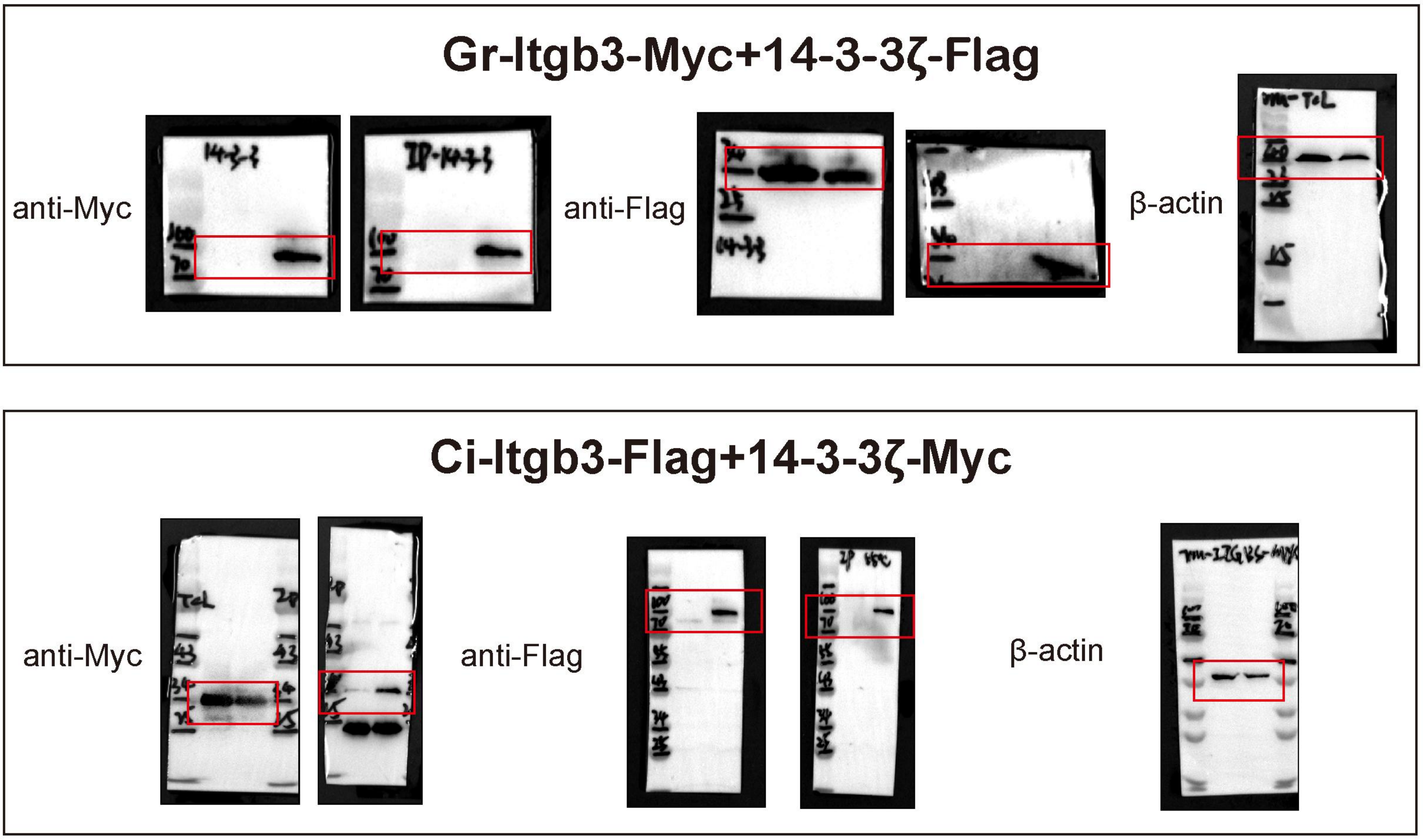
Gr-Itgb3-Myc+14-3-3ζ-Flag
anti-Myc
anti-Flag
β-actin
Ci-Itgb3-Flag+14-3-3ζ-Myc
anti-Myc
anti-Flag
β-actin

Fig 6B

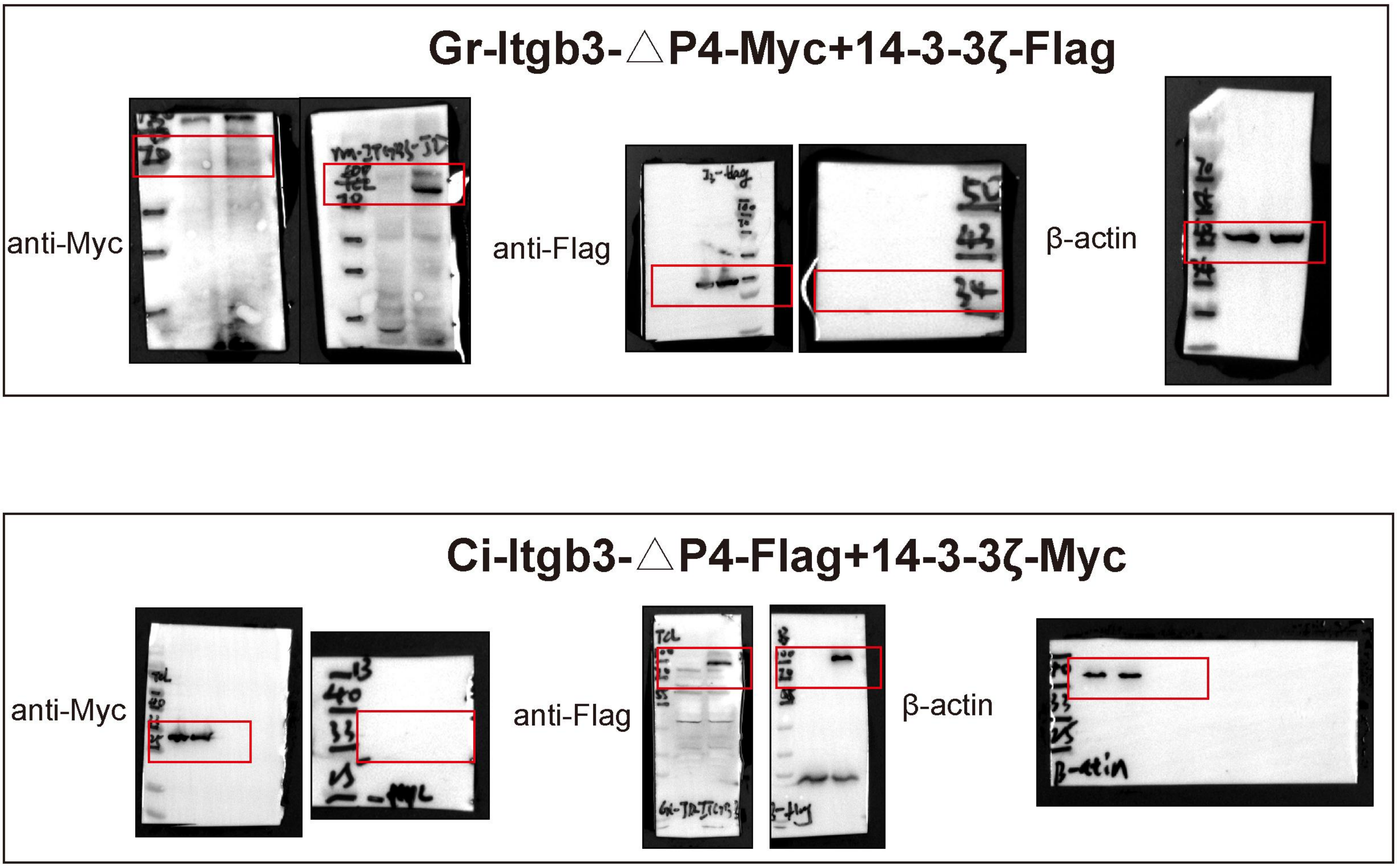
Gr-Itgb3-△P4-Myc+14-3-3ζ-Flag
anti-Myc
anti-Flag
β-actin
Ci-Itgb3-△P4-Flag+14-3-3ζ-Myc
anti-Myc
anti-Flag
β-actin

Fig 6C:

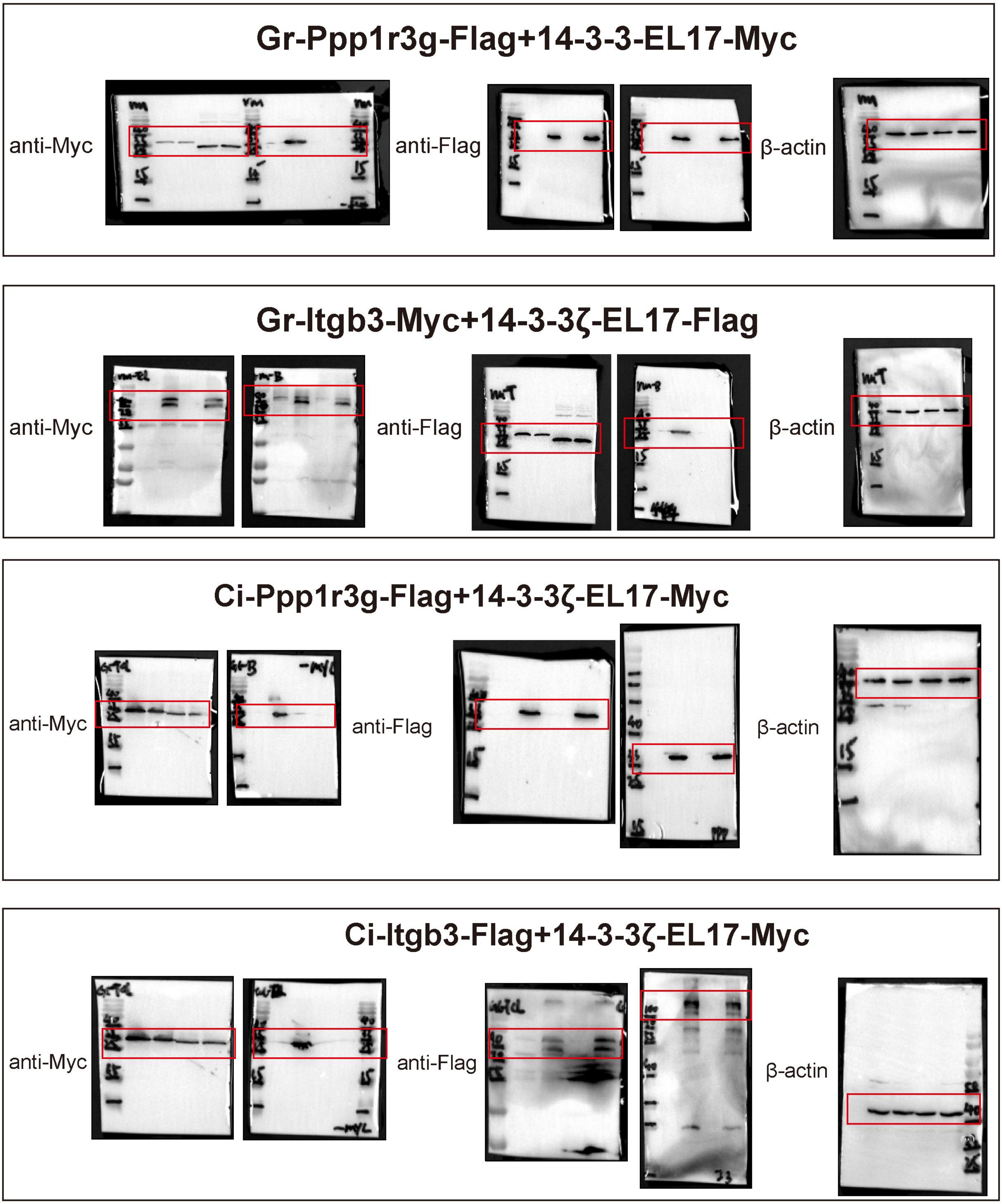
Gr-Ppp1r3g-Flag+14-3-3-EL17-Myc
anti-Myc
anti-Flag
β-actin
Gr-Itgb3-Myc+14-3-3ζ-EL17-Flag
anti-Myc
anti-Flag
β-actin
Ci-Ppp1r3g-Flag+14-3-3ζ-EL17-Myc
anti-Myc
anti-Flag
β-actin
Ci-Itgb3-Flag+14-3-3ζ-EL17-Myc
anti-Myc
anti-Flag
β-actin

Fig 7A:

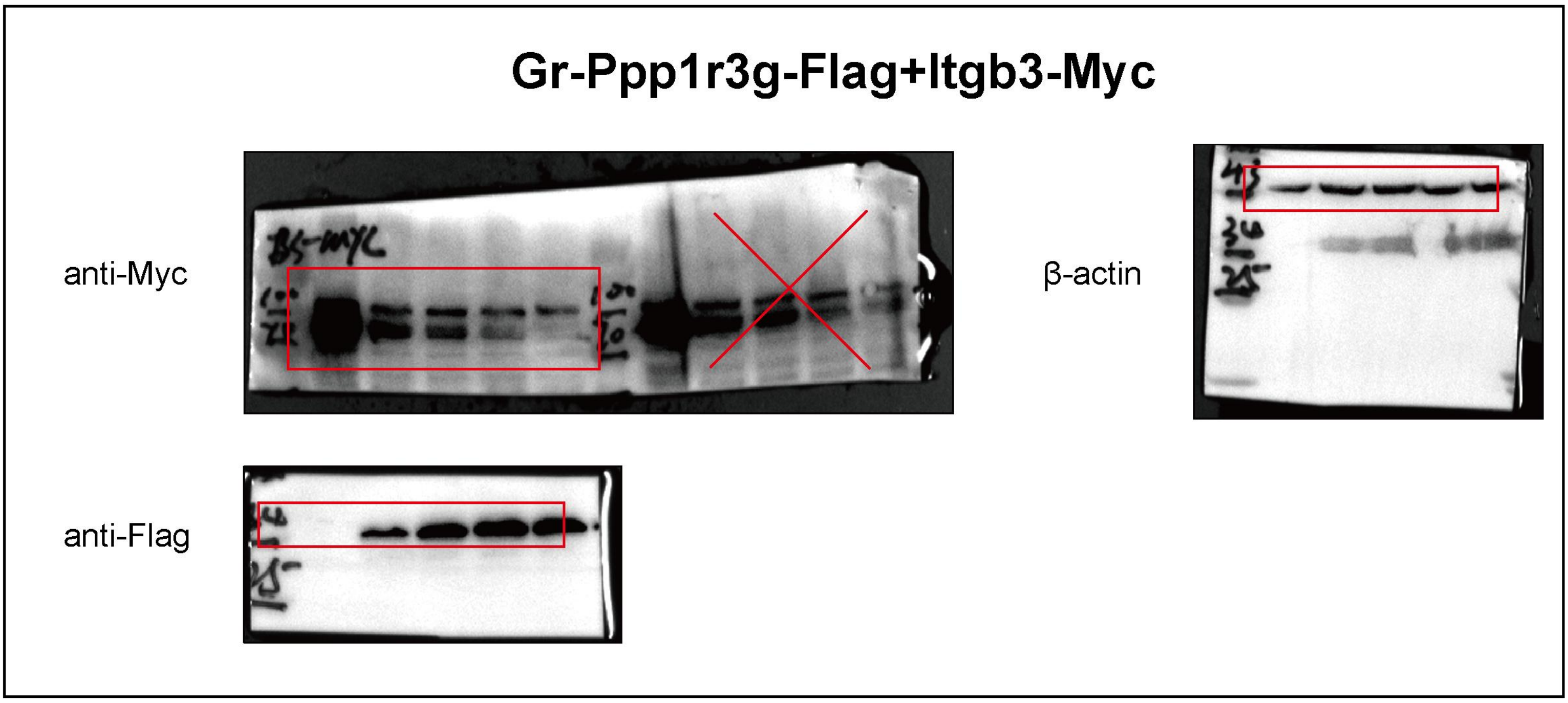
Gr-Ppp1r3g-Flag+Itgb3-Myc
anti-Myc
β-actin
anti-Flag

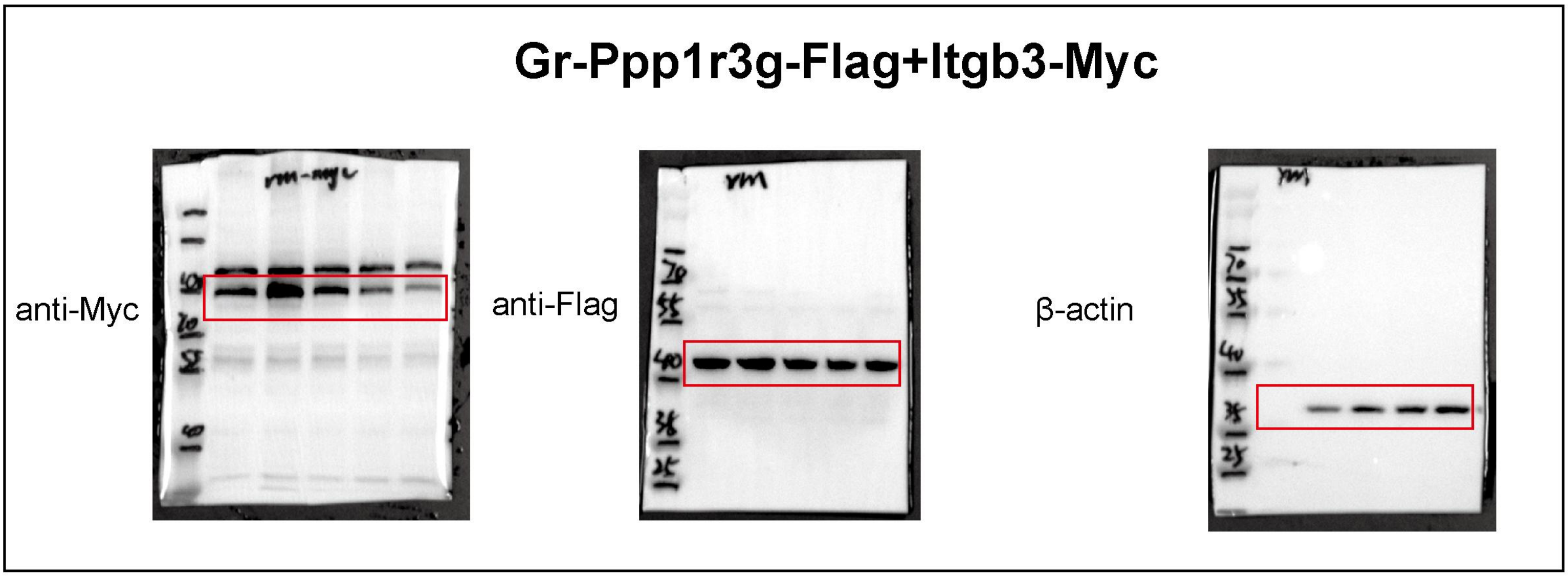
Gr-Ppp1r3g-Flag+Itgb3-Myc
anti-Myc
anti-Flag
β-actin

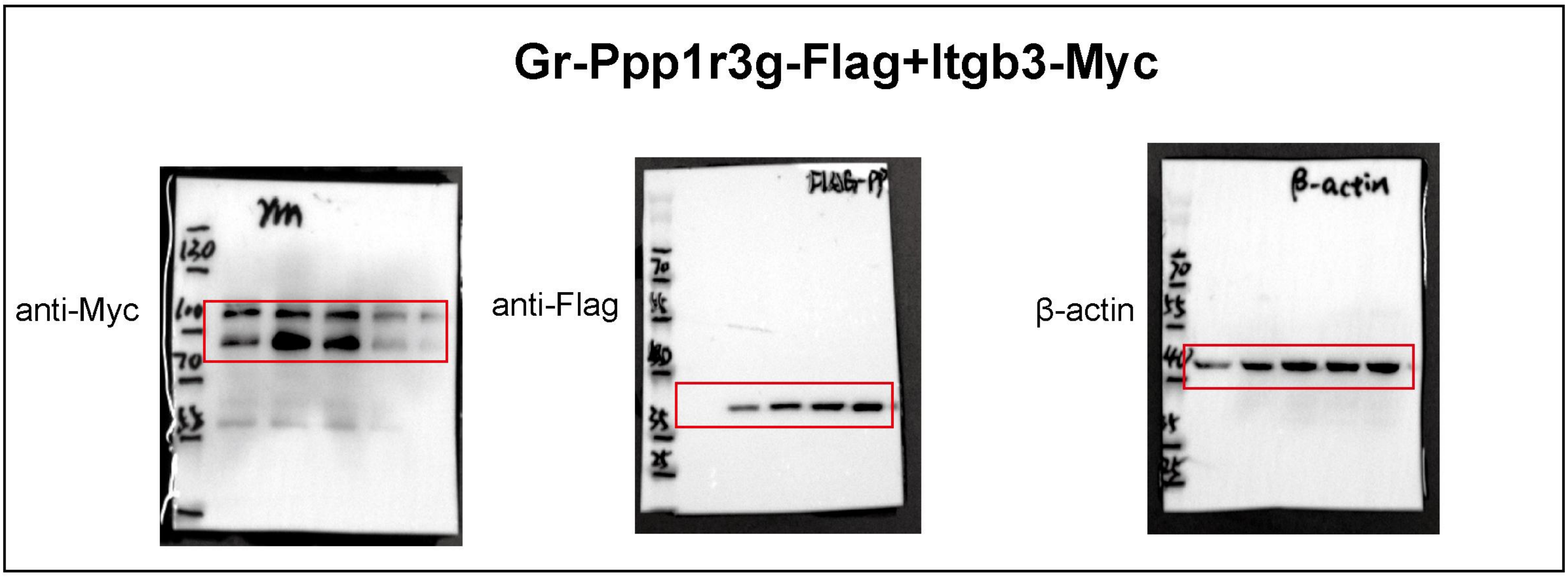
Gr-Ppp1r3g-Flag+Itgb3-Myc
anti-Myc
anti-Flag
β-actin

Fig 7B:

Ci-Ppp1r3g-Myc+Itgb3-Flag

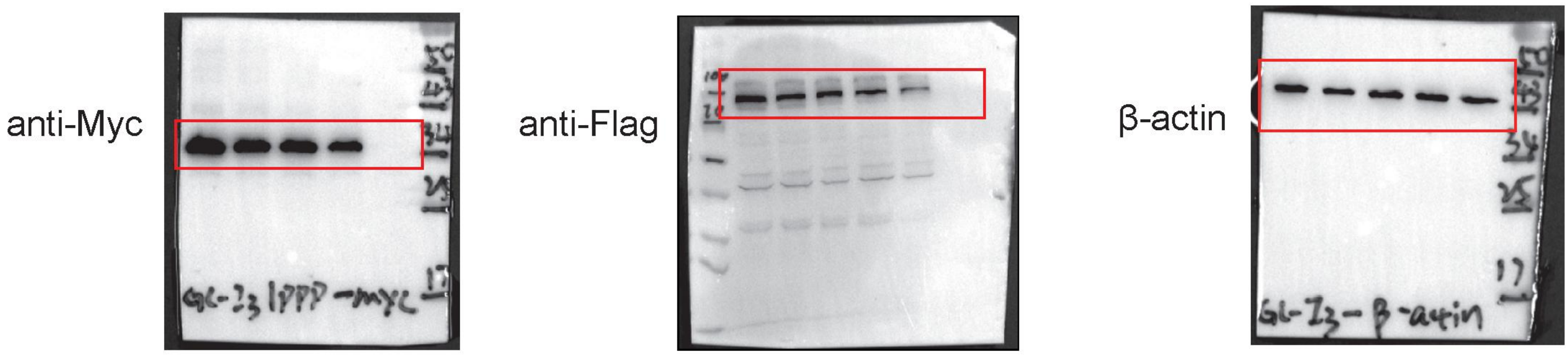

Gi-Ppp1r3g-Flag+Itgb3-Myc

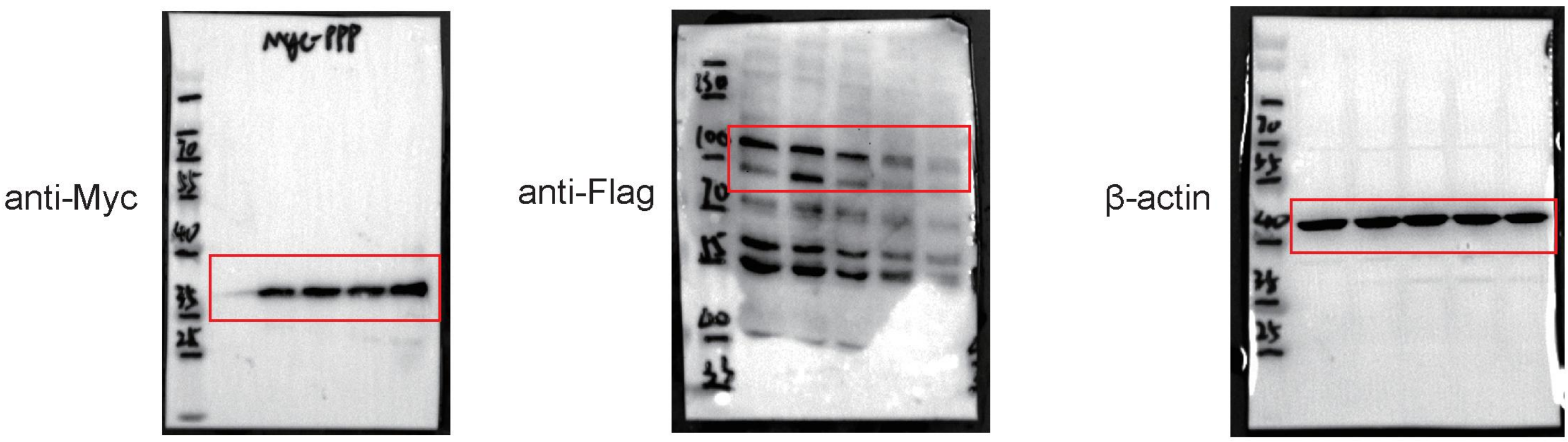

Gi-Ppp1r3g-Flag+Itgb3-Myc

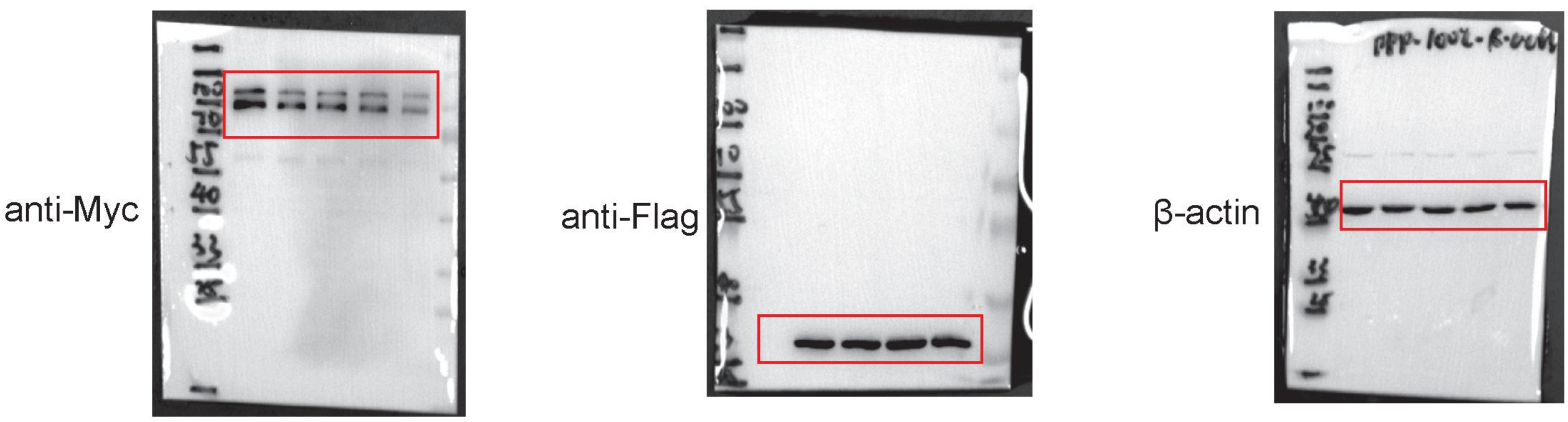

Fig 7C:

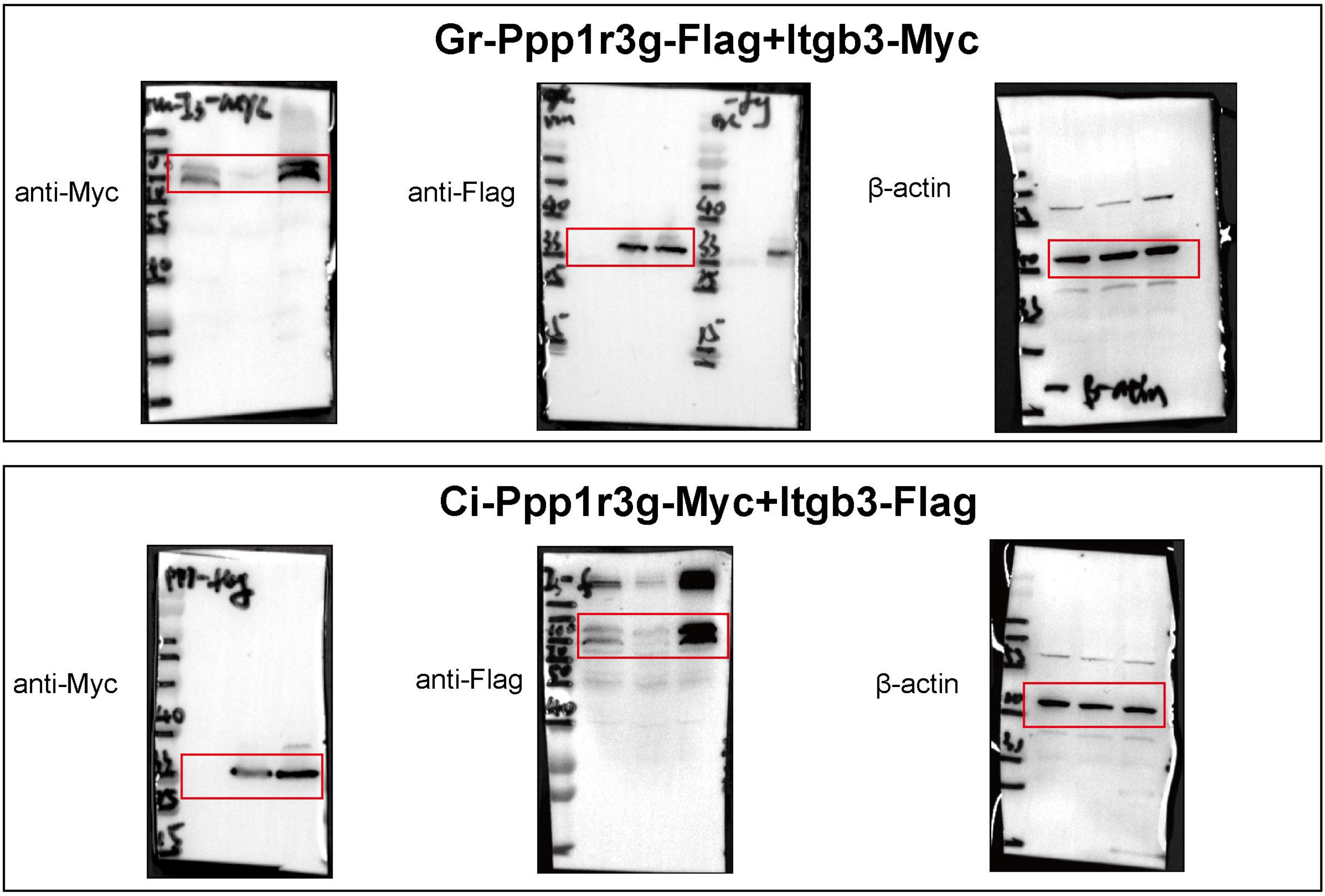
Gr-Ppp1r3g-Flag+Itgb3-Myc
anti-Myc
anti-Flag
β-actin
Ci-Ppp1r3g-Myc+Itgb3-Flag
anti-Myc
anti-Flag
β-actin

Fig 7D:

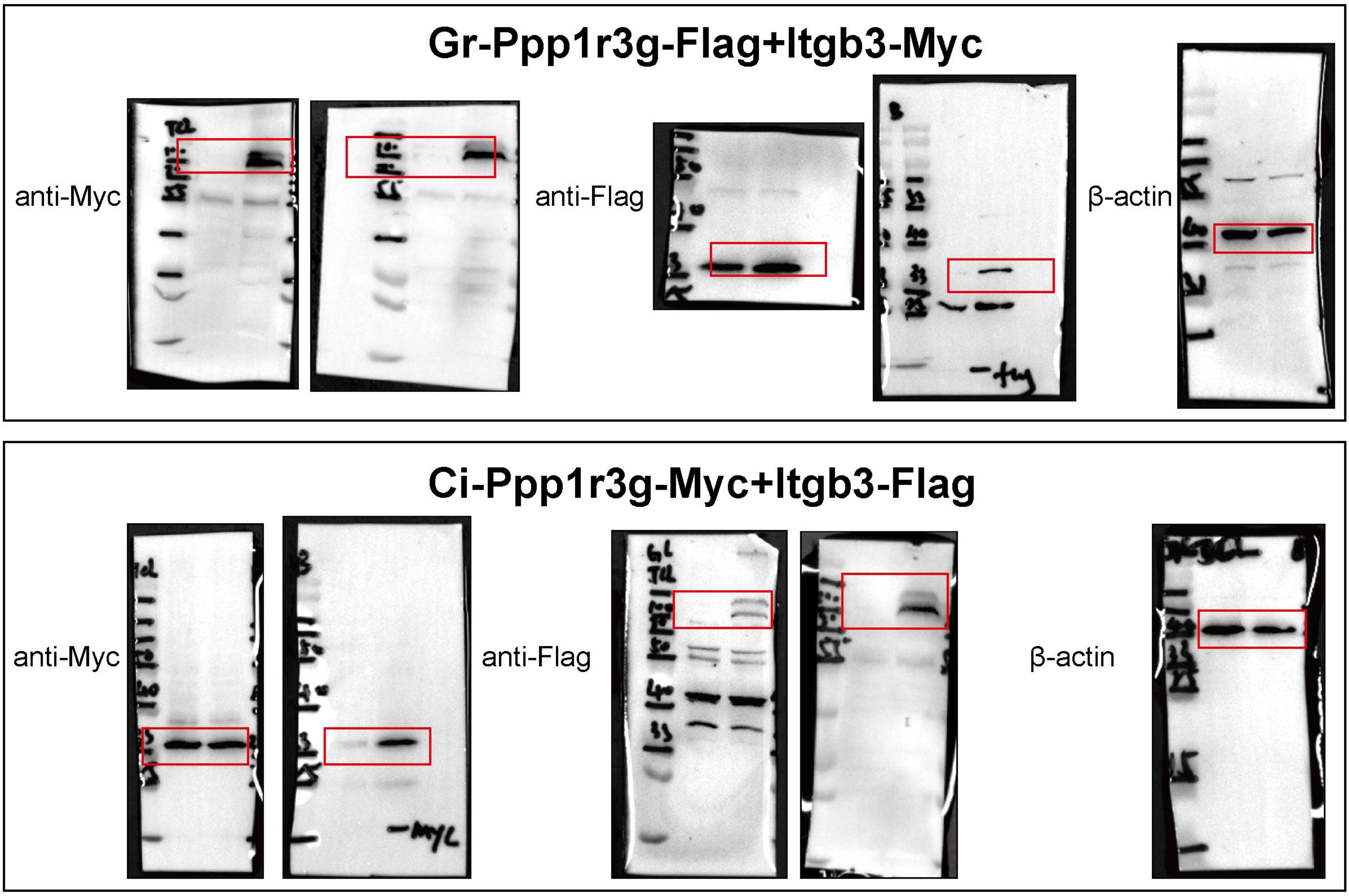
Gr-Ppp1r3g-Flag+Itgb3-Myc
anti-Myc
anti-Flag
β-actin
Ci-Ppp1r3g-Myc+Itgb3-Flag
anti-Myc
anti-Flag
β-actin

S1 A-B Fig:

Ci-Ppp1r3g-Flag+Tbk1-Myc

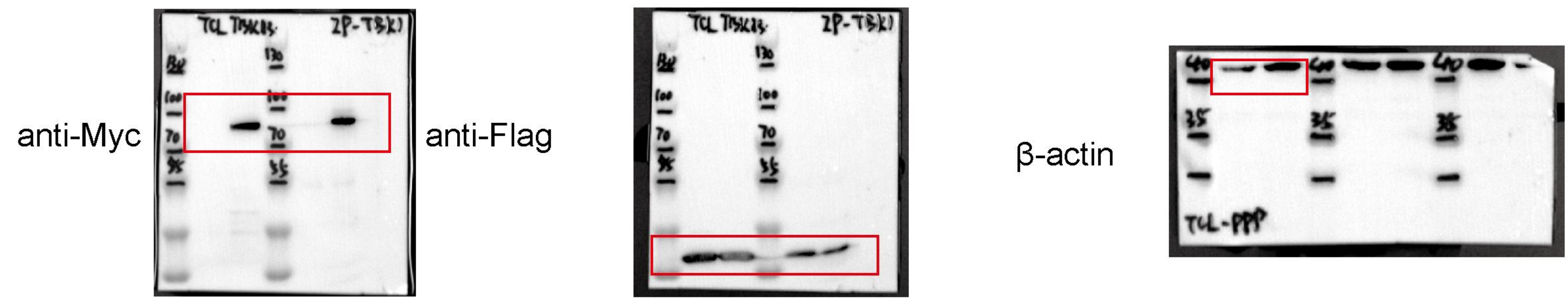

Ci-Ppp1r3g-Flag+Mvas-Myc

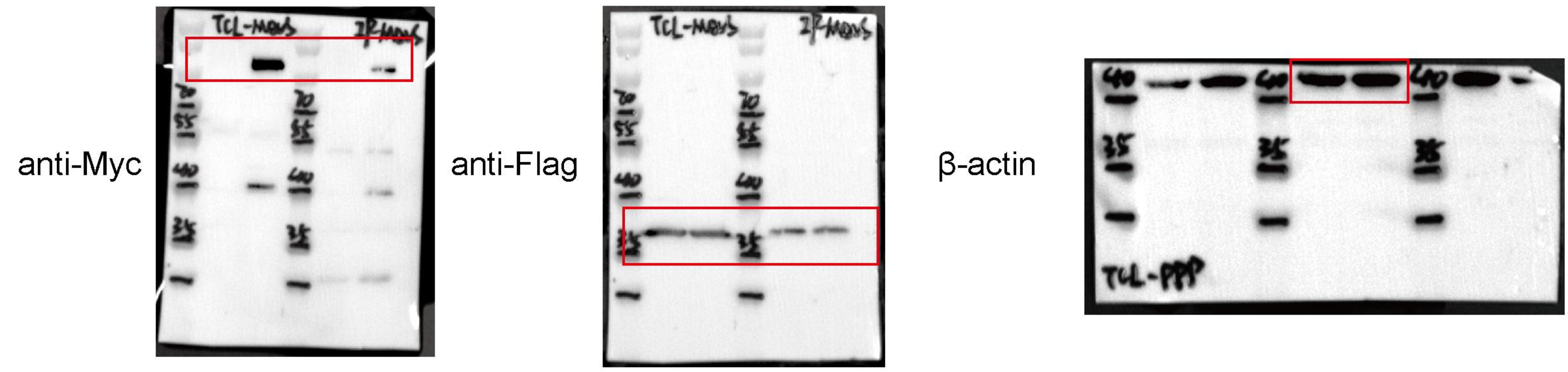

S1 C-D Fig :

Ci-Ppp1r3g-Flag+Traf3-Myc

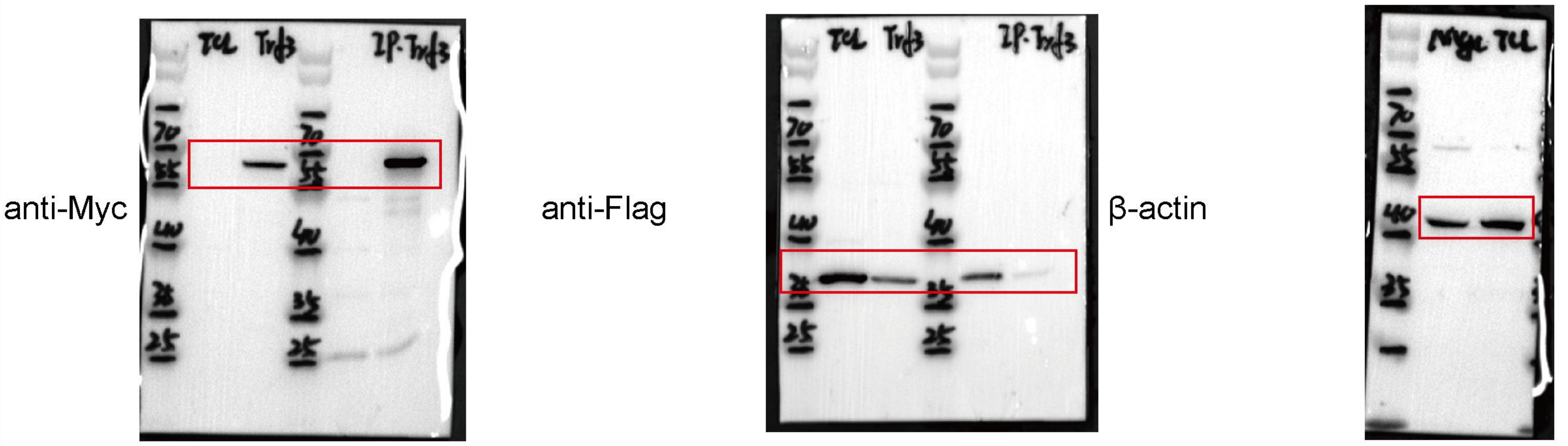

Ci-Ppp1r3g-Flag+Lgp2-GFP

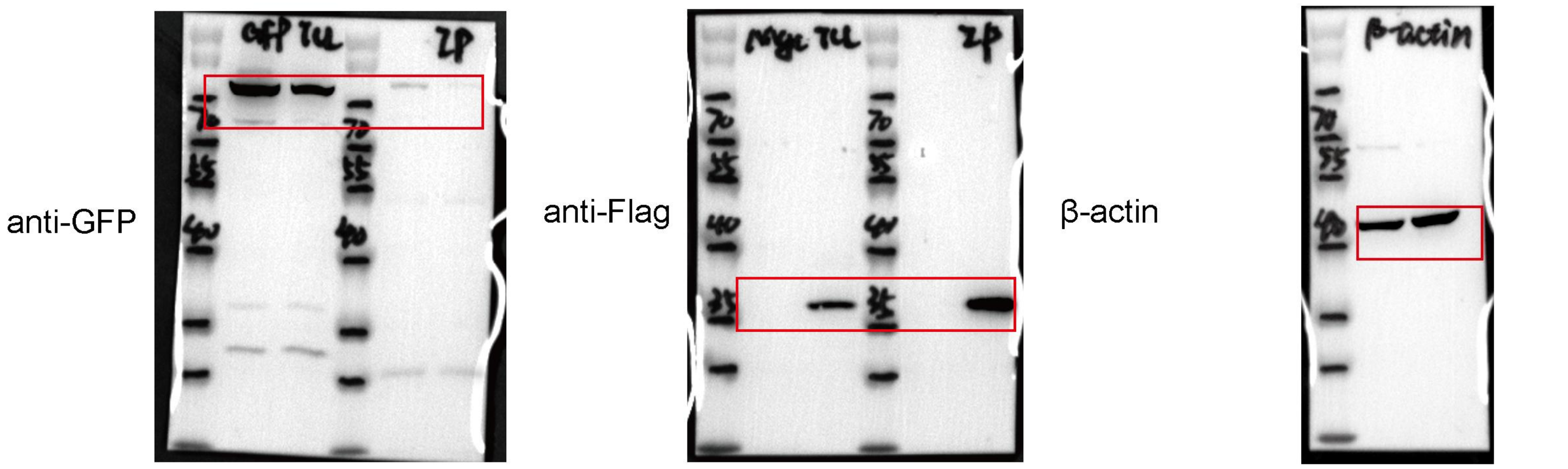

S1 E-G Fig

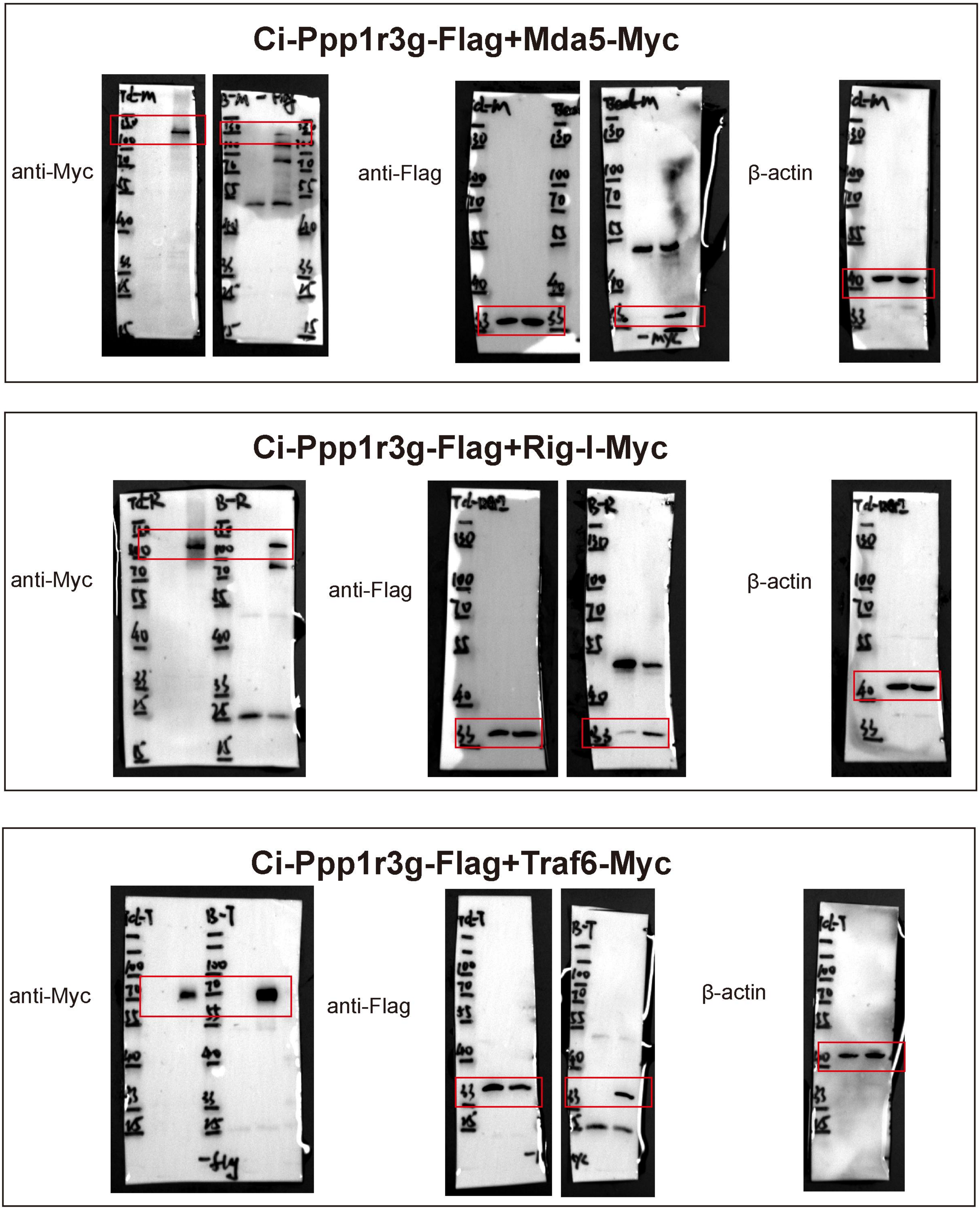
Ci-Ppp1r3g-Flag+Mda5-Myc
anti-Myc
anti-Flag
β-actin
Ci-Ppp1r3g-Flag+Rig-I-Myc
anti-Myc
anti-Flag
β-actin
Ci-Ppp1r3g-Flag+Traf6-Myc
anti-Myc
anti-Flag
β-actin

S3 A-B Fig：

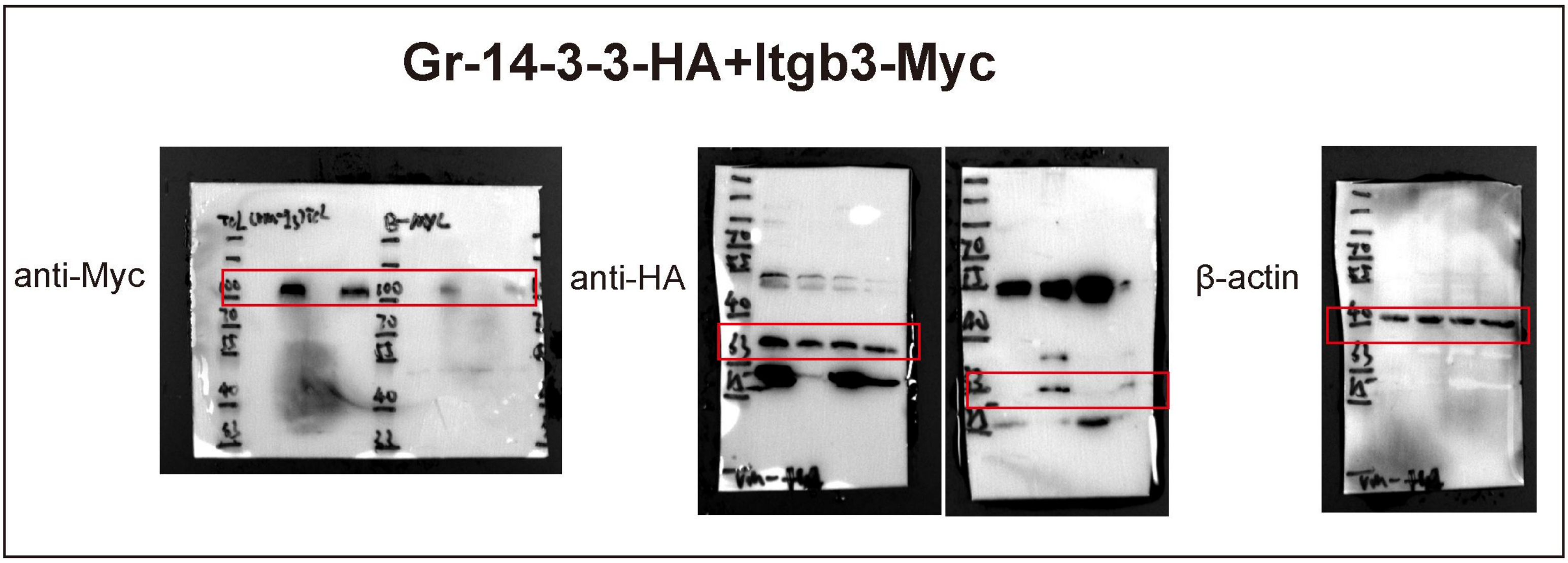
Gr-14-3-3-HA+Itgb3-Myc
anti-Myc
anti-HA
β-actin

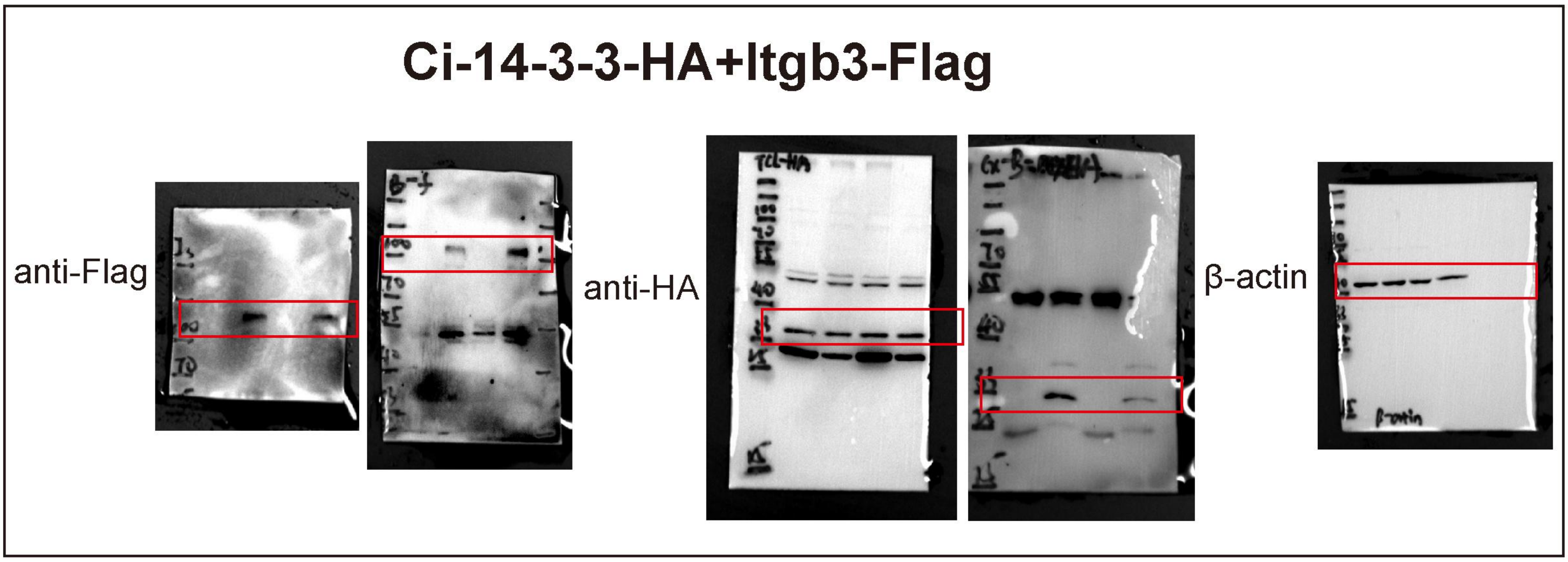
Ci-14-3-3-HA+Itgb3-Flag
anti-Flag
anti-HA
β-actin

S4 A Fig :

Gr-Ppp1r3g-Flag+Itgb3-Myc

anti-Myc

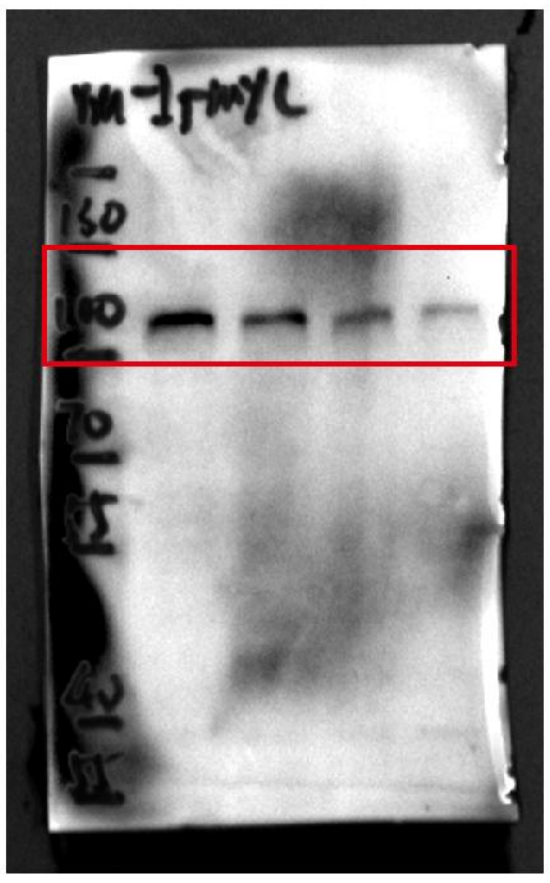

anti-Flag

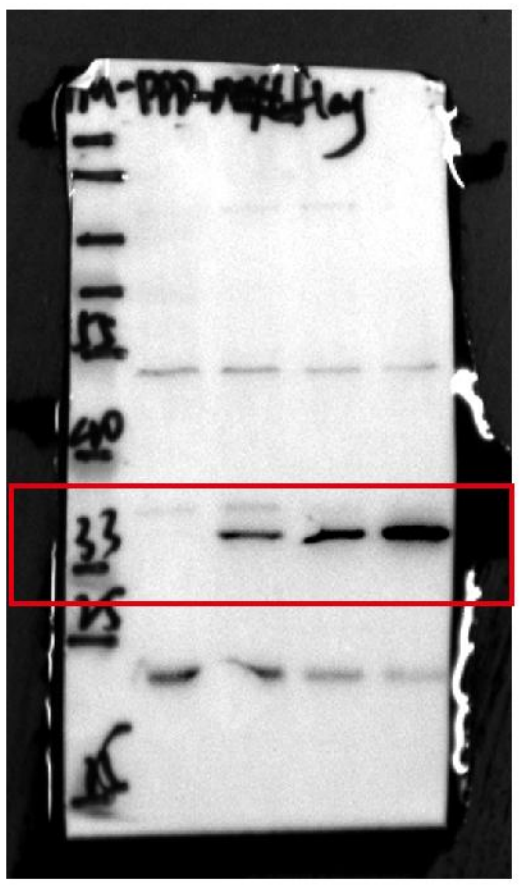

β-actin

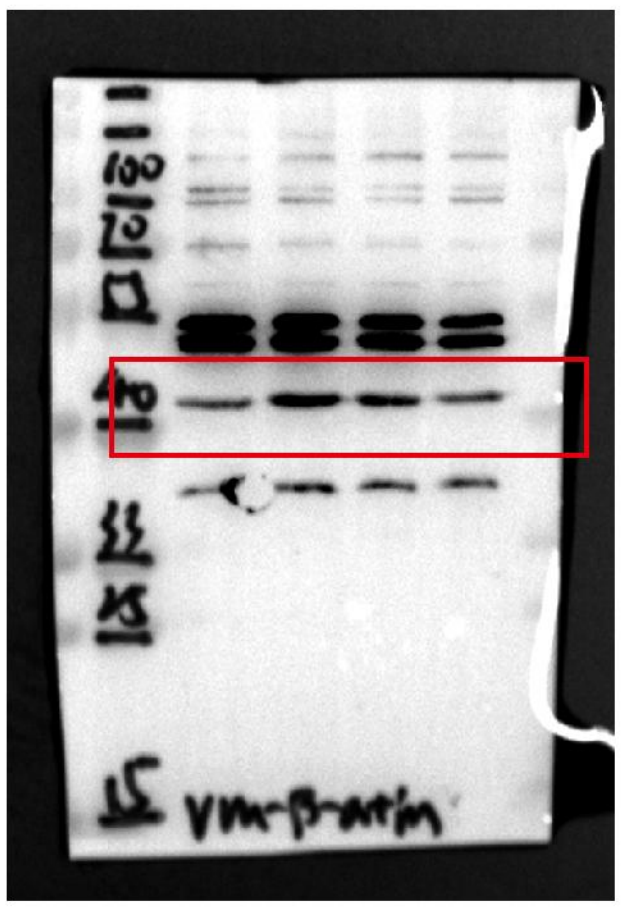

Gr-Ppp1r3g-Flag+Itgb3-Myc

anti-Myc

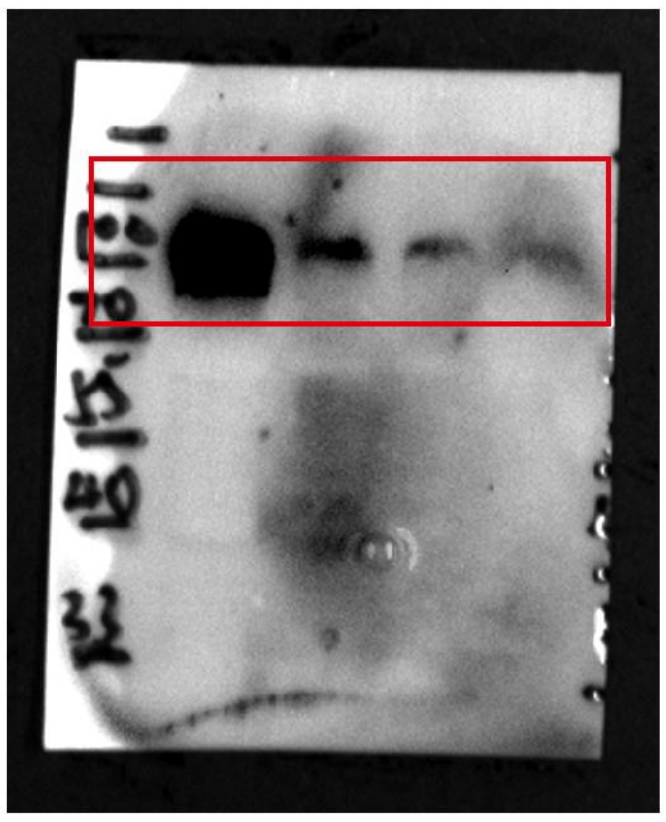

anti-Flag

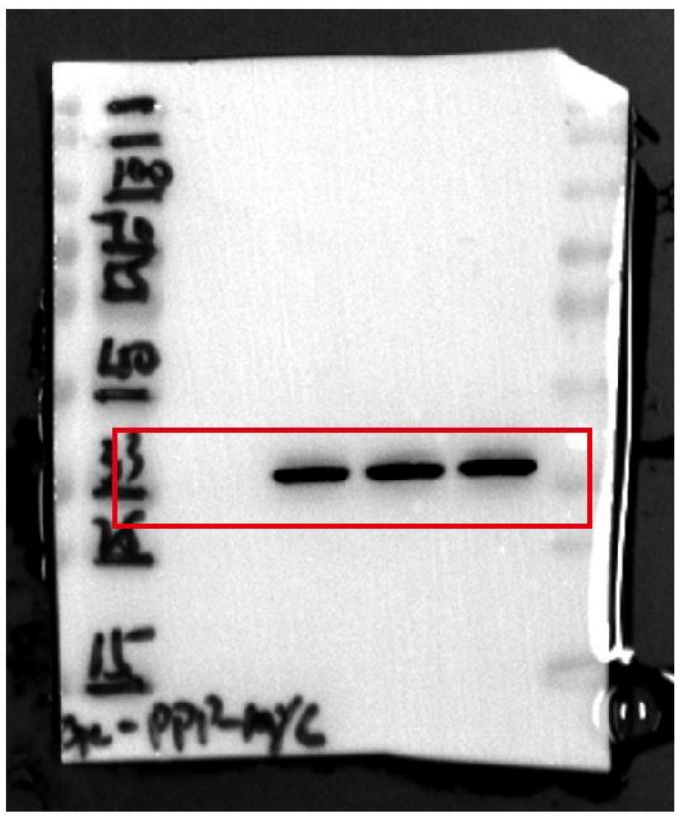

β-actin

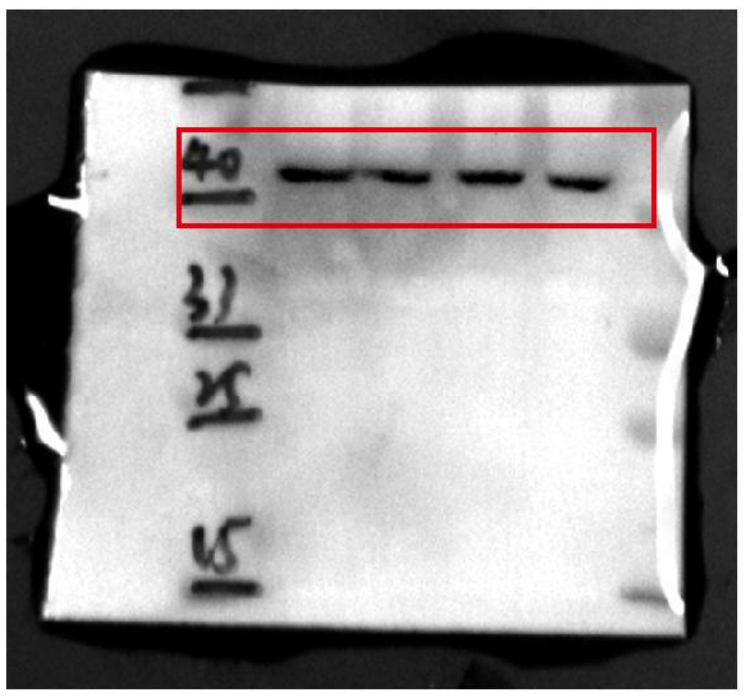

Gr-Ppp1r3g-Flag+Itgb3-Myc

anti-Myc

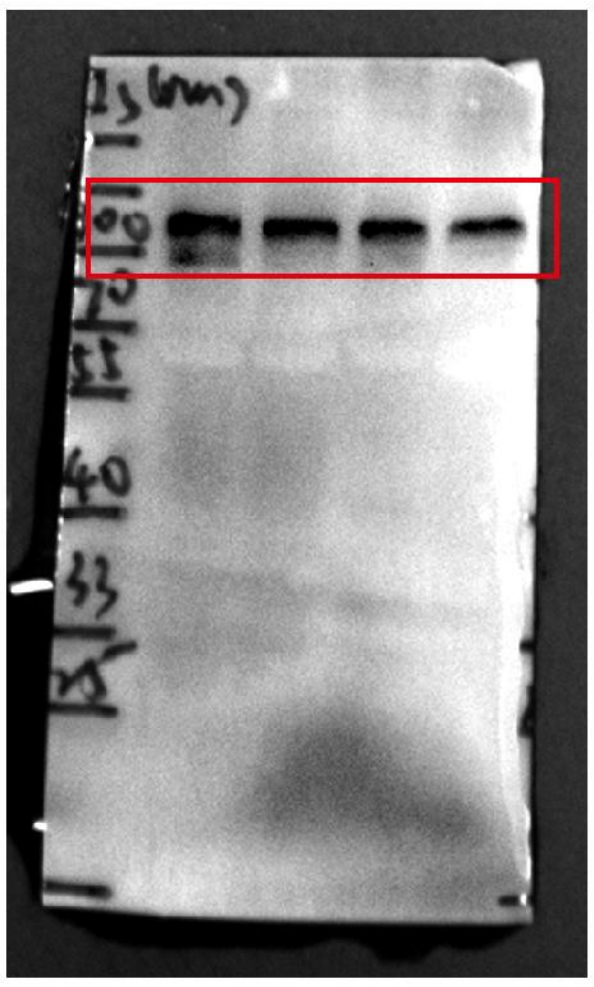

anti-Flag

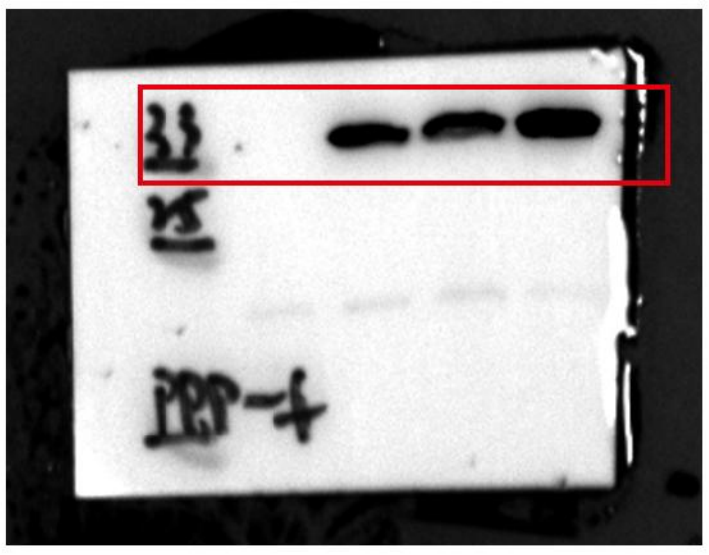

β-actin

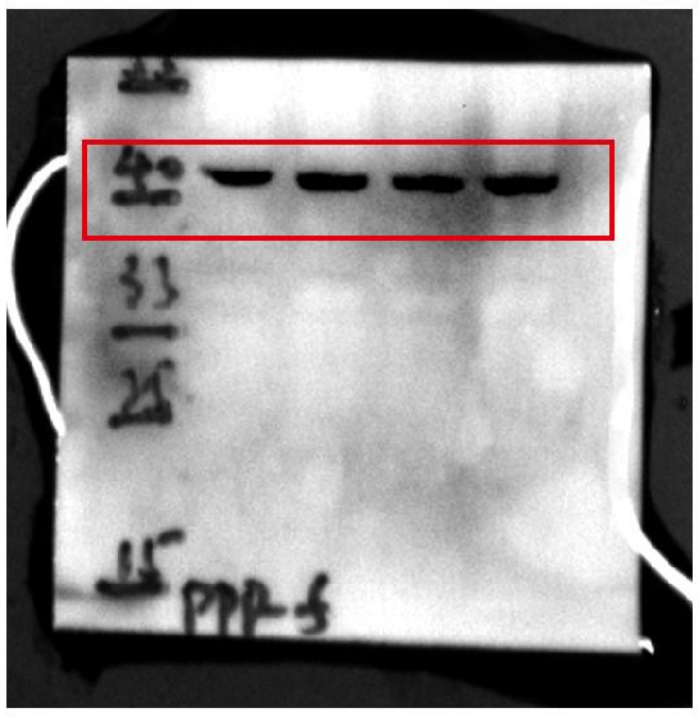

S4 B Fig :

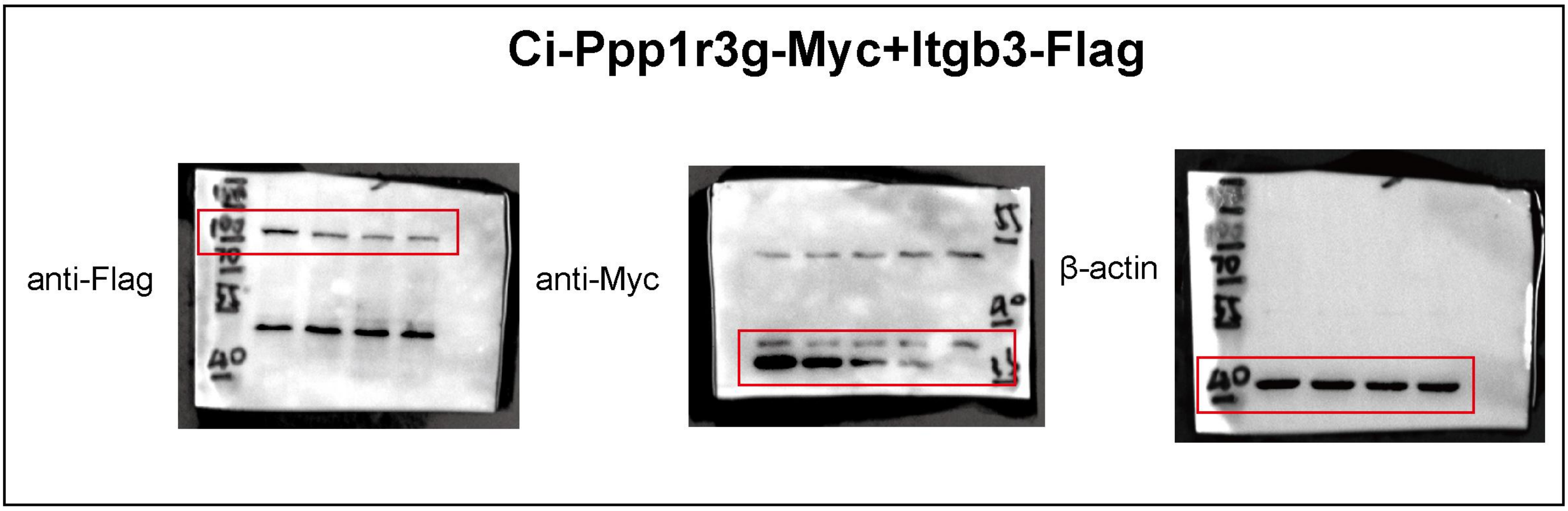
Ci-Ppp1r3g-Myc+Itgb3-Flag
anti-Flag
anti-Myc
β-actin

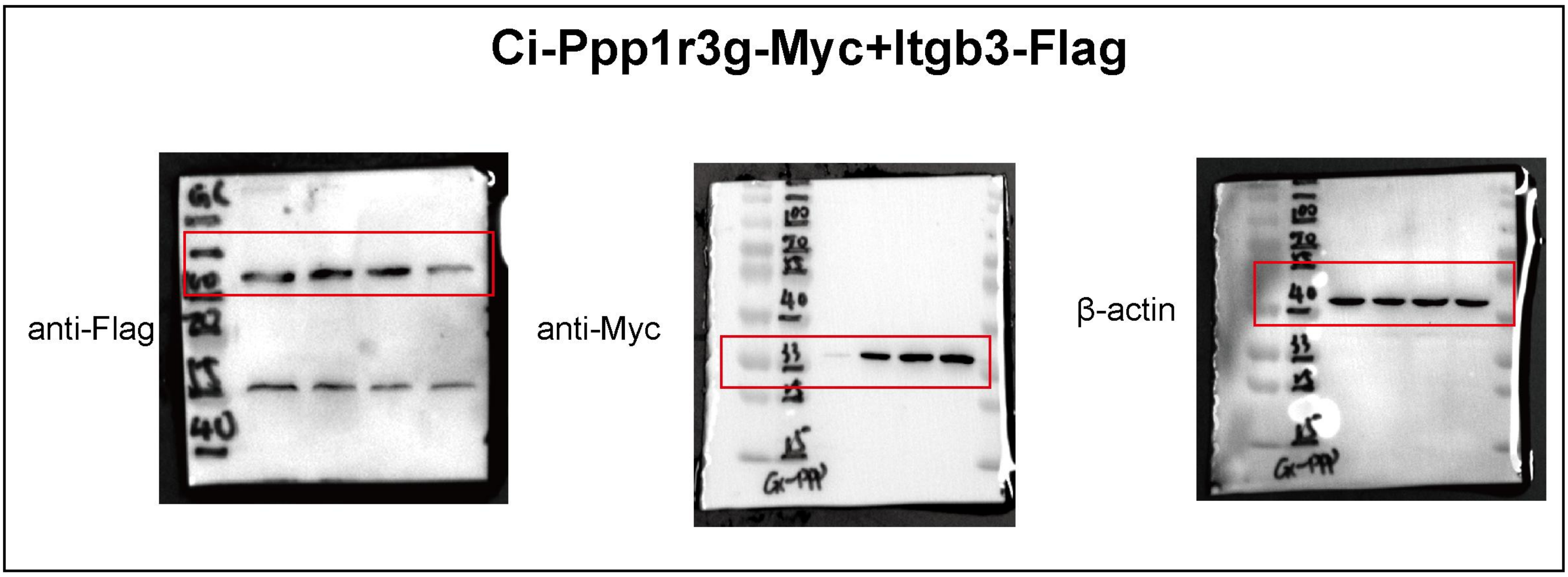
Ci-Ppp1r3g-Myc+Itgb3-Flag
anti-Flag
anti-Myc
β-actin

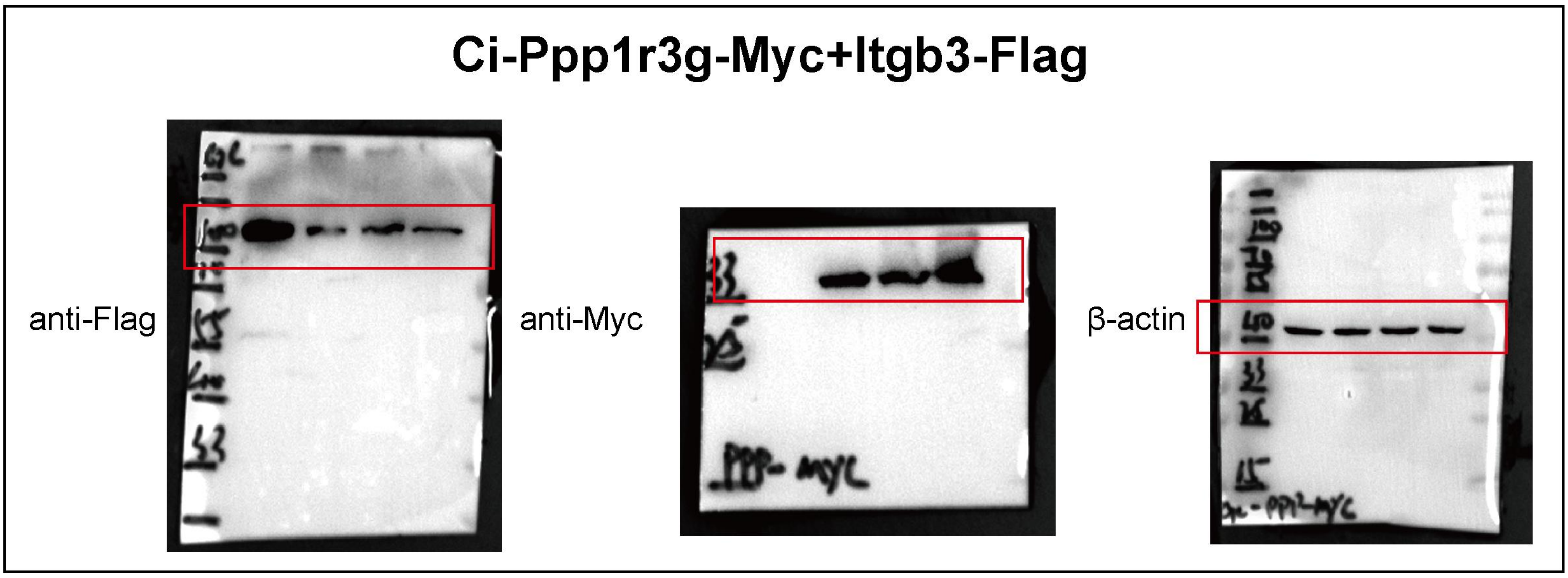
Ci-Ppp1r3g-Myc+Itgb3-Flag
anti-Flag
anti-Myc
β-actin

S5 A-B Fig :

Gr-Ppp1r3g-Flag+Itgb3-Myc +14-3-3ζ-HA

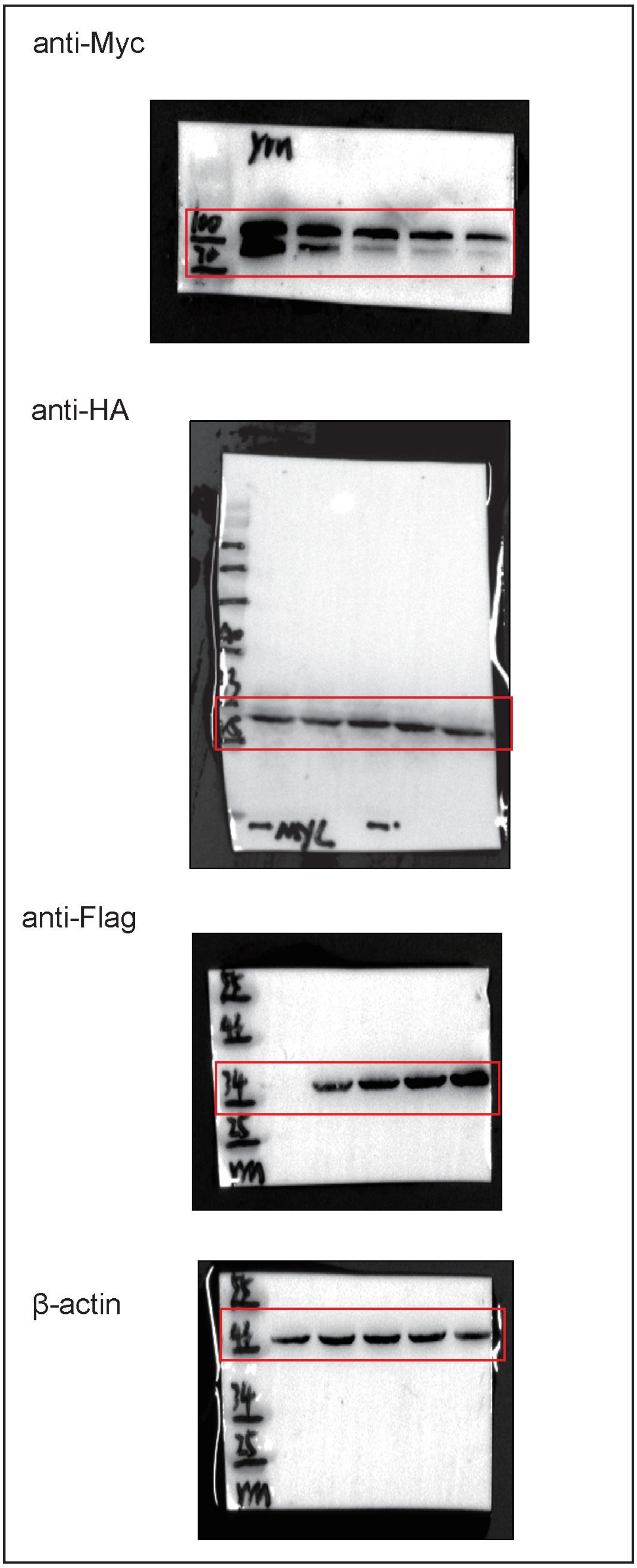

Ci-Ppp1r3g-Myc+Itgb3-Flag +14-3-3ζ-HA

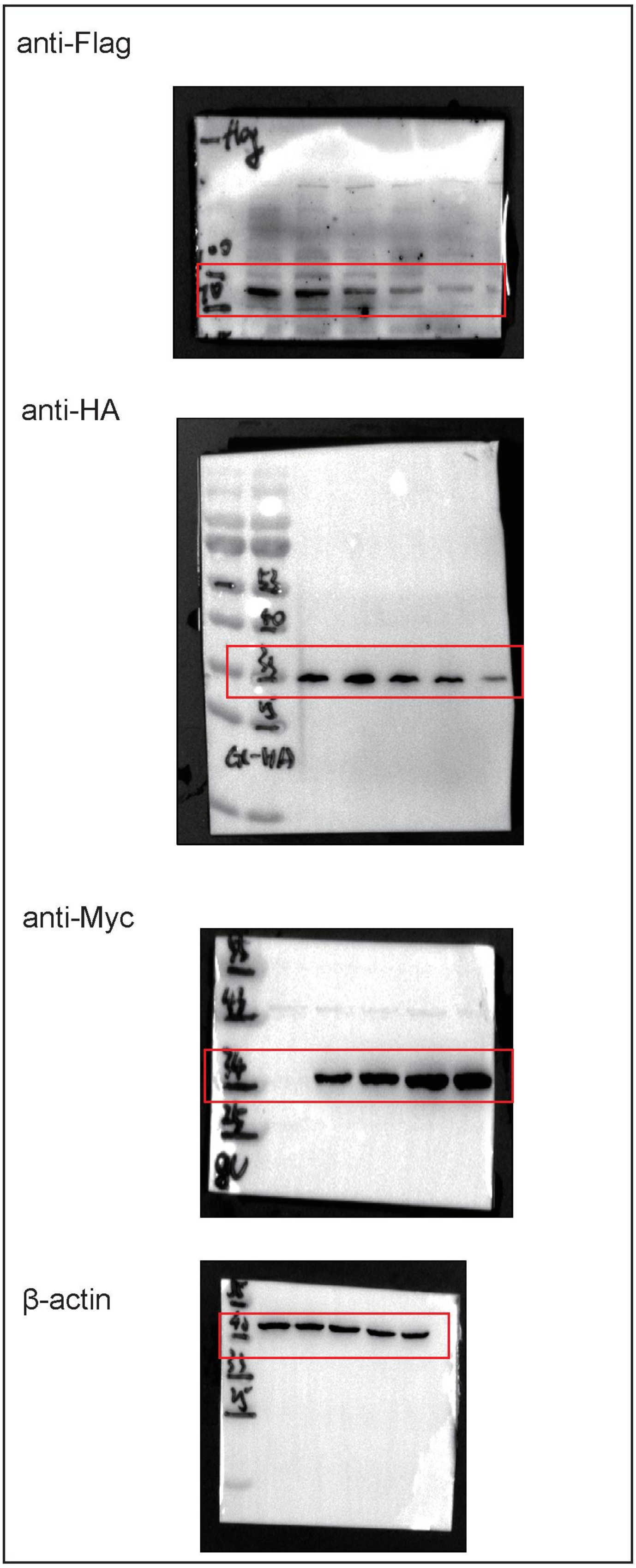

S6 A-B Fig：

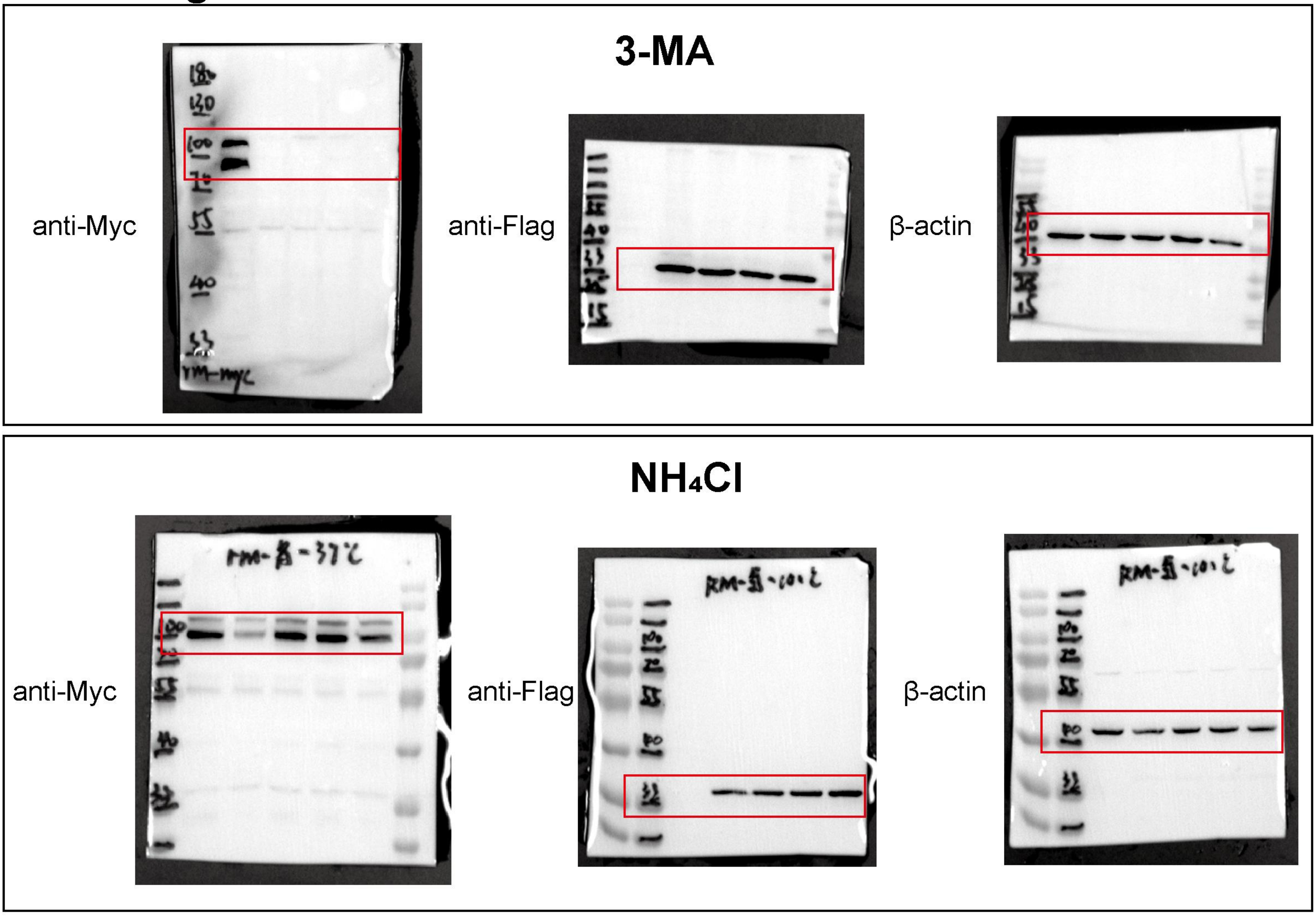

S6 C-D Fig：

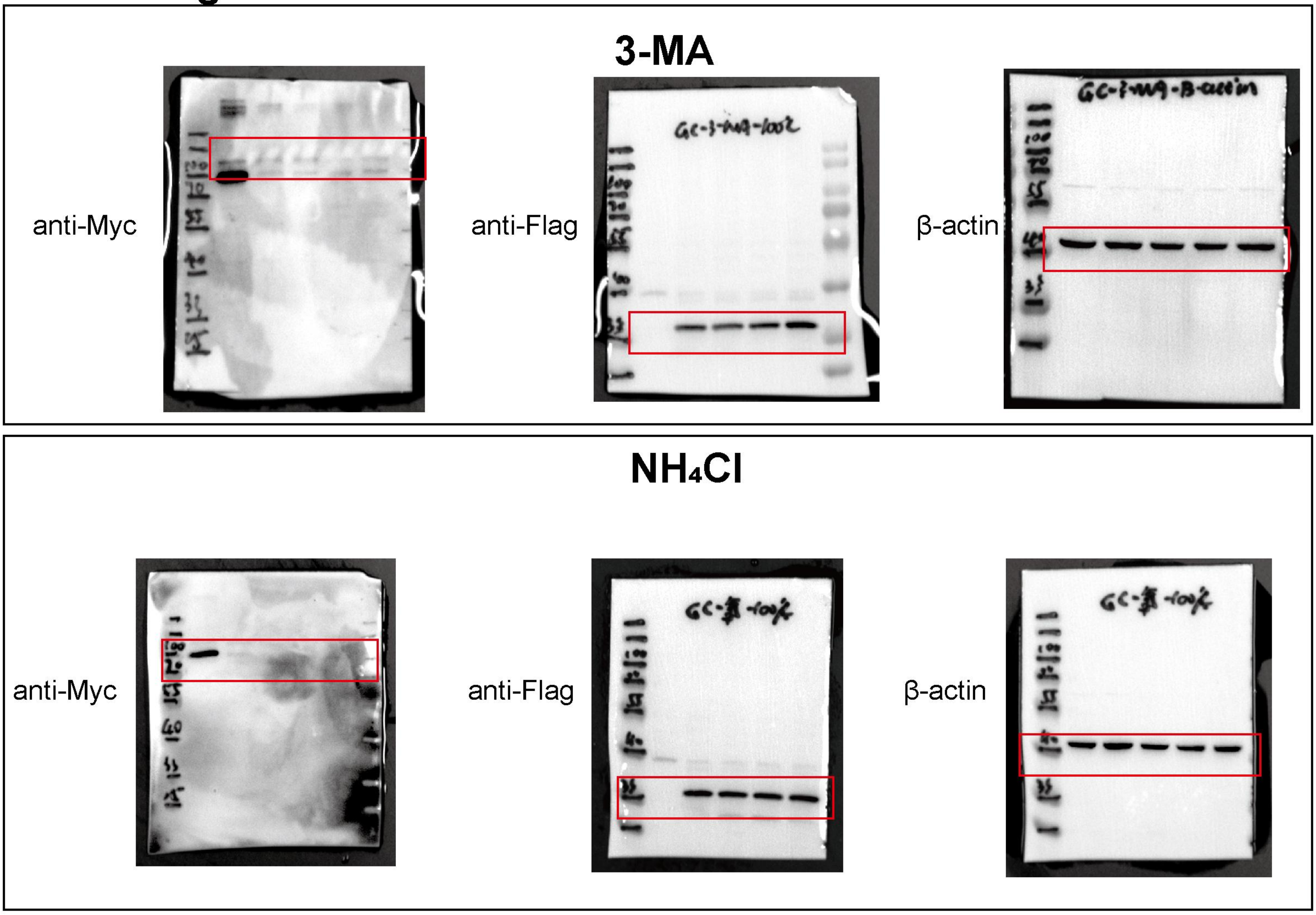

S7 A-B Fig：

Ci-Ppp1r3g-Flag+Ci-Itgb3-Myc+His-Ub

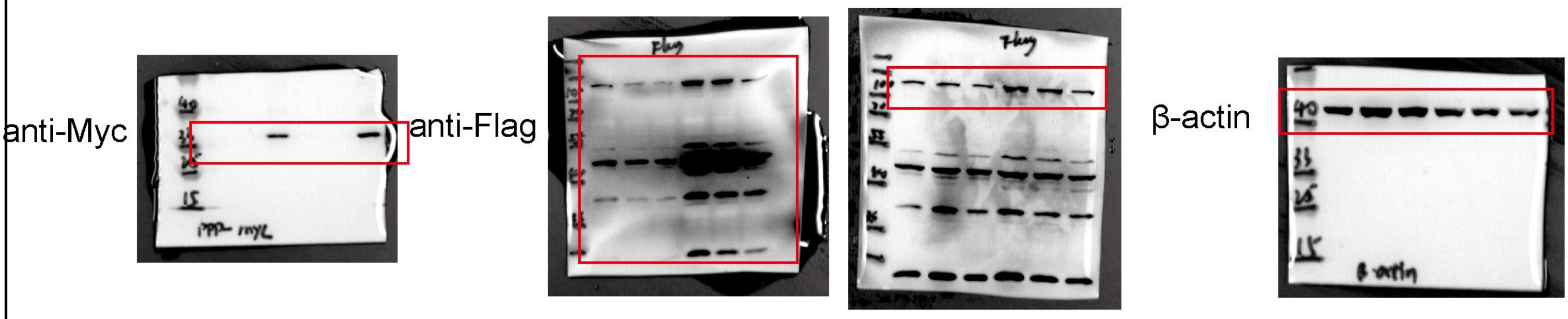

Gr-Ppp1r3g-Myc+Gr-Itgb3-Flag+His-Ub

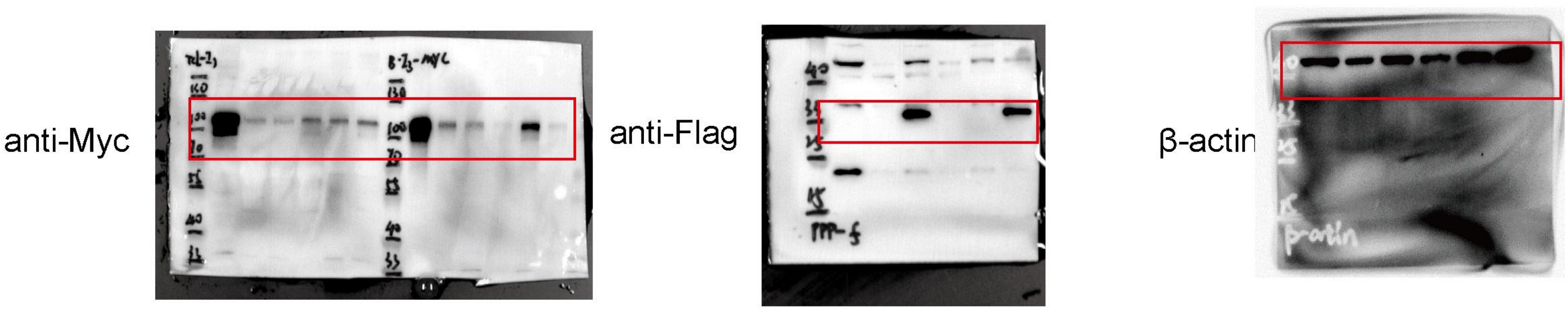

Supplement: Supplementary file 1 [file ppat.1014590.s012.pdf]
