# Supplementary material for: Grass carp reovirus activates fish Ppp1r3g to suppress innate immunity and exacerbate hemorrhage via the 14-3-3ζ/Itgb3 signaling axis
Source: PLoS Pathog. 2026 Sep 18;22(9):e1014590. doi: 10.1371/journal.ppat.1014590 (PMC13588375; doi:10.1371/journal.ppat.1014590)
Supplement: Supplementary file 2 [file ppat.1014590.s013.pdf]

Fig 2C

ppp1r3g$^{-/-}$

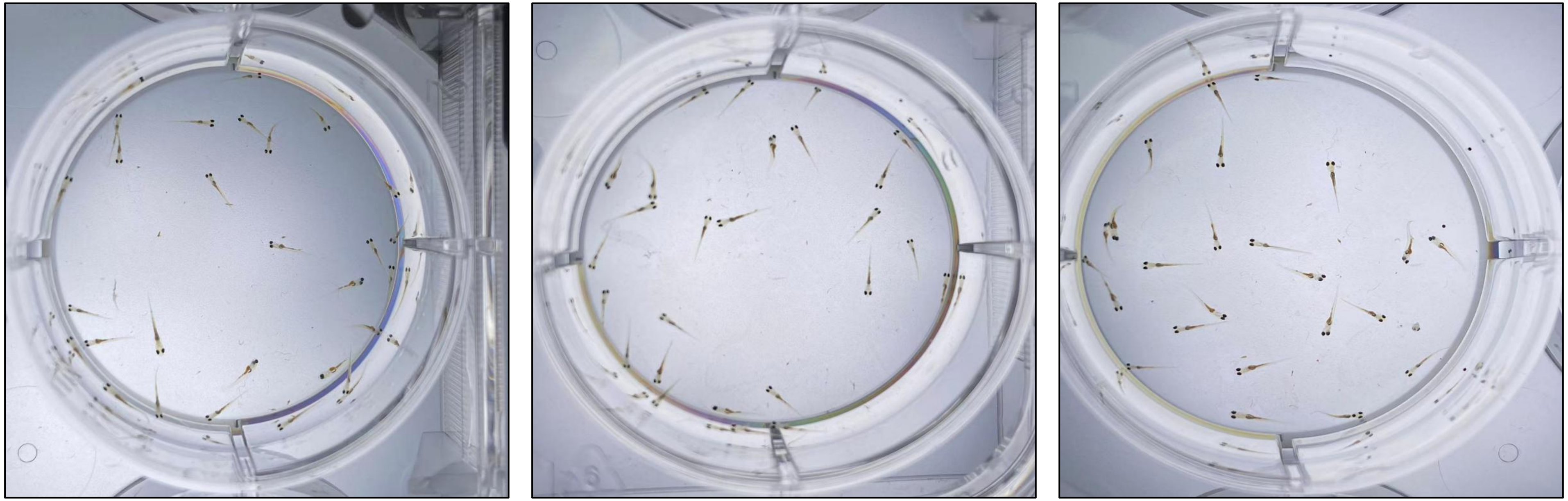

ppp1r3g$^{+/+}$

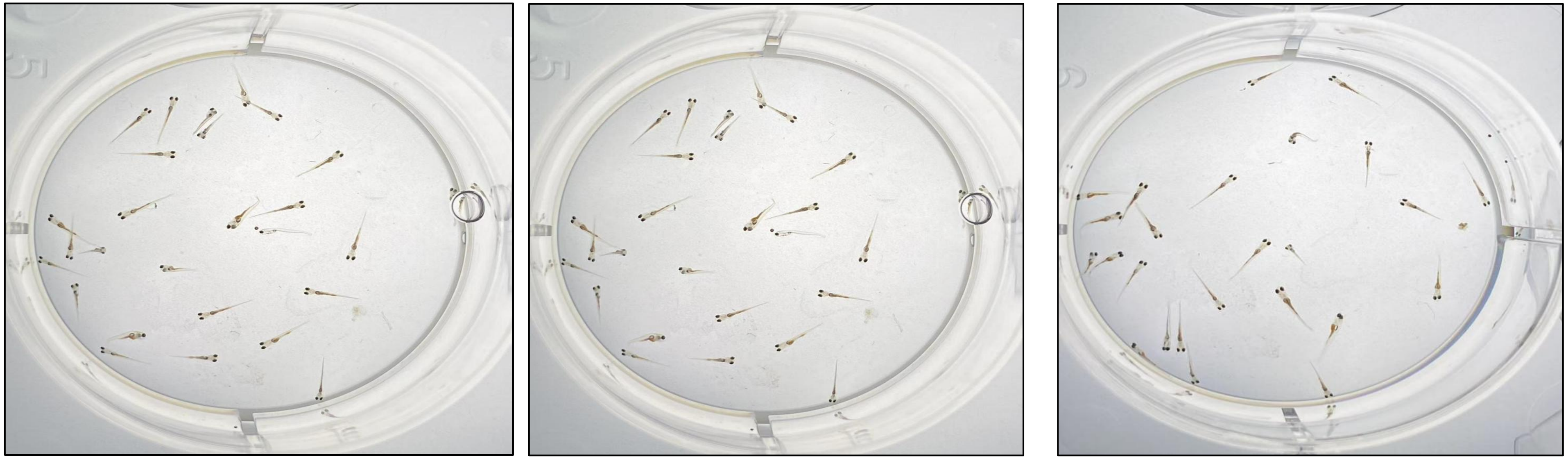

Fig 2C

ppp1r3g$^{-/-}$ +GCRV

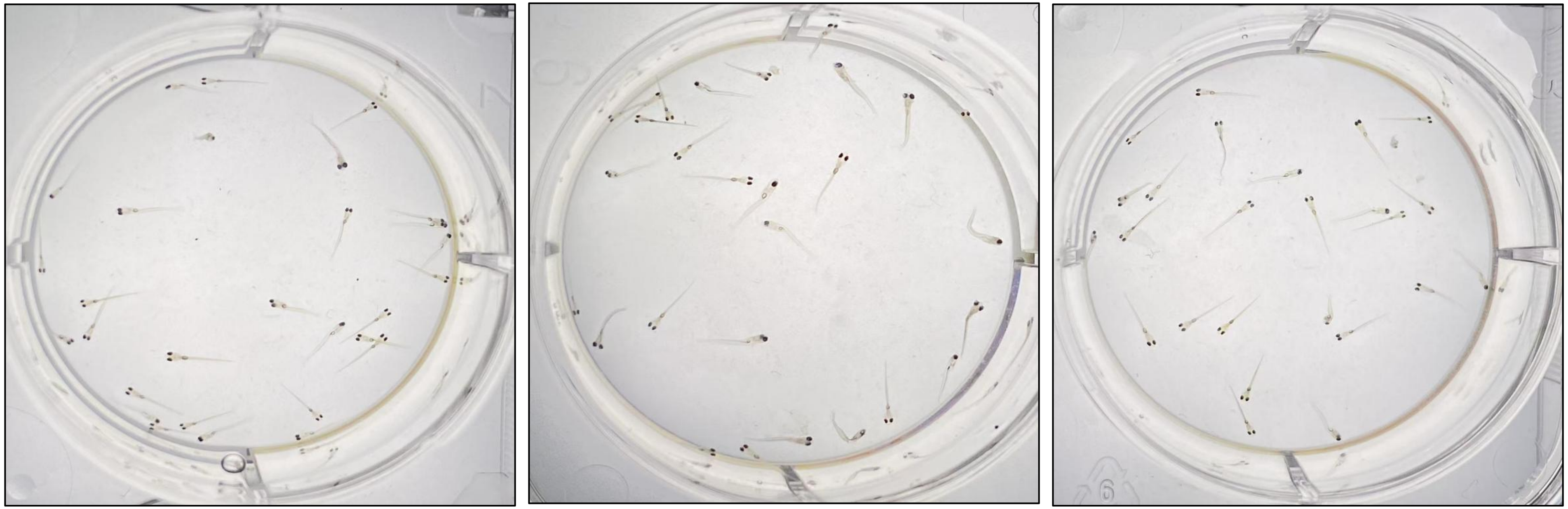

ppp1r3g$^{+/+}$ +GCRV

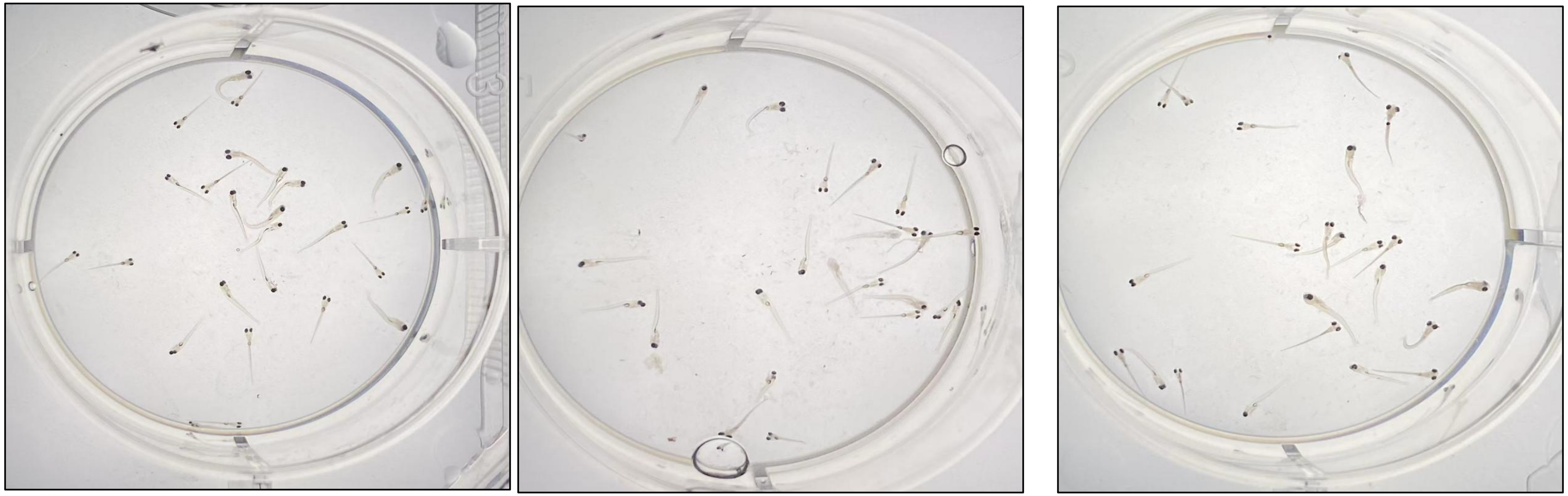

Fig 2D

+GCRV

ppp1r3g$^{+/+}$

ppp1r3g$^{-/-}$

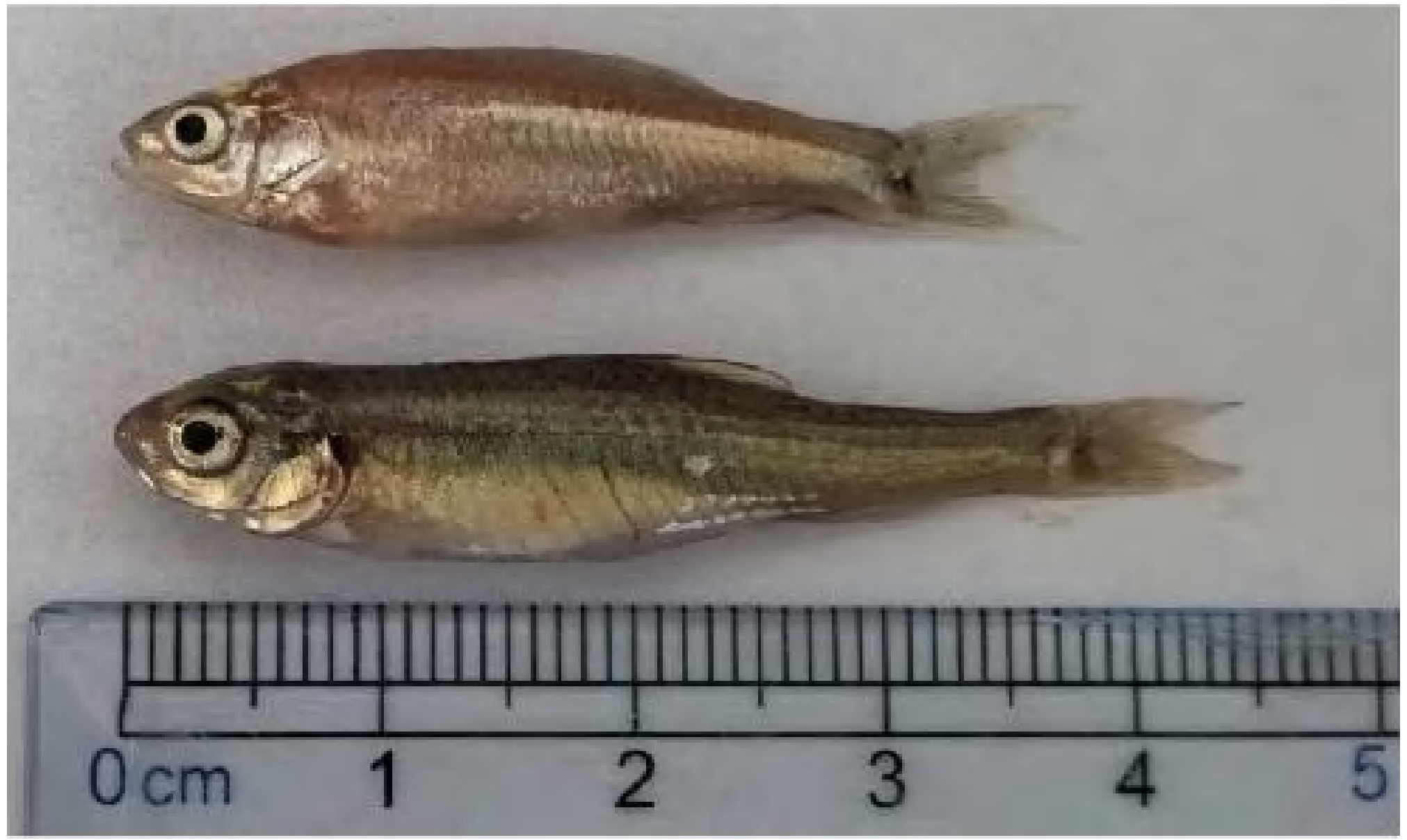

ppp1r3g$^{+/+}$

ppp1r3g$^{-/-}$

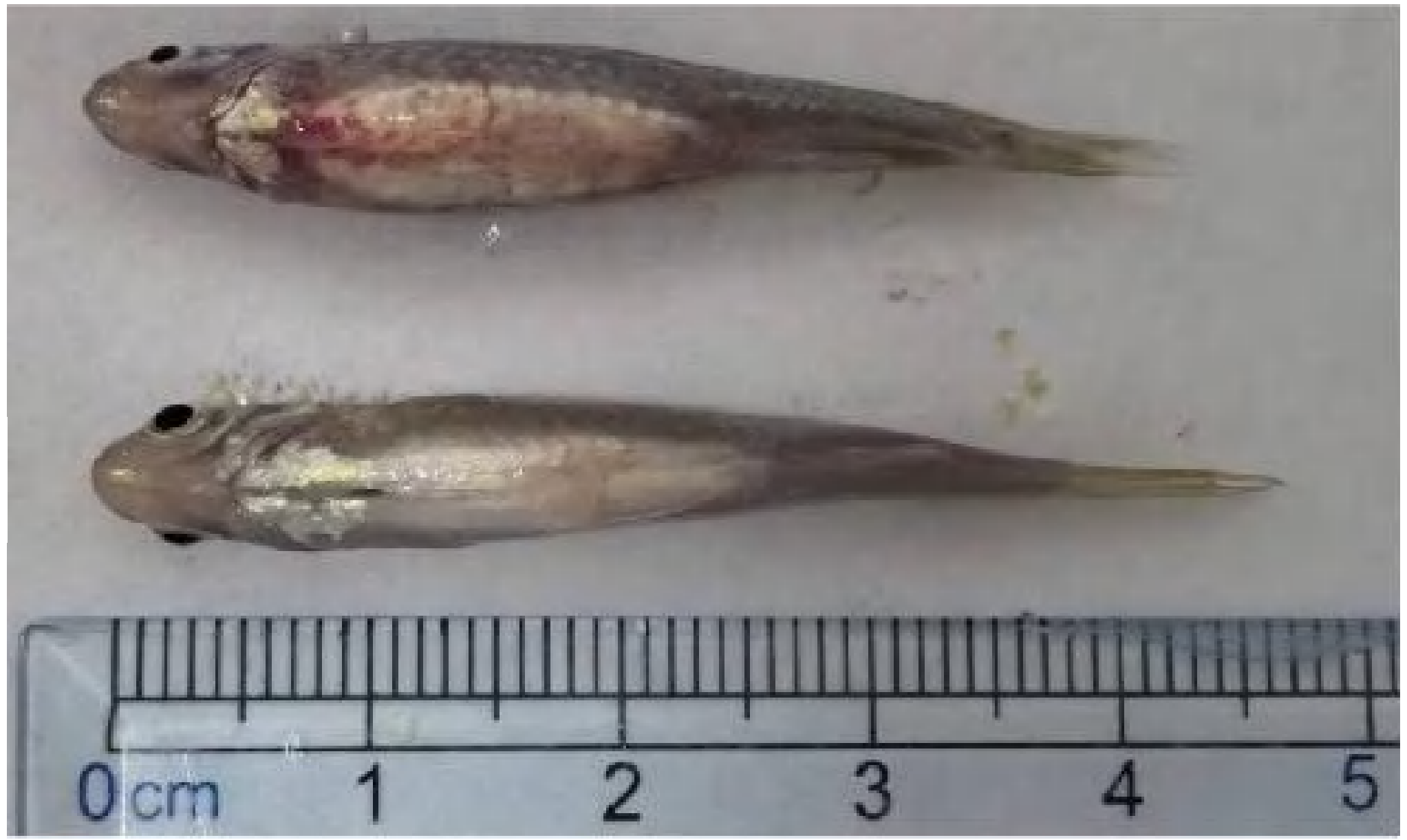

Fig 2E

-GCRV

WT

ppp1r3g-chimera

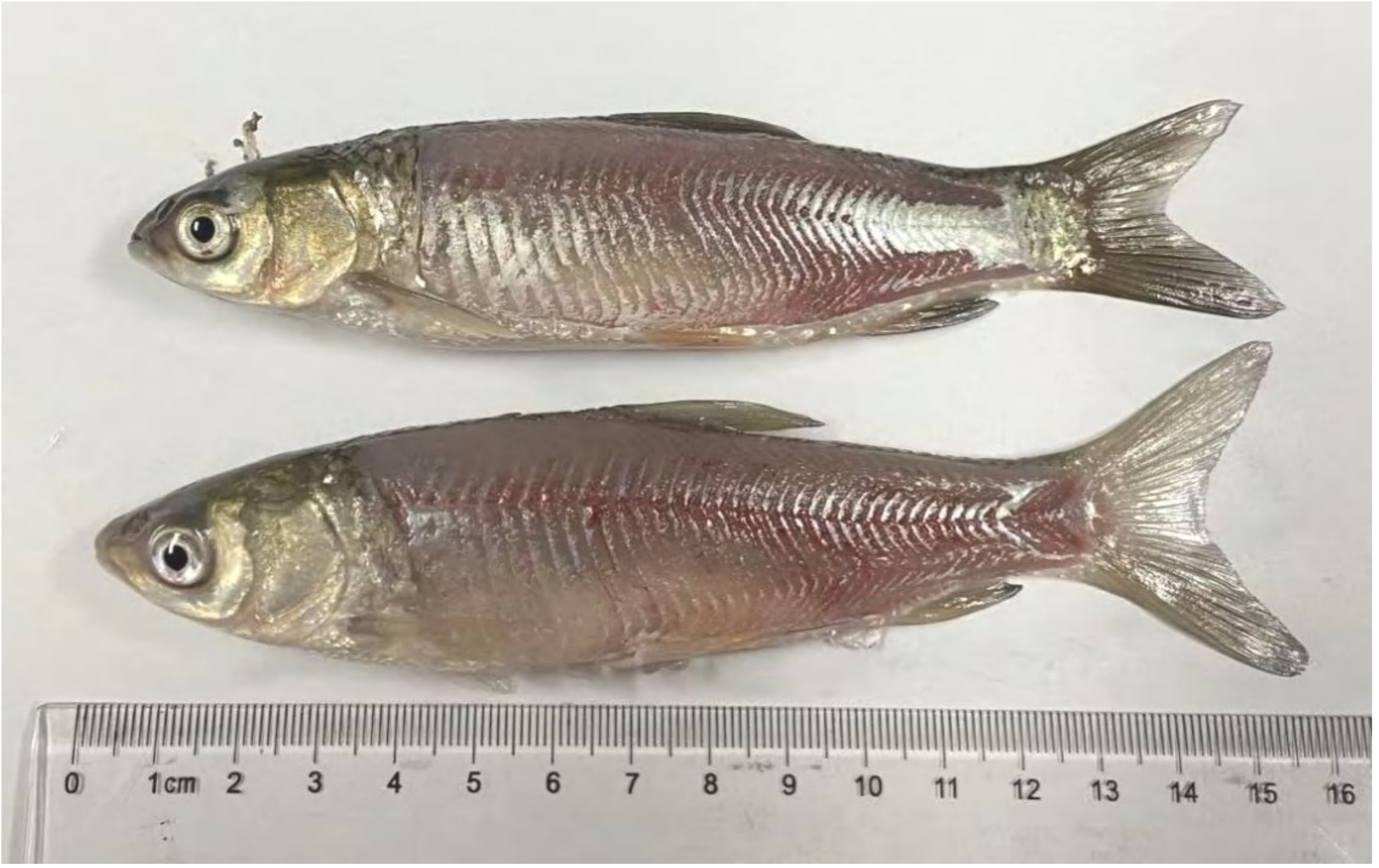

+GCRV

WT

ppp1r3g-chimera

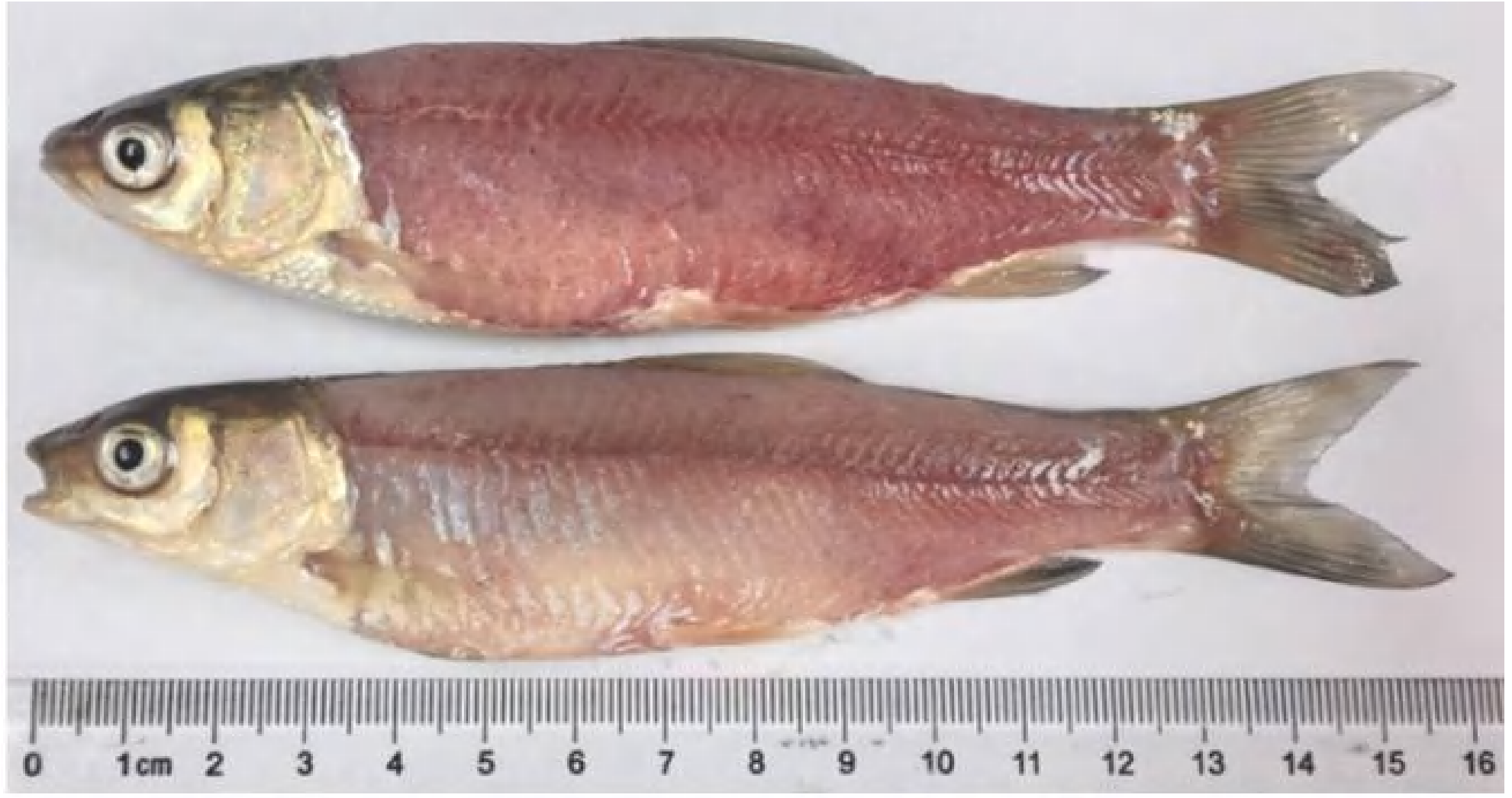

Fig 3A

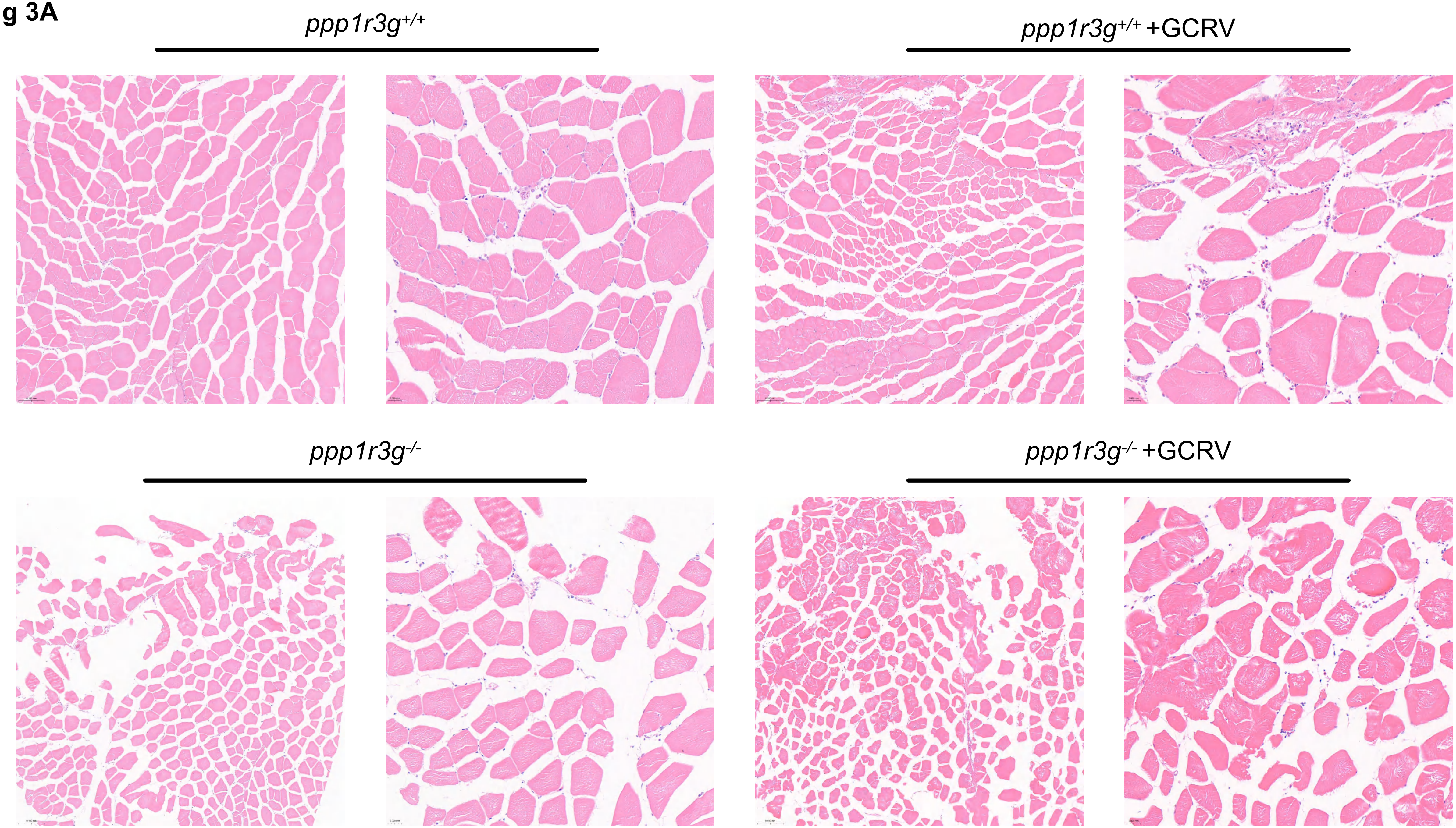

Fig 3B

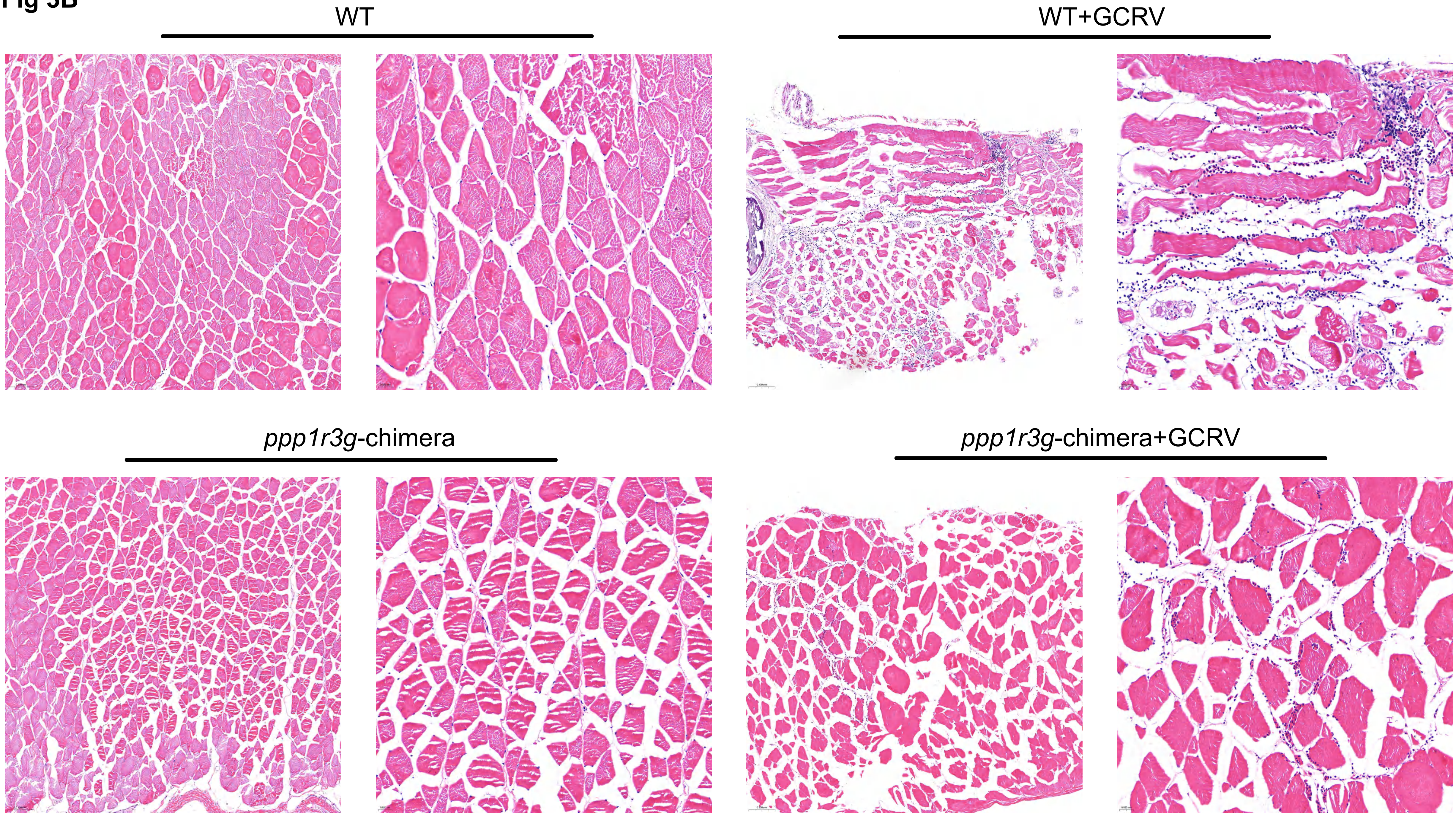

Fig 4D

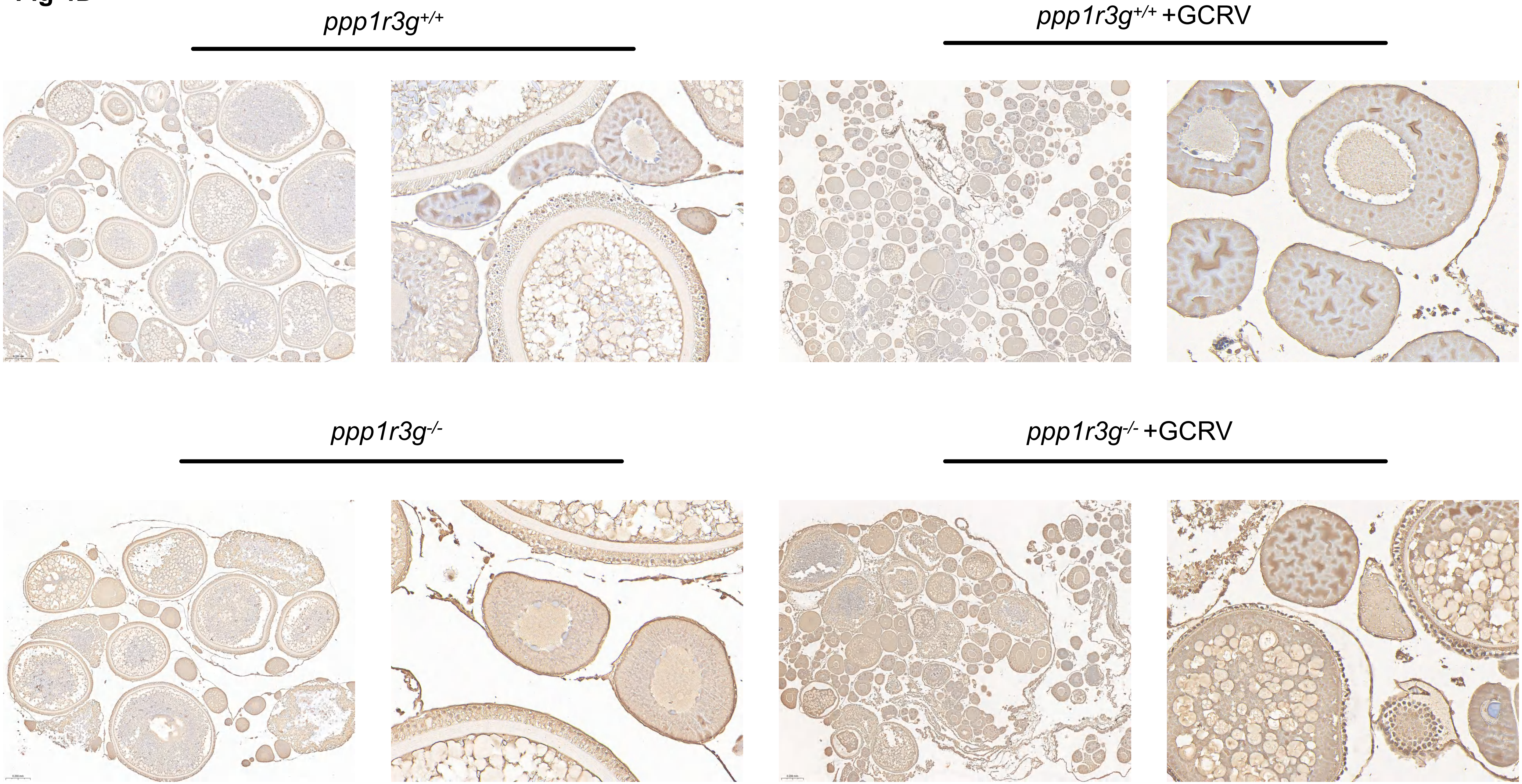

Fig 4E

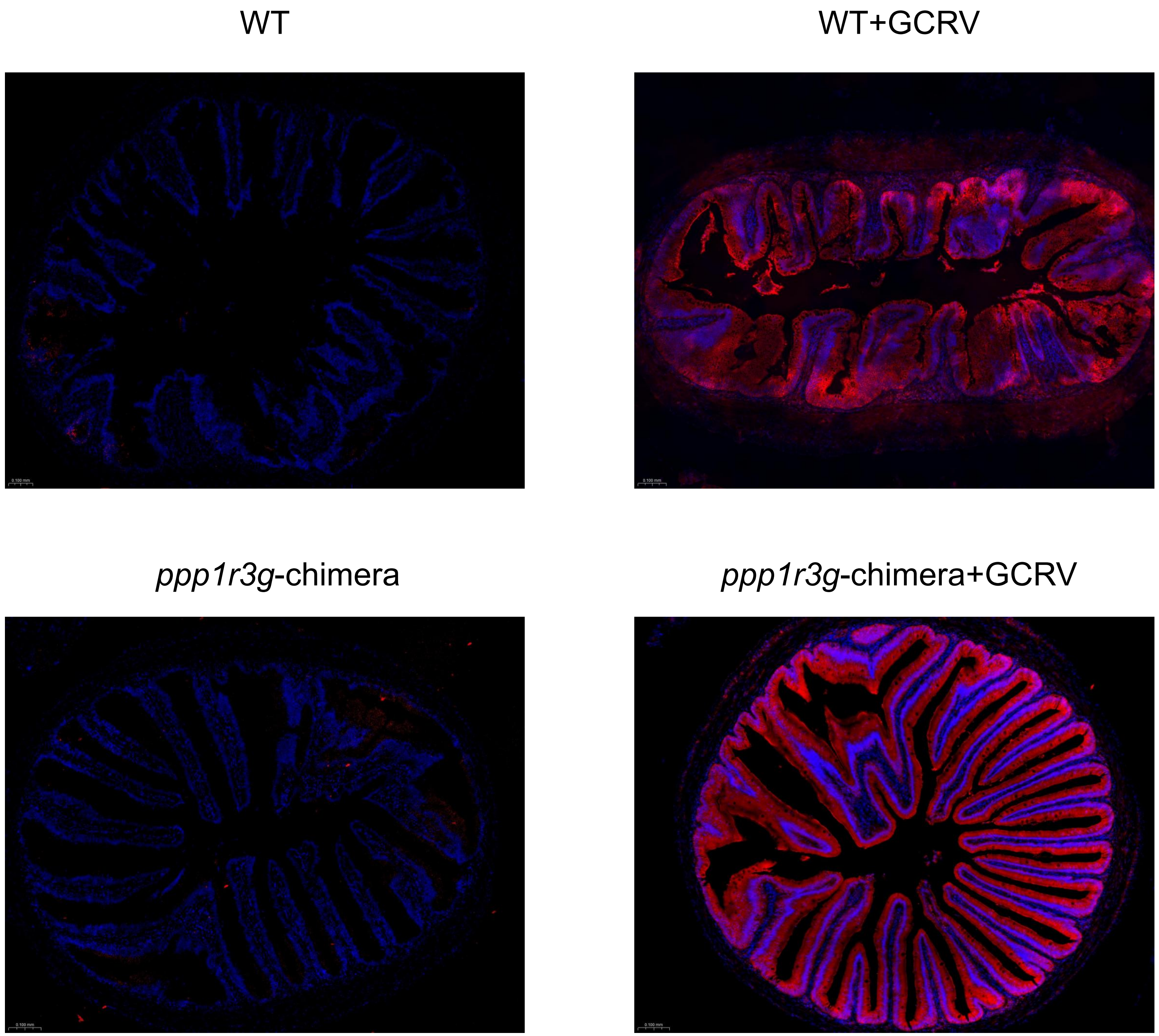

Fig 5C

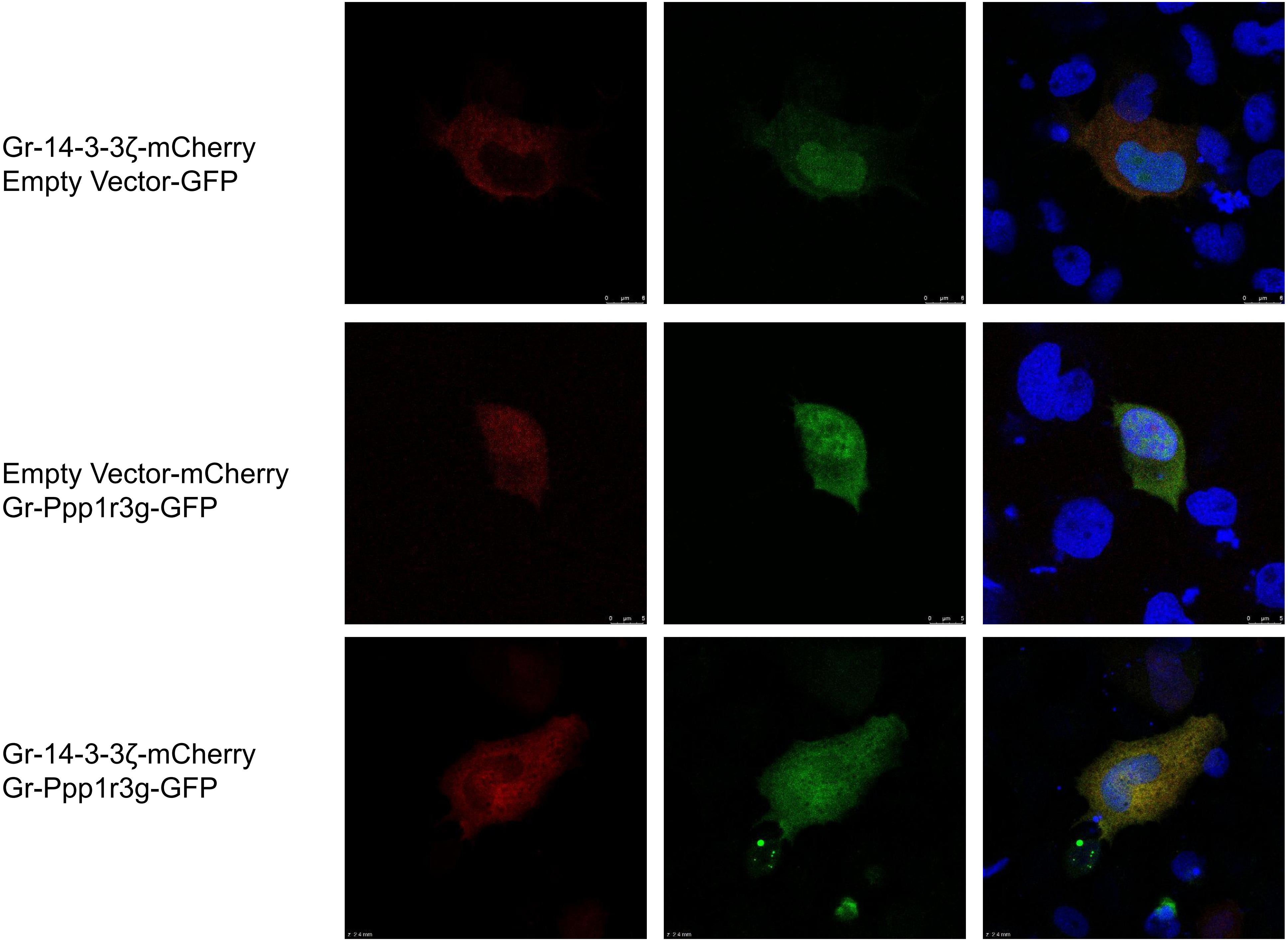
Gr-14-3-3ζ-mCherry
Empty Vector-GFP
Empty Vector-mCherry
Gr-Ppp1r3g-GFP
Gr-14-3-3ζ-mCherry
Gr-Ppp1r3g-GFP

Fig 5C

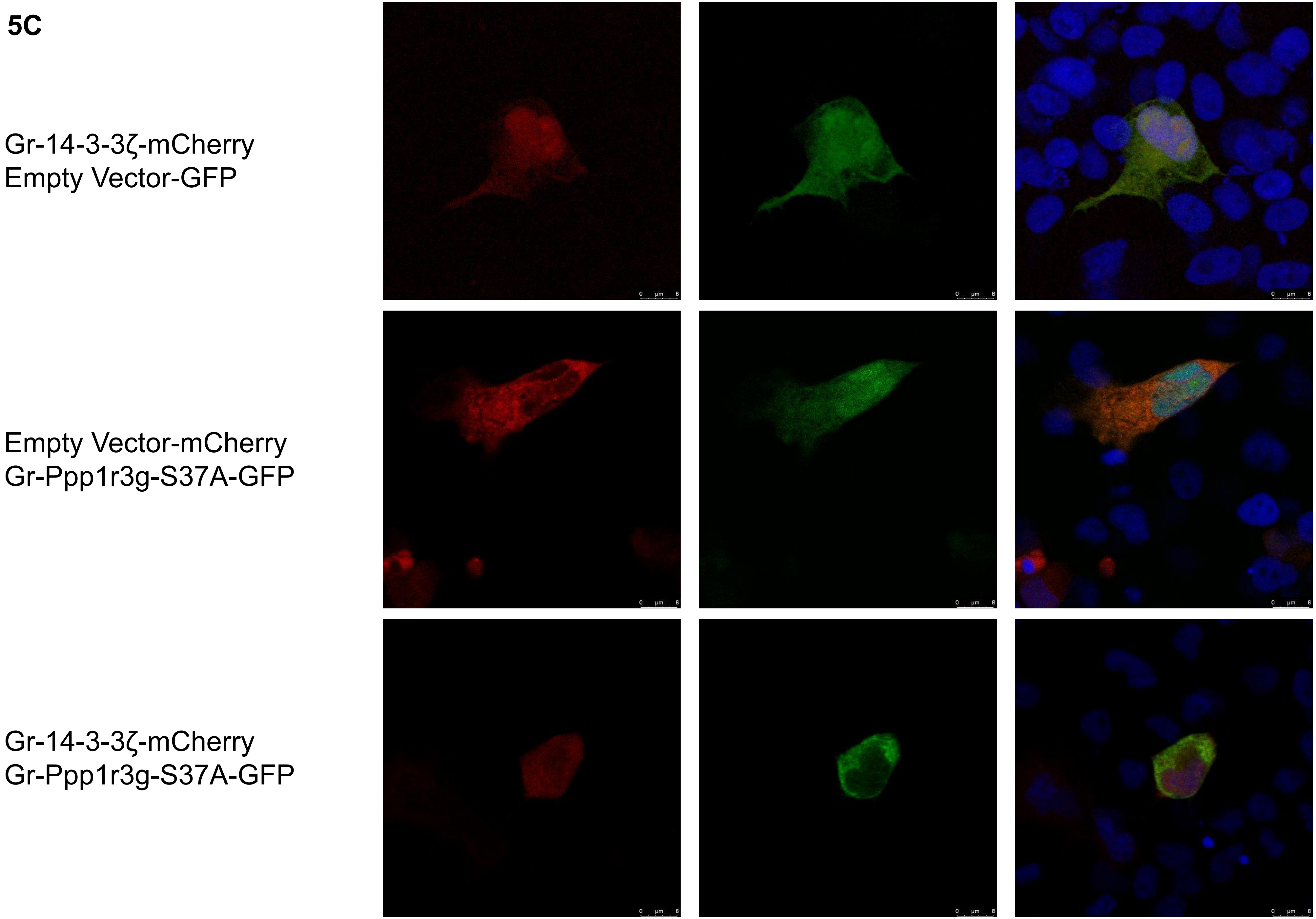
Gr-14-3-3ζ-mCherry
Empty Vector-GFP
Empty Vector-mCherry
Gr-Ppp1r3g-S37A-GFP
Gr-14-3-3ζ-mCherry
Gr-Ppp1r3g-S37A-GFP

Fig 5C

Ci-14-3-3ζ-mCherry
Empty Vector-GFP

Empty Vector-mCherry
Ci-Ppp1r3g-GFP

Ci-14-3-3ζ-mCherry
Ci-Ppp1r3g-GFP

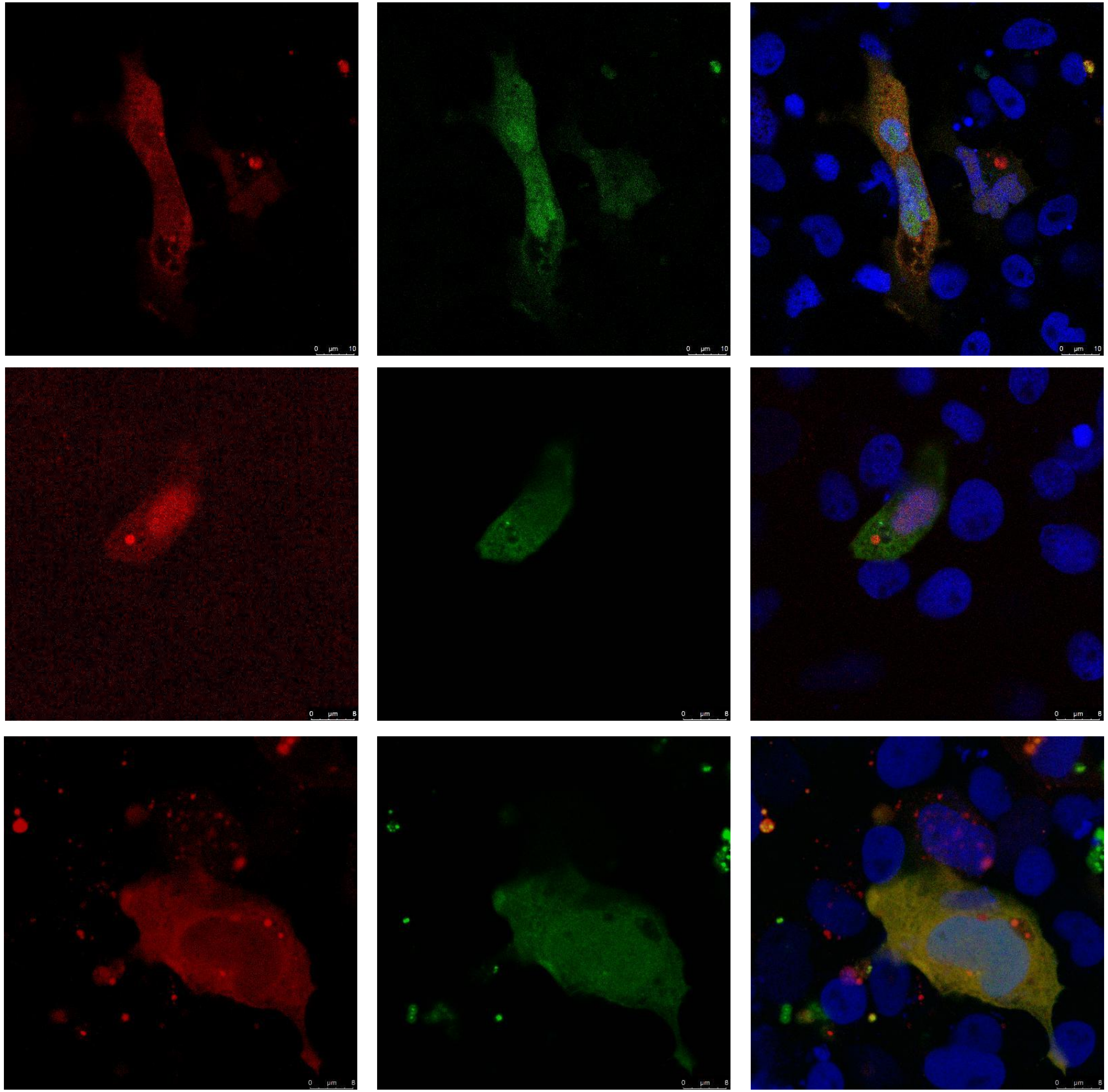

Fig 5C

Ci-14-3-3ζ-mCherry
Empty Vector-GFP

Empty Vector-mCherry
Ci-Ppp1r3g-S37A-GFP

Ci-14-3-3ζ-mCherry
Ci-Ppp1r3g-S37A-GFP

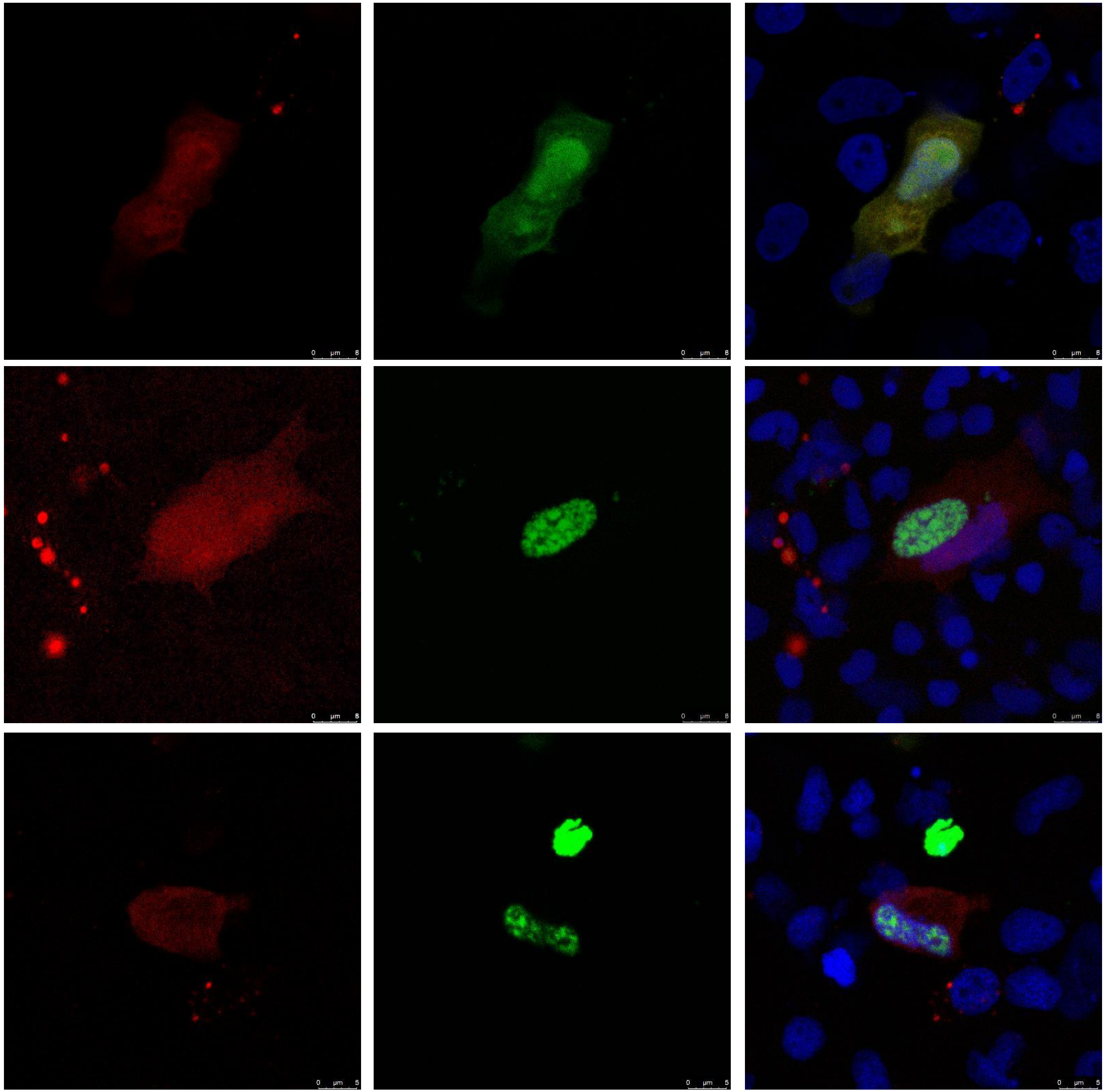

Fig 6D

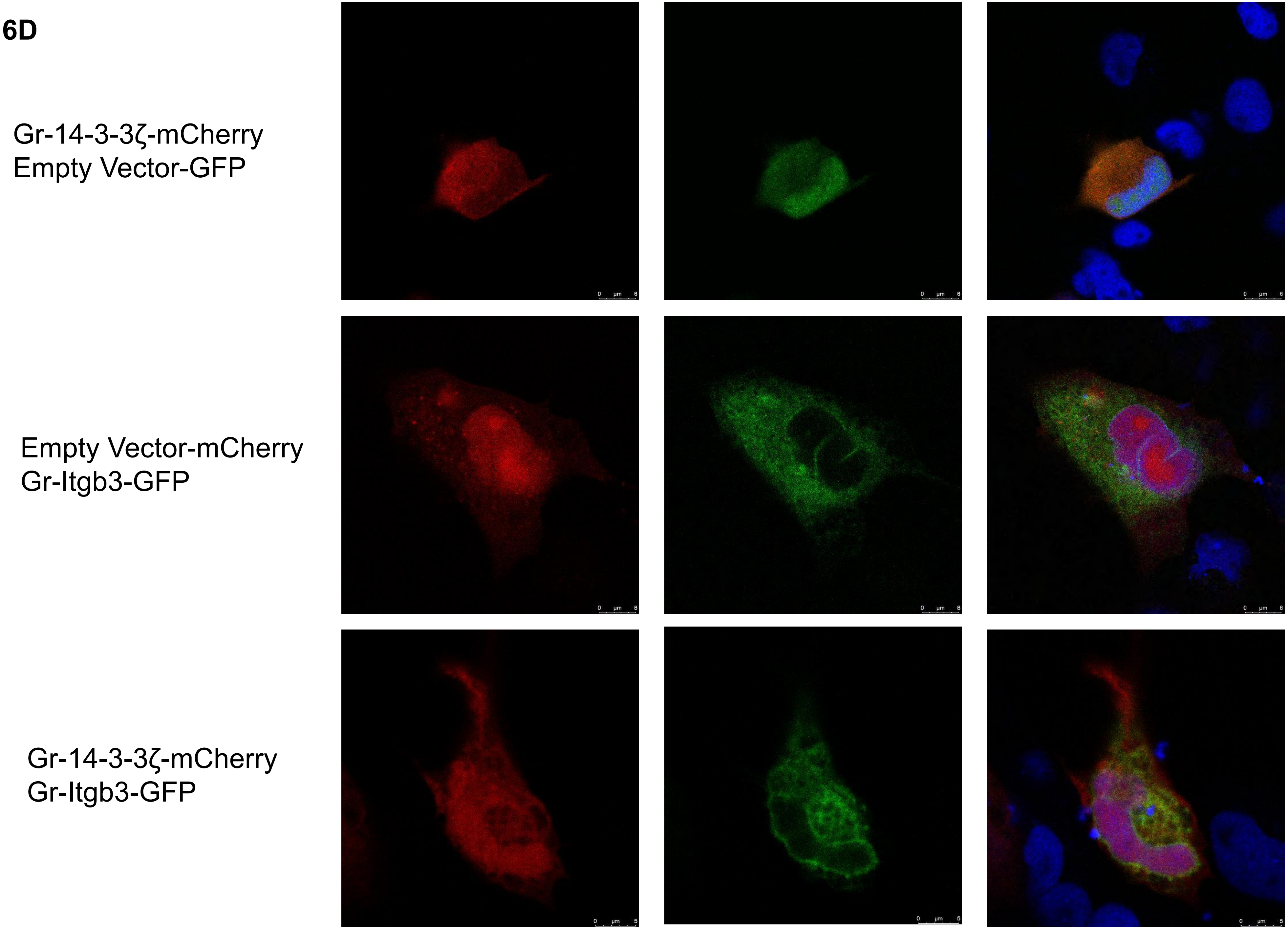
Gr-14-3-3ζ-mCherry
Empty Vector-GFP
Empty Vector-mCherry
Gr-Itgb3-GFP
Gr-14-3-3ζ-mCherry
Gr-Itgb3-GFP

Fig 6E

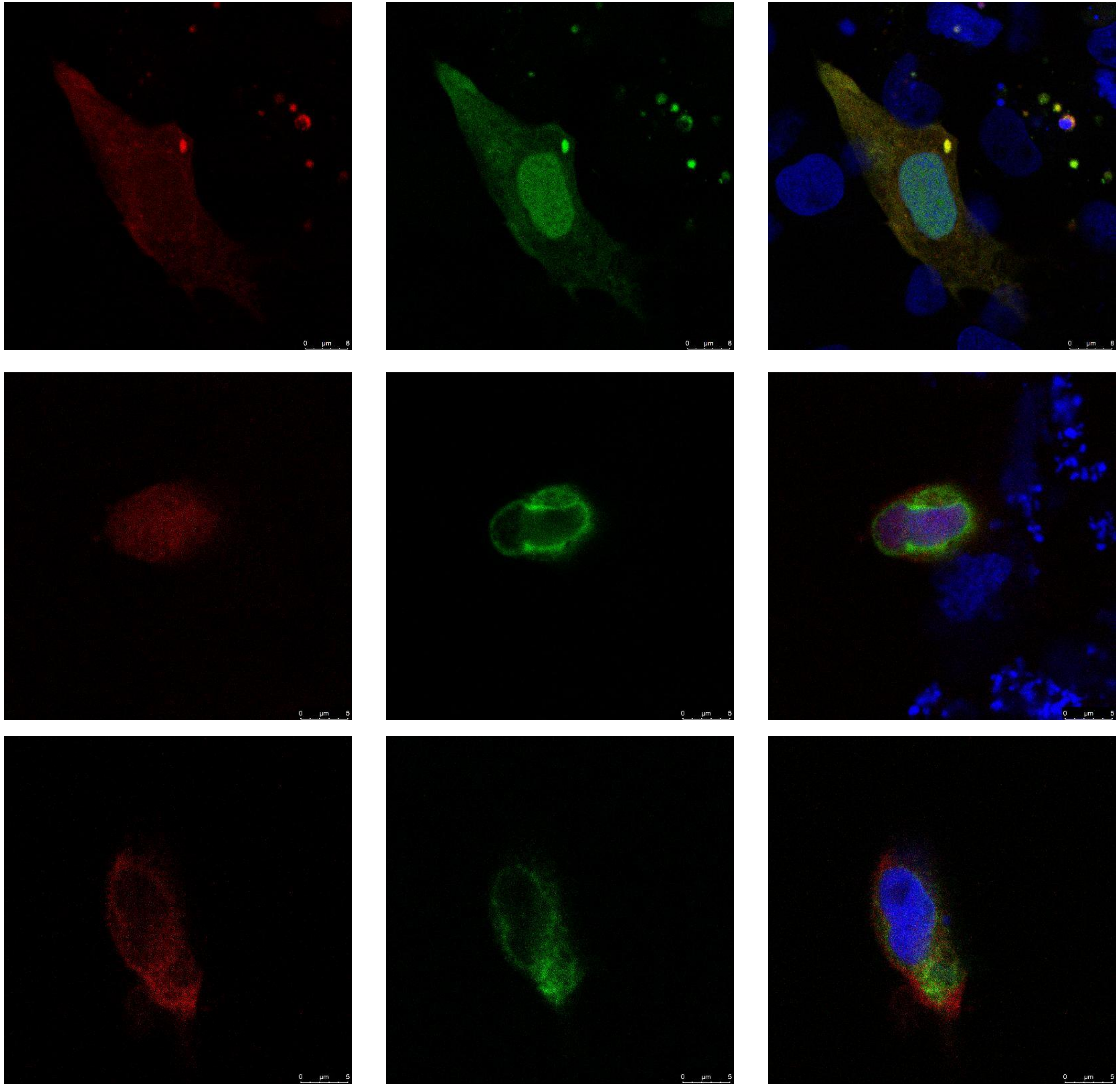
Ci-14-3-3ζ-mCherry
Empty Vector-GFP
Empty Vector-mCherry
Ci-Itgb3-GFP
Ci-14-3-3ζ-mCherry
Ci-Itgb3-GFP
