# Supplementary material for: Grass carp reovirus activates fish Ppp1r3g to suppress innate immunity and exacerbate hemorrhage via the 14-3-3ζ/Itgb3 signaling axis
Source: PLoS Pathog. 2026 Sep 18;22(9):e1014590. doi: 10.1371/journal.ppat.1014590 (PMC13588375; doi:10.1371/journal.ppat.1014590)
Supplement: Supplementary file 3 [file ppat.1014590.s010.docx]

| Primer | Sequence (5'-3') | Purpose |
| --- | --- | --- |
| Gr-*ppp1r3g*-K-F | ATGATGAACACAATGAATGAGG | ****cDNA cloning**** |
| Gr-*ppp1r3g*-K-R | TCATATCGCGTGGAAAGCCGCG |  |
| Gr-*14-3-3ζ*-K-F | ATGGACAGAACAGAGCTCAT |  |
| Gr-*14-3-3ζ*-K-R | TTTAGTTCTCTCCTCCCTCG |  |
| Gr-*14-3-3α*-K-F | ATGGATAAGACGGAGCTGAT |  |
| Gr-*14-3-3α*-K-R | TCAGTTCTCTCCTCCGTCTC |  |
| Gr-*itgb3*-K-F | ATGGAGAAAACTTCATCCAAACTTT |  |
| Gr-*itgb3*-K-R | TCAGTCCTTGCCTCGATATGTGATG |  |
| Gr-*ppp1r3g-*Ser37-K-F1 | AAGAGCCAAGGCCTTACCCGCGT |  |
| Gr-*ppp1r3g-*Ser37-K-R1 | ACGCGGGTAAGGCCTTGGCTCTT |  |
| Gr-*ppp1r3g-*Ser78-K-F1 | CTTCAGCATTGCCGAGGATCCTC |  |
| Gr-*ppp1r3g-*Ser78-K-R1 | GAGGATCCTCGGCAATGCTGAAG |  |
| Gr-*ppp1r3g-*Ser93-K-F1 | CAGGCTGAAGGCTTTCCCCCTTC |  |
| Gr-*ppp1r3g-*Ser93-K-R1 | GAAGGGGGAAAGCCTTCAGCCTG |  |
| Gr-*ppp1r3g-*PP1c-K-F | ATGATGAACACAATGAATGA |  |
| Gr-*ppp1r3g-*PP1c-K-R | AGGATCCTCGCTAATGCTGA |  |
| Gr-*ppp1r3g-*GS-K-F | GACTTGGAGACGCGGCTCGC |  |
| Gr-*ppp1r3g-*GS-K-R | TCATATCGCGTGGAAAGCCG |  |
| Gr-*14-3-3ζ-*EL17-K-F | TTGAGAACTCCACAAATCCC |  |
| Gr-*14-3-3ζ*-EL17-K-R | TCTCCTGCGGCAACTTCAGC |  |
| Gr-*itgb3-*▲P4-K-F | ATGGAGAAAACTTCATCCAA |  |
| Gr-*itgb3-*▲P4-K-R | TCAGTAGAGGGGGTTGTGTCCCG |  |
| Ci-*ppp1r3g*-K-F | ATGAACATAATGAATGAGGA |  |
| Ci-*ppp1r3g*-K-R | TCATATCGCGTGGAAAGCTG |  |
| Ci-*rig-i*-K-F | ATGTACGAGCTGGAAAAGGAGA |  |
| Ci-*rig-i*-K-R | TCAGTCTCTCAGCGGCCA |  |
| Ci-*traf6*-K-F | ATGGCTTGCAGCGACATGGA |  |
| Ci-*traf6*-K-R | TCAAAGTGAAGGTTCTGGGC |  |
| Ci-*mda5*-K-F | ATGAGTAGTGATCAGGACGC |  |
| Ci-*mda5*-K-R | CTACTCTGTTCTGTGAAATTCACAG |  |
| Ci-*14-3-3ζ*-K-F | ATTCACAGAACCAAGCAGCC |  |
| Ci-*14-3-3ζ*-K-R | GGTTCTTTGTAGAGTTGGCT |  |
| Ci-*14-3-3α*-K-F | ATGGACAAGAGCGACCTAGT |  |
| Ci-*14-3-3α*-K-R | TTAGTTCTCGCCCTCGCCGG |  |
| Ci-*itgb3*-K-F | ATGGAGAAAATTTCATCCAA |  |
| Ci-*itgb3*-K-R | TCAGTCCTTGCCTCGATATG |  |
| Ci-*ppp1r3g-*Ser36-K-F1 | AAGAGCCAAGGCCTTACCTGCGT |  |
| Ci-*ppp1r3g-*Ser36-K-R1 | ACGCAGGTAAGGCCTTGGCTCTT |  |
| Ci-*ppp1r3g-*Ser77-K-F1 | CTTCAGCACCGCCGAGGATCCTC |  |
| Ci-*ppp1r3g-*Ser77-K-R1 | GAGGATCCTCGGCGGTGCTGAAG |  |
| Ci-*ppp1r3g-*Ser92-K-F1 | CAGGCTGAAGGCTTTCCCTCTTC |  |
| Ci-*ppp1r3g-*Ser92-K-R1 | GAAGAGGGAAAGCCTTCAGCCTG |  |
| Ci-*ppp1r3g-*PP1c-K-F | ATGAACATAATGAATGAGGA |  |
| Ci-*ppp1r3g-*PP1c-K-R | AGGATCCTCGCTGGTGCTGA |  |
| Ci-*ppp1r3g-*GS-K-F | GCGCTGGAAAGCGTCACGGT |  |
| Ci-*ppp1r3g-*GS-K-R | TCATATCGCGTGGAAAGCTG |  |
| Ci-*14-3-3ζ-*EL17-K-F | TCCGCAACACTTCTCCAGCA |  |
| Ci-*14-3-3ζ-*EL17-K-R | TCTCCTGTGGCGACCTCTGC |  |
| Ci-*itgb3-*▲P4-K-F | ATGGAGAAAATTTCATCCAA |  |
| Ci-*itgb3-*▲P4-K-R | TCATTTGTAGAGGGGGTTGTGGC |  |
| Gr-*ppp1r3g*-EcoR Ⅰ-F | ATGGCCATGGAGGCCCGAATTCGGATGATGAACACAATGAATGAGG | **Plasmid Construction** |
| Gr-*ppp1r3g*- Sal Ⅰ-F | GCGGTACCTCGAGAGATCTCGGTCGACTCATATCGCGTGGAAAGCCGCG |  |
| Gr-*14-3-3ζ*-EcoR Ⅰ-F | ATGGCCATGGAGGCCCGAATTCGGATGGACAGAACAGAGCTCAT |  |
| Gr-*14-3-3ζ*- Sal Ⅰ-F | GCGGTACCTCGAGAGATCTCGGTCGACTTTAGTTCTCTCCTCCCTCG |  |
| Gr-*14-3-3α*-EcoR Ⅰ-F | ATGGCCATGGAGGCCCGAATTCGGATGGATAAGACGGAGCTGAT |  |
| Gr-*14-3-3α*- Sal Ⅰ-F | GCGGTACCTCGAGAGATCTCGGTCGACTCAGTTCTCTCCTCCGTCTC |  |
| Gr-*itgb3*-EcoR Ⅰ-F | ATGGCCATGGAGGCCCGAATTCGGATGGAGAAAACTTCATCCAAACTTT |  |
| Gr-*itgb3*- Sal Ⅰ-F | GCGGTACCTCGAGAGATCTCGGTCGACTCAGTCCTTGCCTCGATATGTGATG |  |
| Gr-*ppp1r3g-*Ser37-EcoR Ⅰ-F | ATGGCCATGGAGGCCCGAATTCGGATGATGAACACAATGAATGAGG |  |
| Gr-*ppp1r3g-*Ser37- Sal Ⅰ-R | GCGGTACCTCGAGAGATCTCGGTCGACTCATATCGCGTGGAAAGCCGCG |  |
| Gr-*ppp1r3g-*Ser78-EcoR Ⅰ-F | ATGGCCATGGAGGCCCGAATTCGGATGATGAACACAATGAATGAGG |  |
| Gr-*ppp1r3g-*Ser78- Sal Ⅰ-R | GCGGTACCTCGAGAGATCTCGGTCGACTCATATCGCGTGGAAAGCCGCG |  |
| Gr-*ppp1r3g-*Ser93-EcoR Ⅰ-F | ATGGCCATGGAGGCCCGAATTCGGATGATGAACACAATGAATGAGG |  |
| Gr-*ppp1r3g-*Ser93- Sal Ⅰ-R | GCGGTACCTCGAGAGATCTCGGTCGACTCATATCGCGTGGAAAGCCGCG |  |
| Gr-*ppp1r3g-*PP1c-EcoR Ⅰ-F | ATGGCCATGGAGGCCCGAATTCGGATGATGAACACAATGAATGA |  |
| Gr-*ppp1r3g-*PP1c- Sal Ⅰ-R | GCGGTACCTCGAGAGATCTCGGTCGACAGGATCCTCGCTAATGCTGA |  |
| Gr-*ppp1r3g-*GS-EcoR Ⅰ-F | ATGGCCATGGAGGCCCGAATTCGGGACTTGGAGACGCGGCTCGC |  |
| Gr-*ppp1r3g-*GS- Sal Ⅰ-R | GCGGTACCTCGAGAGATCTCGGTCGACTCATATCGCGTGGAAAGCCG |  |
| Gr-*14-3-3ζ-*EL17-EcoR Ⅰ-F | ATGGCCATGGAGGCCCGAATTCGGATGGACAGAACAGAGCTCAT |  |
| Gr-*14-3-3ζ*-EL17- Sal Ⅰ-R | GCGGTACCTCGAGAGATCTCGGTCGACTTTAGTTCTCTCCTCCCTCG |  |
| Gr-*itgb3-*▲P4-EcoR Ⅰ-F | ATGGCCATGGAGGCCCGAATTCGGATGGAGAAAACTTCATCCAA |  |
| Gr-*itgb3-*▲P4- Sal Ⅰ-R | GCGGTACCTCGAGAGATCTCGGTCGACTCAGTAGAGGGGGTTGTGTCCCG |  |
| Ci-*ppp1r3g*-EcoR Ⅰ-F | ATGGCCATGGAGGCCCGAATTCGGATGAACATAATGAATGAGGA |  |
| Ci-*ppp1r3g*- Sal Ⅰ-R | GCGGTACCTCGAGAGATCTCGGTCGACTCATATCGCGTGGAAAGCTG |  |
| Ci-*rig-i*-EcoR Ⅰ-F | ATGGCCATGGAGGCCCGAATTCGGATGTACGAGCTGGAAAAGGAGA |  |
| Ci-*rig-i*- Sal Ⅰ-R | GCGGTACCTCGAGAGATCTCGGTCGACTCAGTCTCTCAGCGGCCA |  |
| Ci-*traf6*-EcoR Ⅰ-F | ATGGCCATGGAGGCCCGAATTCGGATGGCTTGCAGCGACATGGA |  |
| Ci-*traf6*- Sal Ⅰ-R | GCGGTACCTCGAGAGATCTCGGTCGACTCAAAGTGAAGGTTCTGGGC |  |
| Ci-*mda5*-EcoR Ⅰ-F | ATGGCCATGGAGGCCCGAATTCGGATGAGTAGTGATCAGGACGC |  |
| Ci-*mda5*- Sal Ⅰ-R | GCGGTACCTCGAGAGATCTCGGTCGACCTACTCTGTTCTGTGAAATTCACAG |  |
| Ci-*14-3-3ζ*-EcoR Ⅰ-F | ATGGCCATGGAGGCCCGAATTCGGATGGACAAGAGCCAACATGT |  |
| Ci-*14-3-3ζ*- Sal Ⅰ-R | GCGGTACCTCGAGAGATCTCGGTCGACTTAGTTTTCCCGGCCCTCCT |  |
| Ci-*14-3-3α*-EcoR Ⅰ-F | ATGGCCATGGAGGCCCGAATTCGGATGGACAAGAGCGACCTAGT |  |
| Ci-*14-3-3α*- Sal Ⅰ-R | GCGGTACCTCGAGAGATCTCGGTCGACTTAGTTCTCGCCCTCGCCGG |  |
| Ci-*itgb3*-EcoR Ⅰ-F | ATGGCCATGGAGGCCCGAATTCGGATGGAGAAAATTTCATCCAA |  |
| Ci-*itgb3*- Sal Ⅰ-R | GCGGTACCTCGAGAGATCTCGGTCGACTCAGTCCTTGCCTCGATATG |  |
| Ci-*ppp1r3g-*Ser36-EcoR Ⅰ-F | ATGGCCATGGAGGCCCGAATTCGGATGAACATAATGAATGAGGA |  |
| Ci-*ppp1r3g-*Ser36- Sal Ⅰ-R | GCGGTACCTCGAGAGATCTCGGTCGACTCATATCGCGTGGAAAGCTG |  |
| Ci-*ppp1r3g-*Ser77-EcoR Ⅰ-F | ATGGCCATGGAGGCCCGAATTCGGATGAACATAATGAATGAGGA |  |
| Ci-*ppp1r3g-*Ser77- Sal Ⅰ-R | GCGGTACCTCGAGAGATCTCGGTCGACTCATATCGCGTGGAAAGCTG |  |
| Ci-*ppp1r3g-*Ser92-EcoR Ⅰ-F | ATGGCCATGGAGGCCCGAATTCGGATGAACATAATGAATGAGGA |  |
| Ci-*ppp1r3g-*Ser92- Sal Ⅰ-R | GCGGTACCTCGAGAGATCTCGGTCGACTCATATCGCGTGGAAAGCTG |  |
| Ci-*ppp1r3g-*PP1c-EcoR Ⅰ-F | ATGGCCATGGAGGCCCGAATTCGGATGAACATAATGAATGAGGA |  |
| Ci-*ppp1r3g-*PP1c- Sal Ⅰ-R | GCGGTACCTCGAGAGATCTCGGTCGACAGGATCCTCGCTGGTGCTGA |  |
| Ci-*ppp1r3g-*GS-EcoR Ⅰ-F | ATGGCCATGGAGGCCCGAATTCGGGCGCTGGAAAGCGTCACGGT |  |
| Ci-*ppp1r3g-*GS- Sal Ⅰ-R | GCGGTACCTCGAGAGATCTCGGTCGACTCATATCGCGTGGAAAGCTG |  |
| Ci-*14-3-3ζ-*EL17-EcoR Ⅰ-F | ATGGCCATGGAGGCCCGAATTCGGATGGACAAGAGCCAACATGT |  |
| Ci-*14-3-3ζ-*EL17- Sal Ⅰ-R | GCGGTACCTCGAGAGATCTCGGTCGACTTAGTTTTCCCGGCCCTCCT |  |
| Ci-*itgb3-*▲P4-EcoR Ⅰ-F | ATGGCCATGGAGGCCCGAATTCGGATGGAGAAAATTTCATCCAA |  |
| Ci-*itgb3-*▲P4- Sal Ⅰ-R | GCGGTACCTCGAGAGATCTCGGTCGACTCATTTGTAGAGGGGGTTGTGGC |  |
| Phage-puro-Gr-*ppp1r3g*-BamHI-F | AAGATCTGGCCCGGGCGGGATCCATGATGAACACAATGAATGA |  |
| Phage-puro-Gr-*ppp1r3g-*XholI-R | CGTCATCCTTGTAATCCTCGAGTCATATCGCGTGGAAAGCCG |  |
| Phage-puro-Ci-*ppp1r3g-*BamHI-F | AAGATCTGGCCCGGGCGGGATCCATGAACATAATGAATGAGGA |  |
| Phage-puro-Ci-*ppp1r3g-*XholI-R | CGTCATCCTTGTAATCCTCGAGTCATATCGCGTGGAAAGCTG |  |
| GFP-Gr-*ppp1r3g*-EcoR Ⅰ-F | AGCTCAAGCTTCGAATTCTGATGATGAACACAATGAATGA |  |
| GFP-Gr-*ppp1r3g*-Sal Ⅰ-R | GGGCCCGCGGTACCGTCGACTGTATCGCGTGGAAAGCCGCGG |  |
| GFP-Ci-*ppp1r3g*-EcoR Ⅰ-F | AGCTCAAGCTTCGAATTCTGATGAACATAATGAATGAGGA |  |
| GFP-Ci-*ppp1r3g*-Sal Ⅰ-R | GGGCCCGCGGTACCGTCGACTGTATCGCGTGGAAAGCTGCGG |  |
| mCherry-Gr-*14-3-3ζ*-EcoR Ⅰ-F | ACTCAGATCTCGAGCTCAAGCTTCGAATTCATGGACAGAACAGAGCTCAT |  |
| mCherry-Gr-*14-3-3ζ*-Sal Ⅰ-R | GGGCCCGCGGTACCGTCGACTGGTTCTCTCCTCCCTCGCCTC |  |
| mCherry-Ci-*14-3-3ζ*-EcoR Ⅰ-F | ACTCAGATCTCGAGCTCAAGCTTCGAATTCATGGACAAGAGCCAACATGT |  |
| mCherry-Ci-*14-3-3ζ*-Sal Ⅰ-R | GGGCCCGCGGTACCGTCGACTGGTTTTCCCGGCCCTCCTCTG |  |
| GFP-Gr-*itgb3*-EcoR Ⅰ-F | AGCTCAAGCTTCGAATTCTGATGGAGAAAACTTCATCCAAACTTT |  |
| GFP-Gr-*itgb3*-Sal Ⅰ-R | GGGCCCGCGGTACCGTCGACTGGTCCTTGCCTCGATATGTGA |  |
| GFP-Ci-*itgb3*-EcoR Ⅰ-F | AGCTCAAGCTTCGAATTCTGATGGAGAAAATTTCATCCAA |  |
| GFP-Ci-*itgb3*-Sal Ⅰ-R | GGGCCCGCGGTACCGTCGACTGGTCCTTGCCTCGATATGTGA |  |
| Gr-*ppp1r3*g-RT-F | TTCAAGATGCCCGTGGACTC | **qRT-PCR** |
| Gr-*ppp1r3*g-RT-R | CGACAGCCAGTCGTTGAAAG |  |
| Gr-*irf3*-RT-F | ACAGCAAGGTTTGGAGGGAC |  |
| Gr-*irf3*-RT-F | AATCTGGCCCAAACCACCAA |  |
| Gr-*mx*-RT-F | CAACCGGACAAAGGACTCTGGG |  |
| Gr-*mx*-RT-R | GATGTCTTGCTGGCCTCTGCAC |  |
| Gr-*tlr3*-RT-F | CACTTCACCAGGTAATGGAGGAC |  |
| Gr-*tlr3*-RT-R | TGTTGGTAGAGGCTAATGCGGA |  |
| Gr-*14-3-3ζ*-RT-F | GGGATTTGTGGAGTTCTCAA |  |
| Gr-*14-3-3ζ*-RT-R | GCTGAAGTTGCCGCAGGAGA |  |
| Gr-*14-3-3α*-RT-F | AAGAAGGAGATGCAGCCCAC |  |
| Gr-*14-3-3α*-RT-R | CAGCGTTGTCAGAGGTCCAT |  |
| Gr-*itgb3*-RT-F | CGACAGTCCCTTCTGTCACC |  |
| Gr-*itgb3*-RT-R | CACACTCACACCTCTTCCCC |  |
| Gr-*f2*-RT-F | GGACCTCAAACCCCACAAATC |  |
| Gr-*f2*-RT-R | CCCACGACACAATGCCAATC |  |
| Gr-*f3a*-RT-F | CAGGCCTACCTGCCCTTCC |  |
| Gr-*f3a*-RT-R | TTTTTCGCTCGCTTTCTTCG |  |
| Gr-*f3b*-RT-F | TTTTCGGTGCTTTTTCTCGC |  |
| Gr-*f3b*-RT-R | TCTGTTTGTCCCTACCAACTCTTG |  |
| Gr-*f10*-RT-F | AGTAAAGTTGCCACACAGGGAGC |  |
| Gr-*f10*-RT-R | CAGTTGGGTAGTTGTAAGAATGTAAAGG |  |
| Gr-*kng-1*-RT-F | ACCGCTCATTCATTCTTTGGC |  |
| Gr-*kng-1*-RT-R | CCTGGTGCAGTTGCTCTTCTC |  |
| Gr-*serpinb1*-RT-F | TCTCTGATGTTCACTCTCGGTTTG |  |
| Gr-*serpinb1*-RT-R | TCTTTGATTTTATTCTCCGTCTGC |  |
| Gr-*serpinc1*-RT-F | TCGATAAAGGAGAAGACATCTGACC |  |
| Gr-*serpinc1*-RT-R | GCTGGAAGGACTCATTAAAAAGTGTAG |  |
| Gr-*serpind1*-RT-F | GCAGAACTATGACTTGATTGACCAC |  |
| Gr-*serpind1*-RT-R | TGATAAAGGCATGAATCCGATGT |  |
| Gr-*serpinf1*-RT-F | CTCTCCAATACTCTCCACGCAGT |  |
| Gr-*serpinf1*-RT-R | CGCTTCCAACCATTCAGACATAC |  |
| Gr-*serpinf2b*-RT-F | ACCTTGATACACTCTGTGATGGGG |  |
| Gr-*serpinf2b-*RT-R | GGAAATTATGACATTGGGCTGTT |  |
| Gr-*serping1*-RT-F | TTCCTCTCAGACACTGTTGGTGG |  |
| Gr-*serping1*-RT-R | GAAGATCAAGTTGGATTTGGGGT |  |
| Gr-*β-actin*-RT-F | GTCCGTGACATCAAAGAG |  |
| Gr-*β-actin*-RT-R | ACCGCAAGATTCCATAC |  |
| Ci-*14-3-3α*-RT-F | GCAAAAGGGAAATGCAGCCA |  |
| Ci-*14-3-3α*-RT-R | CCACAGAGTGAGGTTGTCCC |  |
| Ci-*14-3-3ζ*-RT-F | ACTGACCGACGAGGAGAGAA |  |
| Ci-*14-3-3ζ*-RT-R | AACTTGTCCAGGAGGTGCAG |  |
| Ci-*itgb3*-RT-F | ACTTGTCTCAGTTGCGGAGG |  |
| Ci-*itgb3*-RT-R | CCCTGTTCCGAGACACCTTC |  |
| Ci-*ifn1*-RT-F | TGTTGGTAGAGGCTAATGCGGA |  |
| Ci-*ifn1*-RT-R | GCGTCCTGGAAATGACACCT |  |
| Ci-*ifn2*-RT-F | TCTTTTTCCTCGTGAATGCTTG |  |
| Ci-*ifn2*-RT-R | TCACAACGATGTTCTGACTGGA |  |
| Ci-*ifn3*-RT-F | TACATTTATAGAGACTGCGGGTGG |  |
| Ci-*ifn3*-RT-R | TGGAGTGTCTGGTAAACAGCCTT |  |
| Ci-*ifn4*-RT-F | GTTCGTCATTCAGGCTCTGGTAG |  |
| Ci-*ifn4*-RT-R | TCCCTCCATCCTCCTTGTTCA |  |
| Ci-*isg15*-RT-F | CCCCTTTCCAAGTGTTCGTC |  |
| Ci-*isg15*-RT-R | ATGGTGCTTCCAGATGTGATGT |  |
| Ci-*ifi56*-RT-F | TCTGGAGGGACTGAAGATTGGT |  |
| Ci-*ifi56*-RT-R | TGCGTTCGTTTCGTTCTTGTAG |  |
| GCRV Ⅰ-*vp2*-F | ATCAAGGATCCCATTCCGCCTTCA |  |
| GCRV Ⅰ-*vp2*-R | TTAGAGGATCGTGCCATTGAGGGT |  |
| GCRV Ⅰ-*vp7*-F | ACCACCAACTTTGATCACGCTGAG |  |
| GCRV Ⅰ-*vp7*-R | AGCGTGGGAGTCTTGAATGGTCTT |  |
| GC- *f2*-RT-F | ACCCTCCGCCACCTCCTAT |  |
| GC-*f2*-RT-R | GGGACCCTCCATATCTCCATCT |  |
| GC-*f3a*-RT-F | CGCCTCAGACACCATTCCAA |  |
| GC-*f3a*-RT-R | CAGGTCGCATTCAGTTTCCATC |  |
| GC- *f3b*-RT-F | AGGAGACAAGAGTTTAGGAGCGTG |  |
| GC-*f3b*-RT-R | CAGCACACCACAATCAGAACGA |  |
| GC-*f7*-RT-F | GAGGTCTCGTATCGTCGGTGG |  |
| GC-*f7*-RT-R | TGCGGTCTCGGACACATAGC |  |
| GC-*f9*-RT-F | TCGGATGCTGCTAAAGACGC |  |
| GC-*f9*-RT-R | ACTCCTTTAGTAACGCCCACCAC |  |
| Ci-*β-actin*-RT-F | AGCCATCCTTCTTGGGTATG |  |
| Ci-*β-actin*-RT-R | GGTGGGGCGATGATCTTGAT |  |
| Ci-*rig-i*-RT-F | ACTACACTGAACACCTGCGGAA |  |
| Ci-*rig-i*-RT-R | GCATCTTTAGTGCGGGCG |  |
| Ci-*ifn3-i*-RT-F | TACATTTATAGAGACTGCGGGTGG |  |
| Ci-*ifn3*-RT-R | TGGAGTGTCTGGTAAACAGCCTT |  |
| Ci-*ifn-γ2*-RT-F | CAGCGAACACCTGAAACTAACA |  |
| Ci-*ifn-γ2*-RT-R | CCATCCCAAAGTCATCAAACAT |  |
| Ci-*isg15*-RT-F | CCCCTTTCCAAGTGTTCGTC |  |
| Ci-*isg15*-RT-R | ATGGTGCTTCCAGATGTGATGT |  |
